# Supplementary material for: Datagraphy: toward a systematic approach to dataset discovery
Source: Gigascience. 2025 Oct 22;14:giaf134. doi: 10.1093/gigascience/giaf134 (PMC12644982; doi:10.1093/gigascience/giaf134)

GigaScience

Datagraphy: toward a systematic approach to dataset discovery

--Manuscript Draft--

**Manuscript Number:**	GIGA-D-25-00204
**Full Title:**	Datagraphy: toward a systematic approach to dataset discovery
**Article Type:**	Research
**Funding Information:**	Agence Nationale de la Recherche (ANR-23-IACL-0006) — Not applicable<br>Agence Nationale de la Recherche (ANR-10-AIRT-05) — Not applicable<br>Agence Nationale de la Recherche (ANR-15-IDEX-02) — Not applicable
**Abstract:**	Data has become central to scientific discovery. While primary data collection remains vital, there is growing recognition of the benefits of reusing existing datasets. However, identifying suitable datasets for specific research questions is increasingly difficult due to the fragmentation and heterogeneity of the big data ecosystem. Despite the expansion of data sharing, efficient data discovery remains elusive, with limited empirical research on how datasets are identified, interpreted, and reused. Current dataset search practices often lack standardization, leading researchers to rely on convenience rather than systematic criteria. Unlike bibliographic research, dataset selection lacks a formal methodology, increasing the risks of bias, inefficiencies, and reduced generalizability. To address this gap, we introduce datagraphy, a structured approach to dataset identification and evaluation. Analogous to bibliographic methods but designed for datasets, datagraphy encompasses not only discovery but also critical assessment of data quality, relevance, interoperability, completeness, sustainability, and ethical use. By formalizing dataset search as a research practice, datagraphy seeks to improve transparency, reproducibility, and interdisciplinary collaboration, while also reducing research redundancy and environmental impact. We present a nine-step framework to operationalize datagraphy and explore challenges such as inconsistent metadata and variability among data discovery tools. This framework provides a foundation for systematically and reproducibly identifying and synthesizing reusable datasets. To demonstrate the application of the proposed framework, we conducted a datagraphic search focused on the exposome. Standardization, AI-assisted automation, and community engagement will be key to establishing datagraphy as a foundational research practice, ultimately strengthening the integrity and impact of data-driven science.
**Corresponding Author:**	Pascal Petit, Ph.D.<br>Universite Grenoble Alpes<br>La Tronche Cedex, FRANCE
**Corresponding Author Secondary Information:**	
**Corresponding Author's Institution:**	Universite Grenoble Alpes
**Corresponding Author's Secondary Institution:**	
**First Author:**	Pascal Petit, Ph.D.
**First Author Secondary Information:**	
**Order of Authors:**	Pascal Petit, Ph.D.
	Nicolas Vuillerme, PhD, HDR
**Order of Authors Secondary Information:**	
**Additional Information:**	
**Question**	**Response**
Are you submitting this manuscript to a	No

Powered by Editorial Manager® and ProduXion Manager® from Aries Systems Corporation

special series or article collection?	
**Experimental design and statistics**<br><br>Full details of the experimental design and statistical methods used should be given in the Methods section, as detailed in our Minimum Standards Reporting Checklist. Information essential to interpreting the data presented should be made available in the figure legends.<br><br>Have you included all the information requested in your manuscript?	Yes
**Resources**<br><br>A description of all resources used, including antibodies, cell lines, animals and software tools, with enough information to allow them to be uniquely identified, should be included in the Methods section. Authors are strongly encouraged to cite Research Resource Identifiers (RRIDs) for antibodies, model organisms and tools, where possible.<br><br>Have you included the information requested as detailed in our Minimum Standards Reporting Checklist?	Yes
**Availability of data and materials**<br><br>All datasets and code on which the conclusions of the paper rely must be either included in your submission or deposited in publicly available repositories (where available and ethically appropriate), referencing such data using a unique identifier in the references and in the "Availability of Data and Materials" section of your manuscript.<br><br>Have you have met the above requirement as detailed in our Minimum Standards Reporting Checklist?	Yes

Powered by Editorial Manager® and ProduXion Manager® from Aries Systems Corporation

GigaScience has policies and guidelines in place for the use of generative AI-writing tools such as ChatGPT. If you have used such writing tools to assist with writing the manuscript this must be declared and cited in the text. Authors should not list AI-writing tools and other AI-assisted technologies as an author or co-author and should acknowledge that they are fully responsible for text generated or refined by AI-writing tools.<p><br><br>A summary of use (particularly in the introduction or among methods) needs to be included at the end of the paper, and the outputs should also be included as a supplementary file hosted in GigaDB or other open repositories. Please read our guidelines for more information. <p><br><br>By submitting to GigaScience, you are aware of the journal's AI-writing tools policy, and if you have declared use of such tools below, you have acknowledged this where appropriate in your manuscript and have made a summary of use and outputs available. <p><br><b>AI-assisted writing tools have been used in the preparation of this manuscript?	No

Powered by Editorial Manager® and ProduXion Manager® from Aries Systems Corporation

Manuscript
Click here to access/download;Manuscript;Datagraphy_GigaScience_26-05-

Title: Datagraphy: toward a systematic approach to dataset discovery

Authors

Pascal Petit[1,*]; Nicolas Vuillerme[1,2]

[1] Univ. Grenoble Alpes, AGEIS, 38000 Grenoble, France

[2] Institut Universitaire de France, Paris, France

Correspondence (present address)

* Corresponding author at: Pascal Petit. Laboratoire AGEIS - Université Grenoble Alpes. Bureau 315. Bâtiment Jean Roget. UFR de Médecine. Domaine de La Merci. 38706 La Tronche Cedex, France. E-mail address: pascal.petit@univ-grenoble-alpes.fr (P. Petit). Telephone: + 33 4 76 63 71 04.

ORCID

PP: 0000-0001-9015-5230. NV: 0000-0003-3773-393X.

Abstract

Data has become central to scientific discovery. While primary data collection remains vital, there is growing recognition of the benefits of reusing existing datasets. However, identifying suitable datasets for specific research questions is increasingly difficult due to the fragmentation and heterogeneity of the big data ecosystem. Despite the expansion of data sharing, efficient data discovery remains elusive, with limited empirical research on how datasets are identified, interpreted, and reused. Current dataset search practices often lack standardization, leading researchers to rely on convenience rather than systematic criteria. Unlike bibliographic research, dataset selection lacks a formal methodology, increasing the risks of bias, inefficiencies, and reduced generalizability. To address this gap, we introduce datagraphy, a structured approach to dataset identification and evaluation. Analogous to bibliographic methods but designed for datasets, datagraphy encompasses not only discovery but also critical assessment of data quality, relevance, interoperability, completeness, sustainability, and ethical use. By formalizing dataset search as a research practice, datagraphy seeks to improve transparency, reproducibility, and interdisciplinary collaboration, while also reducing research redundancy and environmental impact. We present a nine-step framework to operationalize datagraphy and explore challenges such as inconsistent metadata and variability among data discovery tools. This framework provides a foundation for systematically and reproducibly identifying and synthesizing reusable datasets. To demonstrate the application of the proposed framework, we conducted a datagraphic search focused on the exposome. Standardization, AI-assisted automation, and community engagement will be key to establishing datagraphy as a foundational research practice, ultimately strengthening the integrity and impact of data-driven science.

Keywords: dataset discovery; data reuse; datagraphy; datagraphic search; research practice; information; exposome; big data; open data; open science

1. Background

Data is omnipresent, shaping nearly every aspect of our lives [1]. The digital revolution and the increasing reliance on technology have led to an exponential surge in data generation, creating some kind of "data big bang." This transformation has marked the onset of the fourth industrial revolution, in which information has become a central pillar and data is widely regarded as the "new gold" of the 21st century [2,3]. Over the past years, data has evolved from being a mere byproduct of digital activities to a valuable asset whose value grows with use [2]. The web now provides access to millions of data sources [2], which are becoming increasingly vast, complex, and heterogeneous as societies undergo continuous digitization [4]. These sources originate from diverse domains and are of different natures [5], including contextual data (e.g., air pollution), person-generated data (e.g., wearables, social media), administrative health data (e.g., electronic health records), and synthetic data (e.g., digital twins) [6,7]. This data revolution is profoundly reshaping the scientific landscape.

In recent years, the scientific field has undergone an epistemological shift, transitioning from knowledge-driven to data-driven research [1,4,8]. Data has become a cornerstone of scientific discovery and is increasingly regarded as a form of scientific currency (data commodification) [4,8-10]. This shift has driven efforts to move away from isolated data silos towards more integrated, accessible, and reusable data ecosystems, where multiple data sources can be used or needed to address a research question. To support this transition, major efforts have been made to establish mandates and standards promoting data sharing [11-13], including the adoption of the FAIR (Findable, Accessible, Interoperable, and Reusable) principles [2,14-16]. The ultimate goal of the FAIR principles is to optimize the reuse of digital assets populating the Internet [2,14-16].

While the collection of new data remains a central focus, there is increasing recognition of the substantial benefits associated with reusing existing datasets [11,17,18]. For example, the European Union and the World Health Organization (WHO) have introduced supportive frameworks such as the Research Data Alliance (RDA) [19], the European Open Data Directive (Directive (EU) 2019/1024), the Data Governance Act (DGA) [20,21], the General Data Protection Regulation (GDPR) [20,22], and the European Health Data Space [20,22]. Nearly half of researchers frequently use data generated by other scientists [24]. Data reuse not only facilitates the validation and replication of findings but also enables

the exploration of extended or novel research questions [17,18]. When disseminated with sufficient quality and contextual information, datasets from diverse scientific communities can contribute to new knowledge through cross-disciplinary insights. Data reuse has become an established practice that mitigates unnecessary duplication of research, accelerates scientific progress, and optimizes resource allocation in terms of time, effort, staff, and costs, while limiting risks to research participants [9,11,17,18]. In this regard, the reuse of data represents a resource-efficient approach that helps reduce the environmental footprint of research [25,26]. Furthermore, leveraging existing datasets allows researchers to address high-impact scientific questions that would otherwise require considerable resources and time [27].

Beyond access, data reuse relies on effective data discovery [14], a fundamental aspect of the FAIR principles that involves identifying and locating relevant datasets [14-16]. The ability to find appropriate datasets is a prerequisite for their reuse [28]. However, locating relevant data remains challenging, often requiring researchers to navigate multiple resources, review numerous publications, and directly contact data owners or study authors [13,14,28]. As data sharing becomes more common, the complexity of data discovery is becoming increasingly evident [10,14]. Traditionally, researchers identified relevant data by consulting the literature, attending conferences, and engaging with colleagues [10]. In today's data-rich environment, web searches have become the primary method for locating datasets [10], but other resources also exist. However, the success of such searches varies widely, depending on the expertise of the researcher, the tools employed, and, to some extent, chance [10]. Despite the growing need for efficient data discovery, there is still limited large-scale empirical evidence on how researchers locate, access, interpret, and reuse datasets [29]. Consequently, the importance of improving data discovery within the scientific community is paramount.

For decades, scientific research has relied on bibliographic searches to systematically identify and synthesize relevant literature, which is an essential process for supporting hypotheses, contextualizing findings, and ensuring methodological rigor [30,31]. However, in the era of data-driven science [19,32], the ability to select appropriate datasets is equally critical for research validity and reproducibility. Despite the increasing availability of large-scale, open-access datasets across scientific disciplines, researchers still lack standardized methodologies for systematically identifying, evaluating, and

selecting datasets that best address their specific research questions [6]. To bridge this gap, we introduce the concept of "*datagraphy*" (or "*datagraphic search*"), a structured approach analogous to bibliographic research but focused on datasets rather than publications. Here, we highlight the potential of datagraphy as well as hurdles that need to be overcome.

2. Defining datagraphy

We propose to define datagraphy as the systematic process of identifying, evaluating, and documenting datasets most suitable for addressing specific research questions, akin to how bibliographic research is conducted to identify relevant scientific literature. This process extends beyond simple dataset discovery; it further advantageously incorporates the assessment of data quality, relevance, completeness, and ethical considerations, ensuring that selected datasets align with research objectives in a rigorous and transparent manner. By formalizing datagraphy as a research practice, we advocate to enhance reproducibility, mitigate selection bias, and improve the overall integrity of data reuse investigations.

3. Rationale and existing gaps

A variety of resources are already available for conducting datagraphy (Table 1). Existing datasets are dispersed across an increasing number of repositories, article supplements, academic journals, websites, and other platforms, each employing distinct metadata structures, data standards, and search functionalities [14]. For instance, domain-agnostic dataset search engines, such as *Google Dataset Search*, enable broad searches before directing users to specific repositories, where more targeted queries and data exploration can be conducted [29,33,34]. Data catalogs [13], such as *OccupationalCohorts.net* [35] and *OccupationalExposureTools.net* [36], which provide structured inventories of data assets through curated metadata records. In addition to repositories and catalogs, data papers [37,38] serve as valuable resources for dataset discovery [39]. These scientific publications detail dataset collection, processing, and validation methodologies, thereby informing the research community about their availability, characteristics, and reuse potential [18]. Data papers undergo peer review and are published in both general scientific journals and specialized data journals, such as *GigaScience*, *Data*

in Brief, and *Scientific Data* [18,40]. To improve data accessibility, many traditional scientific journals now require data availability statements and mandate that datasets be stored either as supplementary materials or in designated repositories [24]. Numerous data repositories exist [41], including *Re3Data* [14,42,43], *Zenodo* [16,40], and *Dataverse* [17]. Government agencies also provide access to datasets, such as those available through the *NYU Libraries Data Sources* [13,34] and other national or regional data portals [2,44]. In addition, open platforms like *GitHub* and *Kaggle* host a range of datasets across multiple domains [2]. All of the aforementioned resources vary in scope, ranging from institutional (e.g., university-level) to international initiatives [28]. Some are domain-specific, such as *TEDI* for toxicology and public health [45], while others, like *Re3Data*, span multidisciplinary research areas [14,42,43] (Table 1). Access to these resources also differs, with some that are openly available (e.g., *Dataverse*), whereas others require institutional affiliation (e.g., *Web of Science*) or subscription-based access (e.g., *Dimensions.ia*).

While these resources facilitate dataset discovery, they primarily function as search engines rather than providing systematic evaluation frameworks. In contrast, bibliographic research methodologies, such as systematic reviews and meta-analyses, follow rigorous protocols for literature selection and synthesis [31]. Current dataset search strategies lack standardization, as researchers often select datasets based on convenience and opportunity rather than through systematic assessment. Unlike bibliographic research, to the best of our knowledge, no formalized methodology exists for dataset selection, making dataset integration and comparison particularly challenging [18]. The absence of structured dataset selection methodologies increases the risk of opportunistic dataset use, potentially introducing biases and limiting the generalizability of research findings. Developing a standardized framework for dataset identification and evaluation is therefore essential to enhancing the reliability and impact of reuse data research.

Table 1: Examples of digital searchable dataset discovery resources

Name	Type	Country	Domain	RA	DL	Characteristics
4TU.ResearchData	Data repository	International	Multidisciplinary	FA	yes	10 322 datasets
CANUE (Canadian Urban Environmental Health Research Consortium)	Data portal	Canada	Environmental health	FR	yes	
DANS	Data repository	Netherlands	Multidisciplinary	FA	yes	313 603 datasets
Data	Data-focused journal	International	Multidisciplinary	OA	yes	
Data Citation Index	Dataset aggregator	International	Multidisciplinary	Sub	?	>15 million datasets, 453 repositories
Data Europa	Governmental data portal	EU	Multidisciplinary	FA	yes	1 857 283 datasets, 195 catalogues
Data in Brief	Data-focused journal	International	Multidisciplinary	OA	yes	
Data Repository Finder	Data repository	International	Multidisciplinary	FA	no	25 repositories
Data Science	Data-focused journal	International	Multidisciplinary	OA	yes	
Data Science Journal – Codata	Data-focused journal	International	Multidisciplinary	OA	yes	
data.gouv	Governmental data portal	France	Multidisciplinary	FA	yes	61 418 datasets
data.world	Data catalog	International	Multidisciplinary	Sub	?	
Datacite	Data repository catalog	International	Multidisciplinary	FA	no	>20 million datasets, 3309 repositories
DataHub	Data catalog	International	Multidisciplinary	FA	yes	
DataMed	Open source data discovery system	International	Biomedical	FA	no	1 280 165 datasets, 49 repositories
DataOne	Data aggregator	International	Environment	FA	no	
Dataverse	Data repository	International	Multidisciplinary	FA	no	466 000 datasets
Dimensions.ia	Publication aggregator with dataset filter	International	Multidisciplinary	Sub	?	29 million datasets
Dryad	Data platform	International	Multidisciplinary	FA	yes	50 000 data publications
Earth data NASA	Data repository	International	Environment	FA	yes	10 749 datasets
EMIF Catalogue	Data catalog	EU	Health	FR	?	480 datasets
Environmental Data Initiative (EDI) Repository	Data repository	International	Environment	FA	yes	
Portail Epidémiologie – France	Data catalog	France	Public health	FA	no	1 098 datasets
EUDat	Data repository	EU	Multidisciplinary	FA	no	
European Health Data Space (EHDS)	Data platform	EU	Health	FA	?	
European Open Science Cloud (EOSC)	Data platform	EU	Multidisciplinary	FA	?	
F1000Research	Data-focused journal	International	Multidisciplinary	OA	yes	
FAIR environmental and health registry (FAIREHR)	Data registry	International	Public health, environmental health	FA	no	
FAIRDOM	Data platform	International	Biology	FR	?	
FAIRsharing	Data catalog	International	Multidisciplinary	FA	no	2 318 datasets
FigShare	Data repository	International	Multidisciplinary	FA	yes	2 107 300
GigaScience	Data-focused journal	International	Multidisciplinary	OA	yes	
GitHub	Data platform	International	Multidisciplinary	FA	yes	
Google dataset search	Domain agnostic data search engine	International	Multidisciplinary	FA	no	>25 million datasets
Green data for health (GD4H)	Data catalog	France	Environment, environmental health	FA	no	177 datasets
Harvard Data Science Review	Data-focused journal	International	Multidisciplinary	OA	yes	
Kaggle	Data platform	International	Multidisciplinary	FR	yes	438 322 datasets
Mendeley Data	Data aggregator	International	Multidisciplinary	FA	yes	>20 million datasets
NYU Data Catalog	Governmental data portal	US	Health	FA	no	426 datasets
OccupationalCohorts.net	Data catalog	EU	Health	FA	no	164 datasets
OccupationalExposureTools.net	Data catalog	EU	Health	FA	no	11 datasets
Open Access Infrastructure for Research in Europe (OpenAIRE)	Data platform	EU	Multidisciplinary	FA	no	74 million datasets, 10 932 repositories
Open Science Framework (OSF)	Data platform	International	Multidisciplinary	FA	yes	2 600 datasets
OpenDoar	Data platform	International	Multidisciplinary	FA	no	5 982 repositories
Our World in Data	Data repository	International	Multidisciplinary	FA	yes	
Registry of Research Data Repositories (Re3Data)	Data repository	International	Multidisciplinary	FA	no	3 331 repositories
ScholeXplorer	Data platform	International	Multidisciplinary	Sub	?	1.15 billion datasets
Scientific Data	Data-focused journal	International	Multidisciplinary	OA	yes	
TEDI (Toxicological and Exposure Database Inventory)	Data catalog	International	Public health, toxicology, environmental health	FA	no	1 055 datasets
UK data service	Governmental data portal	UK	Multidisciplinary	FA	no	9 877 datasets
World Health Organization	Data repository	International	Health	FA	yes	
Zenodo	Data repository	International	Multidisciplinary	FA	yes	403 919 datasets

Note: DL: indicates whether datasets can be directly downloaded from the discovery resource; FA: freely accessible; FR: free registration required; OA: open-access publications; RA: type of access to the dataset discovery resource; Sub: subscription required. The searchable dataset resource names are provided as hyperlinks.

4. Proposed framework for datagraphic search

The principles of systematic searching, traditionally associated with systematic reviews and meta-analyses [31], can be effectively applied to dataset discovery. This structured approach aims to identify all relevant datasets within resource constraints, enhance transparency in the search process, and ensure reproducibility. By providing a rigorous and replicable framework for dataset selection, it mitigates subjective biases and fosters collaboration across disciplines, benefiting not only researchers but also industry professionals, stakeholders, and other interested parties. Consequently, datagraphy has the potential to empower a wide range of users (e.g., researchers and policymakers) by facilitating access to diverse types of knowledge. To ensure the relevance and utility of a datagraphic search, authors should provide a transparent, comprehensive, and accurate account of the rationale behind the search, the methodology employed (including dataset identification and selection criteria), and the key findings (such as dataset characteristics).

To operationalize this process, we propose a nine-step framework that mirrors the systematic approach used in bibliographic research (Figure 1).

Step 1 involves defining the research question, a fundamental component of scientific inquiry [46]. Establishing a clear research question helps determine the specific issues that should be addressed through data analysis. Existing guidelines, such as those provided by the Joanna Briggs Institute, can aid in this process by applying structured frameworks, such as the population, concept, and context criteria (PCC) [47].

Step 2 entails specifying data requirements, including essential variables, study population, timeframes, geographic scope, granularity, and data formats [10].

Step 3 involves defining the dataset search strategy, analogous to approaches used in bibliographic literature searches. Several recommendations used for systematic reviews have been formulated and could be followed to find the best balance between sensitivity and specificity [48]. However, because relevant information is often dispersed across multiple datasets, search strategies frequently need to be adapted. For instance, administrative health databases such as the French National Health Data System (SNDS) [49] may lack key epidemiological variables, such as environmental factors (e.g., air pollution, climate data) [6]. In such cases, distinct search strategies may be required to identify both administrative

health records and complementary contextual datasets that can be integrated. In addition, some platforms, such as *DataONE*, support semantic technologies that automatically expand user-entered keywords to include relevant synonyms [10]. In contrast, if a search portal lacks this functionality, users must manually include appropriate synonyms to ensure comprehensive results [10].

Step 4 focuses on dataset discovery. Researchers can leverage various data repositories, platforms, catalogs, data papers, and other resources to identify potential eligible/relevant datasets. In many cases, discipline-specific repositories provide the most effective means of discovery, as researchers with similar interests are more likely to store and share datasets within these specialized platforms [14]. Domain-specific portals further streamline the search process by offering interfaces and filters tailored to the needs of particular research fields [10]. In addition, data aggregators such as *DataONE* and *DataMed* allow users to search multiple repositories through a single interface [10]. However, not all data discovery resources are equally trustworthy. For example, repositories certified by the *CoreTrustSeal* must meet 16 criteria related to accessibility, usability, reliability, and long-term data preservation [10,50]. Understanding the standards and practices a searchable dataset resource applies to its data and metadata can increase confidence in data quality and reusability [10]. A recent study has also outlined eleven practical tips for dataset discovery, providing a useful starting point or foundational guidance for this endeavor [10].

Step 5 evaluates whether a dataset is accessible and under what conditions. While some datasets can be downloaded directly from the web, others may require a subscription, direct contact with the authors, or approval from data custodians.

Step 6 pertains to ensuring ethical and legal compliance. This includes verifying adherence to data-sharing policies, privacy regulations, and licensing constraints to safeguard responsible data use. This step aims to check whether a dataset can be reused and how.

Step 7 involves assessing dataset relevance (eligibility) by evaluating its alignment with the research objectives, including considerations such as scope, granularity, and contextual applicability. An initial review of the metadata is often sufficient to determine whether a dataset meets the preliminary requirements defined in Steps 1 and 2 [10]. Some searchable dataset resources, such as *Figshare*, offer preview features that allow users to quickly evaluate the dataset's structure and content. Ideally,

metadata should be accompanied by comprehensive documentation to support a thorough evaluation of the dataset's relevance and fitness for use. This includes details on data collection methods, quality assurance procedures, and prior applications of the data [10]. If a dataset fails to meet any of the established criteria, it may be advisable to exclude it from further consideration [10].

Step 8 requires evaluating data quality by examining completeness, representativeness, interoperability, generalizability, validity, and potential limitations, such as missing data or measurement errors [27]. Key considerations include whether the dataset contains the necessary variables to address the research question, whether the data collection and its sampling methodology are appropriate, how variables are defined and measured, and whether the sample size is sufficient to ensure adequate statistical power. Other critical factors include the extent of missing data and, in longitudinal studies, the degree of loss to follow-up [11]. To facilitate a robust quality assessment, predefined metrics (e.g., accuracy, completeness, consistency, timeliness, currency, conformance, and uniqueness) can be applied [51]. This step also aims to assess whether data management or wrangling is required and, if so, to determine its potential scope [52]. The feasibility of data reuse may decline if the effort required to standardize a dataset (i.e., make it research-ready) for research purposes is disproportionately high relative to its potential benefits [52]. In addition, ensuring high data quality is essential for deriving meaningful insights [51]. A recent study offers guidance on evaluating dataset quality in the context of machine learning [51], which can serve as a valuable starting point for implementing Step 8 of the proposed framework.

Step 9 involves transparently documenting the dataset identification and selection process, following a structured approach similar to that of systematic reviews (e.g., PRISMA-like flowchart [31]) (Figure 2). This ensures clarity and reproducibility, ultimately strengthening the reliability of data reuse research.

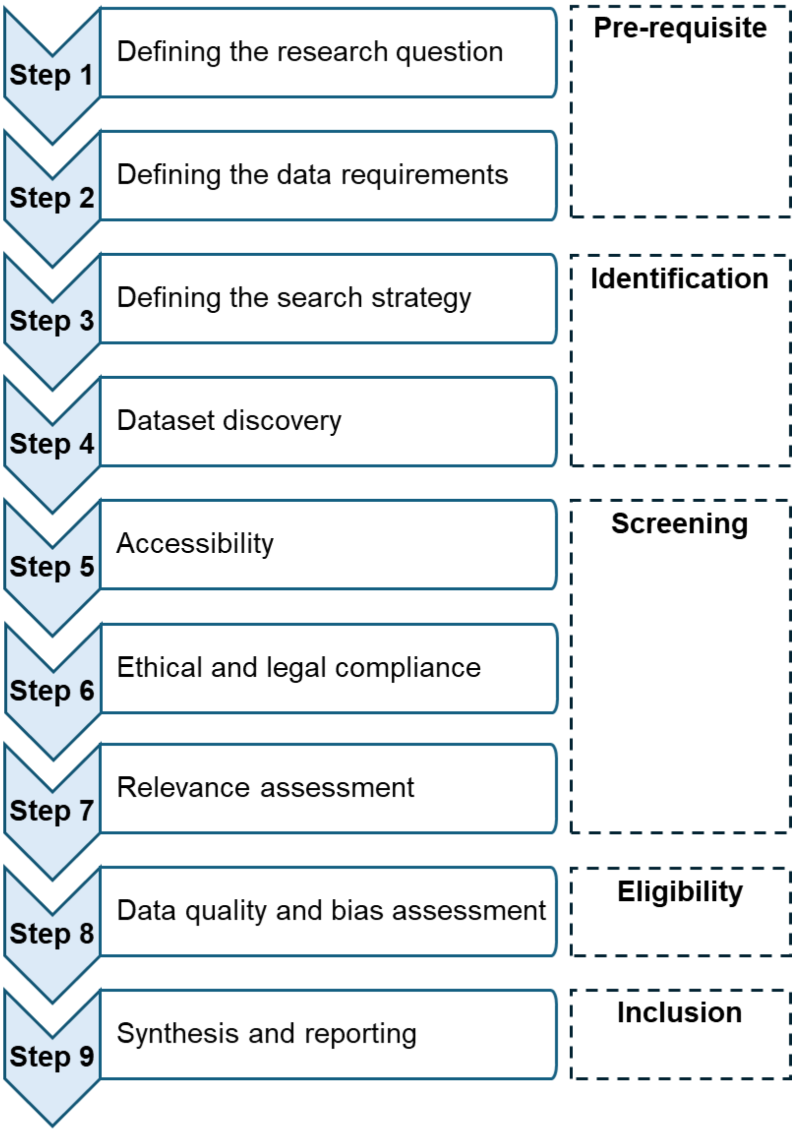

Figure 1: Proposed datagraphy framework

Operationalization of the datagraphy concept with a nine-step approach that mirrors the systematic method used in bibliographic research.

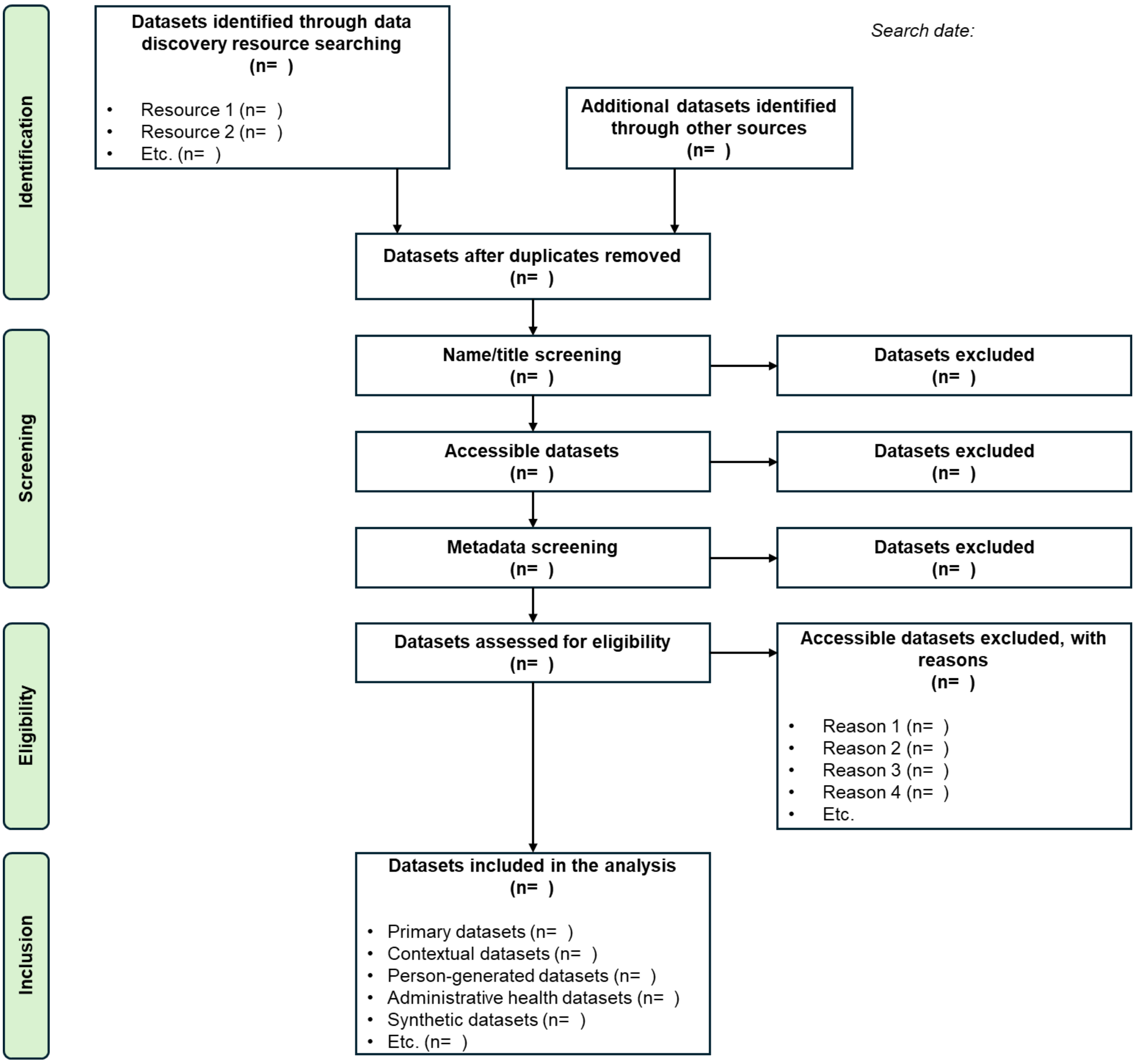

Figure 2: PRISMA-like flowchart for datagraphy

Flowchart illustrating the datagraphy concept.

5. Illustrative and working example: the exposome case

To demonstrate the application of the proposed framework, we conducted a datagraphic search focused on the exposome. The research question was formulated using the Joanna Briggs Institute's PCC framework [47]. The population of interest included any human subjects exposed to environmental factors. The concept encompassed any studies involving at least one health-related outcome. No restrictions were applied for context.

When possible, we used the same search query (i.e., exposom*) as in a prior bibliometric analysis on the exposome [7]. For searchable dataset resources that did not support wildcard characters, we used the simplified query "exposome." The search was carried out across 11 dataset discovery platforms, following a process inspired by the Preferred Reporting Items for Systematic Reviews and Meta-Analyses (PRISMA) guidelines [31] and the practical recommendations outlined by Gregory et al. [10]. For this example, screening was performed by a single author. To minimize bias due to temporal variability in dataset availability, all data were collected on the same day, April 10, 2025.

To ensure the relevance and representativeness of the included datasets, titles/names, metadata, and dataset content were assessed based on predefined inclusion and exclusion criteria (Table 1). When titles or metadata lacked sufficient information, full-content screening was conducted. Datasets were included if they addressed the human exposome. Exclusion criteria encompassed datasets involving only animal or in vitro data, those irrelevant to the exposome concept, or those lacking individual-level data on exposures, outcomes, and participant characteristics. For this illustrative example, we deliberately excluded datasets that contained adverse outcome data without environmental exposure/factor information, even though some could potentially be linked to contextual environmental datasets (e.g., via geographic data).

Table 1: Criteria used for the dataset selection

Question	Description	Answer	
		no	yes/can't tell
**Stage 1: Screening dataset name/title**			
Q_{11}	Does the name/title mention terms related to exposome?	0	1
Q_{12}	Does the name/title mention terms related to humans?	0	1
Q_{13}	Is the name/title in English or French?	0	1
$S_1 = Q_{11} \times Q_{12} \times Q_{13}$; Dataset eligible for stage 2 if $S_1 = 1$			
**Stage 2: Dataset accessibility**			
Q_{21}	Is the dataset freely accessible?	0	1
Q_{22}	Can the dataset be reused for research purposes?	0	1
$S_2 = Q_{21} \times Q_{22}$; Dataset eligible for stage 3 if $S_2 = 1$			
**Stage 3: Screening dataset metadata**			
Q_{31}	Is there a data dictionary explaining the dataset content?	0	1
Q_{32}	Does the metadata mention terms related to exposome?	0	1
Q_{33}	Does the metadata mention terms related to humans?	0	1
Q_{34}	Is there any individual data available?	0	1
Q_{35}	Is the data real (not synthetic)?	0	1
$S_3 = Q_{31} \times Q_{32} \times Q_{33} \times Q_{34} \times Q_{35}$; Dataset eligible for stage 4 if $S_3 = 1$			
**Stage 4: Screening dataset content**			
Q_{41}	Is the data in English or French?	0	1
Q_{42}	Does the dataset structure/format allow its reuse?	0	1
Q_{43}	Does the dataset contain individual data?	0	1
Q_{44}	Does the dataset contain an ID for each participant?	0	1
Q_{45}	Does the dataset contain exposure data?	0	1
Q_{46}	Does the dataset contain participants' characteristics?	0	1
Q_{47}	Does the dataset contain outcome data (e.g., presence or absence of a disease)?	0	1
$S_4 = Q_{41} \times Q_{42} \times Q_{43} \times Q_{44} \times Q_{45} \times Q_{46} \times Q_{47}$; Dataset eligible for inclusion if $S_4 = 1$			
Score = $S_1 \times S_2 \times S_3 \times S_4$; Dataset selected for review/analysis if score = 1			

Note: Q: question, S: score.

Results from the datagraphic search are presented in Figure 3, with step-by-step details provided in Table A.1 (Supplementary Materials). Of the 11 searchable dataset resources, six supported the use of the wildcard query "exposom*." A total of 322 datasets were initially retrieved. After removing 109 duplicates (34%), 213 unique records remained for screening. Duplicate identification was challenging due to variations in dataset names and the presence of subsets nested within larger datasets. In cases where subsets offered no additional unique information, only the original dataset was retained.

Title-based screening excluded 85 datasets (40%). Of the remaining 128, 22 (17%) were inaccessible, resulting in 106 datasets for metadata screening. Of these, 96 (91%) were excluded, most commonly due to the absence of individual-level data (n=67, 70%) or a lack of exposure/environmental data (n=20, 21%).

The final screening phase involved full content review of the remaining 10 datasets, from which 4 met the inclusion criteria. These included two datasets from the National Health and Nutrition Examination Survey (NHANES) [38,53], one from a Pakistani cohort study [37], and one from the EXPOsOMICS

Personal Exposure Monitoring Study [54]. Dataset quality assessment was not conducted for this illustrative example.

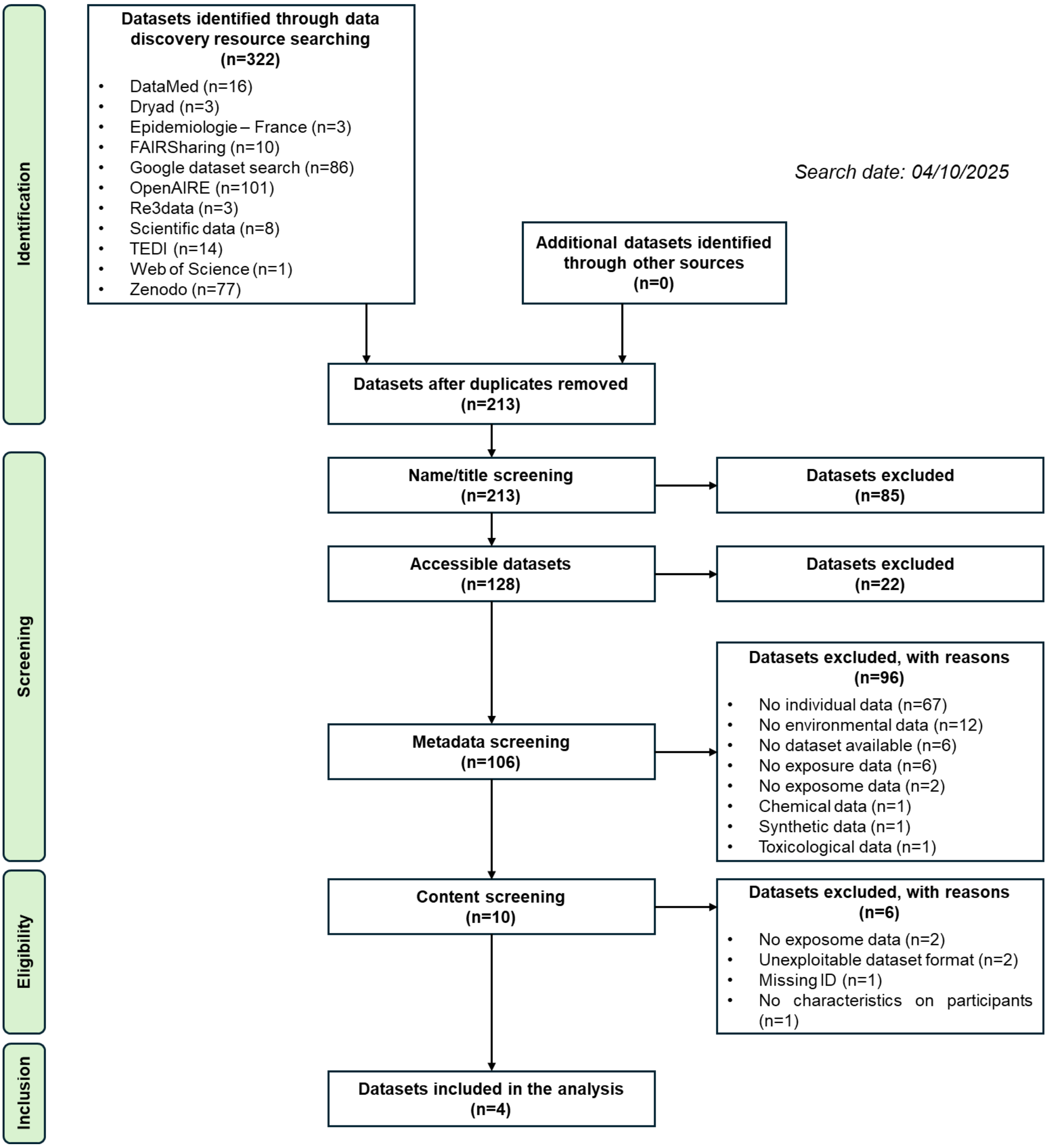

Figure 3: PRISMA-like flowchart of the exposome datagraphic search

Flowchart summarizing the results of the exposome datagraphic search.

6. Discussion

6.1 Challenges

The successful implementation of datagraphy faces several challenges. One of the primary obstacles is the availability of metadata, which is essential for assessing the existence and characteristics of datasets. Researchers frequently encounter poorly described and non-standardized data, limiting their reusability [19]. As a result, substantial time and effort are required to manually sift through large volumes of irrelevant datasets. Major heterogeneity exists among searchable dataset discovery resources in terms of data uploading, management, and access procedures [50]. Existing searchable dataset discovery resources (e.g., data repositories) accept a wide variety of data types and formats but generally do not attempt to integrate, harmonize, and check the quality of the deposited data. Deposited datasets may be incomplete at times, intentionally so [24]. In addition, existing searchable dataset discovery resources impose minimal restrictions on the descriptors used for data deposition [16]. As a result, the data ecosystem is becoming increasingly fragmented and heterogeneous, further complicating dataset discovery and leading to a "needle in a haystack" paradox [16]. This variability in metadata and the lack of standardized protocols for data collection, storage, and format/structure among repositories create major barriers to the effective identification and reuse of datasets. In the absence of consensus on core repository services, each organization tends to implement systems based on its specific goals [50], making cross-repository integration challenging. For instance, in data papers and data-focused journals, publication requirements and the amount of data that can be attached vary by journal, making it challenging to find comprehensive and useful content. Moreover, limited accessibility and machine-readability of existing metadata, in particular for population health data, hamper effective dataset discovery and reuse [55,56]. Often, available information is provided only at the most general level, highlighting deficiencies in metadata.

Beyond metadata challenges, searchable dataset discovery resources (e.g., data repositories) also differ in terms of functionality and coverage, including subject matter, geographical and temporal scope, language, and scientific domain [2,30]. Two recent reviews, covering 35 [19] and 25 [50] data repositories respectively, identified interoperability and sustainability as major obstacles to fulfilling the FAIR data principles. Consequently, the suitability of a given resource depends on its specific attributes. Existing tools allow searches for spreadsheets or published data in formats such as *CSV* or *JSON*, but

most do not support complex datasets, such as collections of tables, text, or temporal data. In addition, existing searchable data discovery resources exhibit limited data discovery capabilities and superficial query responses. Translating search strategies and user queries across searchable dataset resources with differing syntax requirements can be a complex and time-consuming task [48]. To address this issue, the adoption of standardized vocabularies (e.g., taxonomies tailored to domain-specific terminology) is critical. One promising solution is the development of web-based search systems leveraging semantic metadata, as recently proposed in the field of information science for literature search of scientific publications [57]. Sustainability is another concern, with many data-sharing platforms that lack long-term funding models, posing risks to the durability and accessibility of datasets [58].

The lack of standardized indicators or scores assessing dataset reusability (e.g., FAIRness) and quality further complicates efforts to identify the most appropriate datasets for specific research questions [2,18,55]. A major challenge in assessing data quality lies in the original data collection methods and processes (i.e., data capture), which are beyond the control of secondary users [51,59].

Dataset documentation and access conditions also vary tremendously across resources, making selection difficult for researchers. Some datasets are freely available, while others cost tens of thousands of dollars to access [27]. The time required to acquire existing datasets and obtain Institutional Review Board (IRB) approval can also vary considerably [27]. While some datasets can be directly downloaded from the web (e.g., Comparative Toxicogenomics Database [60]), others require multiple layers of permissions and security measures (e.g., SNDS [49]), and in certain cases, data must be analyzed within a dedicated data processing environment (e.g., SNDS [49]). Furthermore, national and international ethical and legal obligations can restrict data sharing, grouped analyses, and data deposition [24,61]. Regulations such as the European GDPR require organizations to implement robust data protection measures, including data retention and deletion protocols, which can impede data reuse efforts and may result in substantial financial penalties in case of non-compliance [22,62].

Certain data sources, such as administrative health records, currently lack comprehensive catalogs [6]. Additionally, some datasets remain inaccessible behind paywalls, going unnoticed by researchers [30], while others are not deposited in open repositories (referred to as invisible data or dark data [13]), increasing the risk of data graveyards (i.e., unused data) [63]. Over time, the availability of originally

accessible data tends to decline (a phenomenon known as data decay), which can ultimately lead to data loss [24]. Access to published data is not always guaranteed due to broken links, missing metadata, or a lack of willingness by authors to share data upon request [24]. For example, in one large-scale study of around 900 articles published in *Nature* and *Science* between 2000 and 2019, 61% of papers that included "data available upon request" statements did not provide the data when contacted [24]. Sharing data via supplementary materials is also common in the research community [64]. However, unlike public repositories, supplementary materials are not persistently discoverable or archived. They are not hosted as part of the journal's permanent record, lack persistent identifiers (e.g., DOIs or accession numbers), and are vulnerable to link rot and content drift [64]. In addition, supplementary material data is often not available as individual or raw data but as aggregated or summary information that cannot be easily reused.

6.7 Potential implications and future directions

Establishing datagraphy as a recognized research methodology could have profound implications across scientific disciplines. By promoting systematic dataset selection, datagraphy may enhance the reliability and reproducibility of data reuse research, mitigate selection bias, facilitate effective data reuse, and foster interdisciplinary collaboration by improving dataset discoverability. As scientific data grow in volume and complexity, the need for a structured approach to dataset selection becomes increasingly urgent. Integrating datagraphy as a foundational research practice will ensure that dataset selection adheres to the same methodological rigor as literature reviews, ultimately strengthening the integrity and impact of data-driven discoveries.

To advance datagraphy, standardization efforts are essential, as was done for bibliographic search. Future research should focus on developing standardized guidelines for datagraphic reporting, leveraging artificial intelligence (AI) for automated dataset evaluation, and fostering community-driven initiatives to refine best practices. Establishing reporting guidelines analogous to existing ones, such as PRISMA [31], could be a viable approach. Another alternative could be the development of an extension of existing frameworks (e.g., PRISMA guidelines for dataset discovery).

The adoption of persistent identifiers (e.g., digital object identifiers, or DOIs) for datasets is essential for datagraphy. However, this practice is currently underdeveloped [12,18]. Assigning persistent identifiers ensures long-term accessibility, facilitates dataset citation, and prevents broken links due to website migrations [65]. Sustainable and trustworthy FAIR searchable data discovery resources (e.g., data repositories) must be developed to support these efforts.

The creation of a comprehensive search index could enable dataset comparison [42]. However, empirical insights into what makes a dataset more reusable remain limited [2]. Guidelines such as FAIR, which promote universal metadata standards, are essential for dataset comparison and integration [18,55]. While measuring "*FAIRness*" is not yet an established practice, several efforts (e.g., the *FAIR metrics* group) are paving the way [2]. Implementing FAIR principles enhances discoverability and reuse, ensuring seamless dataset discovery, access, and integration across diverse research domains [55,56,65]. Complementing the FAIR principles with the TRUST (Transparency, Responsibility, User focus, Sustainability, and Technology) principles could provide a more comprehensive approach to data sustainability [58]. Additional initiatives, such as *Make Data Count*, aim to establish data as a recognized research output [18].

Integrating dataset search tools with literature databases and data management platforms is another critical step [29]. For instance, *Web of Science* allows filtering for data papers, but not other bibliometric databases, such as *PubMed*. Developing unified guidelines for cloud architectures [66], promoting common data elements [67,68], and standardizing reporting formats and metadata sharing are also essential [69]. Richly described metadata in machine-readable formats will enhance interoperability, enabling efficient data harvesting, attribution, and content understanding [56,65,67-69]. Ongoing harmonization efforts, such as those in toxicology [61], should be further encouraged.

Initiatives such as *DCAT* [2], *DATACC* (https://www.datacc.org/en/), and *schema.org* [2] can enhance datagraphy by increasing dataset discoverability and enabling federated searches across multiple data catalogs. A unified data service is needed to efficiently retrieve relevant and reliable datasets [42]. The European initiative *DataGEMS* [70] aims to address this challenge by developing an advanced data discovery platform based on FAIR principles. *DataGEMS* will integrate data sharing, discovery, and analysis into a comprehensive ecosystem covering the entire data lifecycle (i.e., storage, management,

discovery, analysis, and reuse). This EU-funded initiative involves twelve partners across eight countries working to create open-source tools that facilitate access to FAIR-by-design datasets. Leveraging state-of-the-art data management, natural language processing, and machine learning, *DataGEMS* will support dataset discovery and analysis across diverse data modalities, including tabular data, text documents, knowledge graphs, and images. By promoting data FAIRness, *DataGEMS* will bridge the gap between data providers and users, fostering a more efficient data-sharing ecosystem.

The integration of AI-based tools for automated dataset discovery could represent a promising avenue for enhancing datagraphy. Ultimately, establishing datagraphy as a prerequisite for new studies and data collection could meaningfully improve the quality, transparency, sustainability, and impact of scientific research by empowering individuals (e.g., researchers, stakeholders, and policymakers) with valuable insights.

List of abbreviations

AI: artificial intelligence

DGA: Data Governance Act

DOI: digital identifier of an object

EU: European Union

FAIR: Findable, Accessible, Interoperable, and Reusable

GDPR: General Data Protection Regulation

IRB: Institutional Review Board

NHANES: National Health and Nutrition Examination Survey

PCC: population, concept, and context criteria

PRISMA: Preferred Reporting Items for Systematic Reviews and Meta-Analyses

RDA: Research Data Alliance

SNDS: French National Health Data System

TRUST: Transparency, Responsibility, User focus, Sustainability, and Technology

WHO: World Health Organization

Acknowledgments

None.

Declaration of interests

The authors declare that they have no known competing financial interests or personal relationships that could have appeared to influence the work reported in this paper.

Funding

This work was partially supported by funding from the French government, managed by the National Research Agency (ANR) under the France 2030 program [ANR-23-IACL-0006] and under the "Investissements d'avenir" program [ANR-10-AIRT-05 and ANR-15-IDEX-02]. The funders played no role in study design, data collection, analysis and interpretation of data, or the writing of this manuscript.

Author's contribution

Pascal Petit: Conceptualization, Methodology, Software, Validation, Investigation, Data Curation, Writing - Original Draft, Writing - Review & Editing, Visualization, Supervision, Project administration, Funding acquisition.

Nicolas Vuillerme: Methodology, Validation, Writing - Review & Editing, Supervision, Project administration, Funding acquisition, Resources.

Data availability statement

The authors confirm that the data supporting the findings of this study are available within the article and its supplementary materials.

References

1. Pastorino R, De Vito C, Migliara G, et al. Benefits and challenges of Big Data in healthcare: an overview of the European initiatives. Eur J Public Health. 2019;29(Supplement_3):23-27. https://doi.org/10.1093/eurpub/ckz168.
2. Koesten L, Vougiouklis P, Simperl E, Groth P. Dataset Reuse: Toward Translating Principles to Practice. Patterns (N Y). 2020;1(8):100136. https://doi.org/10.1016/j.patter.2020.100136.
3. Peterson ED. Machine Learning, Predictive Analytics, and Clinical Practice: Can the Past Inform the Present?. JAMA. 2019;322(23):2283-2284. https://doi.org/10.1001/jama.2019.17831.
4. Li C, Huang M. Environmental Sustainability in the Age of Big Data: Opportunities and Challenges for Business and Industry. Environ Sci Pollut Res Int. 2023;30(56):119001-119015. https://doi.org/10.1007/s11356-023-30301-5.
5. Näher AF, Vorisek CN, Klopfenstein SAI, et al. Secondary data for global health digitalisation. Lancet Digit Health. 2023;5(2):e93-e101. The Lancet. Digital health, 5(2), e93–e101. https://doi.org/10.1016/S2589-7500(22)00195-9.
6. Petit P, Vuillerme N. Leveraging Administrative Health Databases to Address Health Challenges in Farming Populations: Scoping Review and Bibliometric Analysis (1975-2024). JMIR Public Health Surveill. 2025;11:e62939. https://doi.org/10.2196/62939.
7. Petit P, Vuillerme N. Global research trends on the human exposome: a bibliometric analysis (2005-2024). Environ Sci Pollut Res Int. 2025;32(13):7808-7833. https://doi.org/10.1007/s11356-025-36197-7.
8. Tenopir C, Rice NM, Allard S, et al. Data sharing, management, use, and reuse: Practices and perceptions of scientists worldwide. PLoS One. 2020;15(3):e0229003.
9. Chabilall J, Brown Q, Cengiz N, Moodley K. Data as scientific currency: Challenges experienced by researchers with sharing health data in sub-Saharan Africa. PLOS Digit Health. 2024;3(10):e0000635. https://doi.org/10.1371/journal.pdig.0000635.

10. Gregory K, Khalsa SJ, Michener WK, Psomopoulos FE, de Waard A, Wu M. Eleven quick tips for finding research data. PLoS Comput Biol. 2018;14(4):e1006038. https://doi.org/10.1371/journal.pcbi.1006038.
11. Doolan DM, Winters J, Nouredini S. Answering Research Questions Using an Existing Data Set. Med Res Arch. 2017;5(9).
12. Pierce HH, Dev A, Statham E, Bierer BE. Credit data generators for data reuse. Nature. 2019;570(7759):30-32. https://doi.org/10.1038/d41586-019-01715-4.
13. Yee M, Surkis A, Lamb I, Contaxis N. The NYU Data Catalog: a modular, flexible infrastructure for data discovery. J Am Med Inform Assoc. 2023;30(10):1693-1700. https://doi.org/10.1093/jamia/ocad125.
14. Contaxis N, Clark J, Dellureficio A, et al. Ten simple rules for improving research data discovery. PLoS Comput Biol. 2022;18(2):e1009768. https://doi.org/10.1371/journal.pcbi.1009768.
15. Vesteghem C, Brøndum RF, Sønderkær M, et al. Implementing the FAIR Data Principles in precision oncology: review of supporting initiatives. Brief Bioinform. 2020;21(3):936-945. https://doi.org/10.1093/bib/bbz044.
16. Wilkinson MD, Dumontier M, Aalbersberg IJ, et al. The FAIR Guiding Principles for scientific data management and stewardship. Sci Data. 2016;3:160018. https://doi.org/10.1038/sdata.2016.18.
17. Dul J, van Raaij E, Caputo A. Advancing scientific inquiry through data reuse: Necessary condition analysis with archival data. Strateg Chang. 2024;33:35–40. https://doi.org/10.1002/jsc.2562.
18. Sielemann K, Hafner A, Pucker B. The reuse of public datasets in the life sciences: potential risks and rewards. PeerJ. 2020;8:e9954. https://doi.org/10.7717/peerj.9954.
19. Guillot P, Bøgsted M, Vesteghem C. FAIR sharing of health data: a systematic review of applicable solutions. Health Technol. 2023;13:869–882. https://doi.org/10.1007/s12553-023-00789-5.

20. Lalova-Spinks T, Meszaros J, Huys I. The application of data altruism in clinical research through empirical and legal analysis lenses. Front Med (Lausanne). 2023;10:1141685. https://doi.org/10.3389/fmed.2023.1141685.
21. Shabani M. The Data Governance Act and the EU's move towards facilitating data sharing. Mol Syst Biol. 2021;17(3):e10229. https://doi.org/10.15252/msb.202110229.
22. Vlahou A, Hallinan D, Apweiler R, et al. Data Sharing Under the General Data Protection Regulation: Time to Harmonize Law and Research Ethics?. Hypertension. 2021;77(4):1029-1035. https://doi.org/10.1161/HYPERTENSIONAHA.120.16340.
23. Raab R, Küderle A, Zakreuskaya A, et al. Federated electronic health records for the European Health Data Space. Lancet Digit Health. 2023;5(11):e840-e847. https://doi.org/10.1016/S2589-7500(23)00156-5.
24. Tedersoo L, Küngas R, Oras E, et al. Data sharing practices and data availability upon request differ across scientific disciplines. Sci Data. 2021;8(1):192. https://doi.org/10.1038/s41597-021-00981-0.
25. Aleixandre-Benavent R, Vidal-Infer A, Alonso-Arroyo A, Peset F, Ferrer Sapena A. Research Data Sharing in Spain: Exploring Determinants, Practices, and Perceptions. Data. 2020;5(2):29. https://doi.org/10.3390/data5020029.
26. World Bank. Open data for sustainable development. Policy Note ICT01. August 2015. 2015. Retrieved from https://thedocs.worldbank.org/en/doc/741081441230716917-0190022015/original/OpenDataforSustainabledevelopmentPNFINALONLINESeptember1.pdf. Accessed April, 10, 2025.
27. Smith AK, Ayanian JZ, Covinsky KE, et al. Conducting high-value secondary dataset analysis: an introductory guide and resources. J Gen Intern Med. 2011;26(8):920-929. https://doi.org/10.1007/s11606-010-1621-5.
28. Trifan A, Oliveira JL. Patient data discovery platforms as enablers of biomedical and translational research: A systematic review. J Biomed Inform. 2019;93:103154. https://doi.org/10.1016/j.jbi.2019.103154.

29. Gregory K. A dataset describing data discovery and reuse practices in research. Sci Data. 2020;7(1):232. https://doi.org/10.1038/s41597-020-0569-5.
30. Gusenbauer M. Search where you will find most: Comparing the disciplinary coverage of 56 bibliographic databases. Scientometrics. 2022;127(5):2683-2745. https://doi.org/10.1007/s11192-022-04289-7.
31. Page MJ, McKenzie JE, Bossuyt PM, et al. The PRISMA 2020 statement: an updated guideline for reporting systematic reviews. BMJ. 2021;372:n71. https://doi.org/10.1136/bmj.n71.
32. Schmitt CP, Stingone JA, Rajasekar A, et al. A roadmap to advance exposomics through federation of data. Exposome. 2023;3(1):osad010. https://doi.org/10.1093/exposome/osad010.
33. Gregory K, Groth P, Scharnhorst A, Wyatt S. Lost or found? Discovering data needed for research. Harvard Data Sci Rev. 2020;2. https://doi.org/10.1162/99608f92.e38165eb.
34. Wickham RJ. Secondary Analysis Research. J Adv Pract Oncol. 2019;10(4):395-400. https://doi.org/10.6004/jadpro.2019.10.4.7.
35. Kogevinas M, Schlünssen V, Mehlum IS, Turner MC. The OMEGA-NET International Inventory of Occupational Cohorts. Ann Work Expo Health. 2020;64(6):565-568. https://doi.org/10.1093/annweh/wxaa039.
36. Peters S. Although a valuable method in occupational epidemiology, job-exposure -matrices are no magic fix. Scand J Work Environ Health. 2020;46(3):231-234. https://doi.org/10.5271/sjweh.3894.
37. Gul F, Herrema H, Davids M, et al. Gut microbial ecology and exposome of a healthy Pakistani cohort. Gut Pathog. 2024;16(1):5. https://doi.org/10.1186/s13099-024-00596-x.
38. Patel CJ, Pho N, McDuffie M, et al. A database of human exposomes and phenomes from the US National Health and Nutrition Examination Survey. Sci Data. 2016;3:160096. https://doi.org/10.1038/sdata.2016.96.
39. Dimitrova M, Meyer R, Buttigieg PL, et al. A streamlined workflow for conversion, peer review, and publication of genomics metadata as omics data papers. Gigascience. 2021;10(5):giab034. https://doi.org/10.1093/gigascience/giab034.

40. Suhr B, Dungl J, Stocker A. Search, reuse and sharing of research data in materials science and engineering-A qualitative interview study. PLoS One. 2020;15(9):e0239216. https://doi.org/10.1371/journal.pone.0239216.
41. Peng G, Gross WS, Edmunds R. Crosswalks among stewardship maturity assessment approaches promoting trustworthy FAIR data and repositories. Sci Data. 2022;9(1):576. https://doi.org/10.1038/s41597-022-01683-x.
42. Krämer T, Klas CP, Hausstein B. A data discovery index for the social sciences. Sci Data. 2018;5:180064. https://doi.org/10.1038/sdata.2018.64.
43. Pampel H, Weisweiler NL, Strecker D, et al. re3data - Indexing the Global Research Data Repository Landscape Since 2012. Sci Data. 2023;10(1):571. https://doi.org/10.1038/s41597-023-02462-y.
44. Doolan DM, Froelicher ES. Using an existing data set to answer new research questions: a methodological review. Res Theory Nurs Pract. 2009;23(3):203-215. https://doi.org/10.1891/1541-6577.23.3.203.
45. Petit P. Toxicological and Exposure Database Inventory: A review. Int J Hyg Environ Health. 2022;246:114055. https://doi.org/10.1016/j.ijheh.2022.114055.
46. Barroga E, Matanguihan GJ. A Practical Guide to Writing Quantitative and Qualitative Research Questions and Hypotheses in Scholarly Articles. J Korean Med Sci. 2022;37(16):e121. https://doi.org/10.3346/jkms.2022.37.e121.
47. Joanna Briggs Institute. Joanna Briggs Institute Reviewers' manual 2015: methodology for JBI scoping reviews. 2015. Retrieved from https://reben.com.br/revista/wp-content/uploads/2020/10/Scoping.pdf Accessed April 3, 2025.
48. Bramer WM, de Jonge GB, Rethlefsen ML, Mast F, Kleijnen J. A systematic approach to searching: an efficient and complete method to develop literature searches. J Med Libr Assoc. 2018;106(4):531-541. https://doi.org/10.5195/jmla.2018.283.
49. Maillard O, Bun R, Laanani M, et al. Use of the French National Health Data System (SNDS) in pharmacoepidemiology: A systematic review in its maturation phase. Therapie. 2024;79(6):659-669. https://doi.org/10.1016/j.therap.2024.05.003.

50. Banzi R, Canham S, Kuchinke W, Krleza-Jeric K, Demotes-Mainard J, Ohmann C. Evaluation of repositories for sharing individual-participant data from clinical studies. Trials. 2019;20(1):169. https://doi.org/10.1186/s13063-019-3253-3.
51. Gong Y, Liu G, Xue Y, Li R, Meng L. A survey on dataset quality in machine learning. Inf Softw Technol. 2023;162:107268. https://doi.org/10.1016/j.infsof.2023.107268.
52. Grath-Lone LM, Jay MA, Blackburn R, et al. What makes administrative data "research-ready"? A systematic review and thematic analysis of published literature. Int J Popul Data Sci. 2022;7(1):1718. https://doi.org/10.23889/ijpds.v6i1.1718.
53. Nguyen V, Middleton LYM, Zhao N, et al. Cleaned NHANES 1988-2018 [dataset]. Figshare. 2025;v9. https://doi.org/10.6084/m9.figshare.21743372.v9.
54. Oosterwegel MJ, Ibi D, Portengen L, et al. Processed metabolomic data from the EXPOsOMICS Personal Exposure Monitoring study [dataset]. Environ Sci Technol. 2023;57(34). https://doi.org/10.1021/acs.est.3c03233.
55. Alvarez-Romero C, Bernabeu-Wittel M, Luis Parra-Calderón C, Rodríguez Mejías S, Martínez-García A. Desiderata for discoverability and FAIR adoption of health data hubs. J Biomed Inform. 2024;157:104700. https://doi.org/10.1016/j.jbi.2024.104700.
56. Amadi D, Kiwuwa-Muyingo S, Bhattacharjee T, et al. Making Metadata Machine-Readable as the First Step to Providing Findable, Accessible, Interoperable, and Reusable Population Health Data: Framework Development and Implementation Study. Online J Public Health Inform. 2024;16:e56237. https://doi.org/10.2196/56237.
57. Huettemann S, Mueller RM, Dinter B. Designing ontology-based search systems for research articles. Int J Inf Manage. 2025;83:102901. https://doi.org/10.1016/j.ijinfomgt.2025.102901.
58. Kilgus T, Nowak A, Gersch M, Fürstenau D. Sustainability in Secondary Use of Health Data - A Scoping Review. Stud Health Technol Inform. 2024;316:398-402. https://doi.org/10.3233/SHTI240431.
59. Christen P, Schnell R. Thirty-three myths and misconceptions about population data: from data capture and processing to linkage. Int J Popul Data Sci. 2023;8(1):2115. https://doi.org/10.23889/ijpds.v8i1.2115.

60. Davis AP, Wiegers TC, Johnson RJ, Sciaky D, Wiegers J, Mattingly CJ. Comparative Toxicogenomics Database (CTD): update 2023. Nucleic Acids Res. 2023;51(D1):D1257-D1262. https://doi.org/10.1093/nar/gkac833.
61. Zare Jeddi M, Galea KS, Viegas S, et al. FAIR environmental and health registry (FAIREHR)-supporting the science to policy interface and life science research, development and innovation. Front Toxicol. 2023;5:1116707. https://doi.org/10.3389/ftox.2023.1116707.
62. Staunton C, Slokenberga S, Mascalzoni D. The GDPR and the research exemption: considerations on the necessary safeguards for research biobanks. Eur J Hum Genet. 2019;27(8):1159-1167. https://doi.org/10.1038/s41431-019-0386-5.
63. Custer S, Sethi T. Avoiding Data Graveyards: Insights from Data Producers & Users in Three Countries. Williamsburg, VA: AidData at William & Mary. 2017. Retrieved from https://developmentgateway.org/wp-content/uploads/2020/10/Avoiding-Data-Graveyards-Final-Report.pdf. Accessed April 10, 2025.
64. Anderson NR, Tarczy-Hornoch P, Bumgarner RE. On the persistence of supplementary resources in biomedical publications. BMC Bioinformatics. 2006;7:260. https://doi.org/10.1186/1471-2105-7-260.
65. Bayer JM, Scully RA, Dlabola EK, et al. Sharing FAIR monitoring program data improves discoverability and reuse. Environ Monit Assess. 2023;195(10):1141. https://doi.org/10.1007/s10661-023-11788-4.
66. Holub P, Kohlmayer F, Prasser F, et al. Enhancing Reuse of Data and Biological Material in Medical Research: From FAIR to FAIR-Health. Biopreserv Biobank. 2018;16(2):97-105. https://doi.org/10.1089/bio.2017.0110.
67. Basu A, Warzel D, Eftekhari A, et al. Call for Data Standardization: Lessons Learned and Recommendations in an Imaging Study. JCO Clin Cancer Inform. 2019;3:1-11. https://doi.org/10.1200/CCI.19.00056.
68. Pan H, Bakalov V, Cox L, et al. Identifying Datasets for Cross-Study Analysis in dbGaP using PhenX. Sci Data. 2022;9(1):532. https://doi.org/10.1038/s41597-022-01660-4.

69. Habermann T. Metadata and Reuse: Antidotes to Information Entropy. Patterns (N Y). 2020;1(1):100004. https://doi.org/10.1016/j.patter.2020.100004.

70. DataGEMS. Data Discovery Platform with Generalized Exploratory, Management, and Search Capabilities. 2025. Retrieved from https://doi.org/10.3030/101188416. Accessed April 10, 2025.

Figure 1

Click here to access/download;Figure;Figure_1.pdf

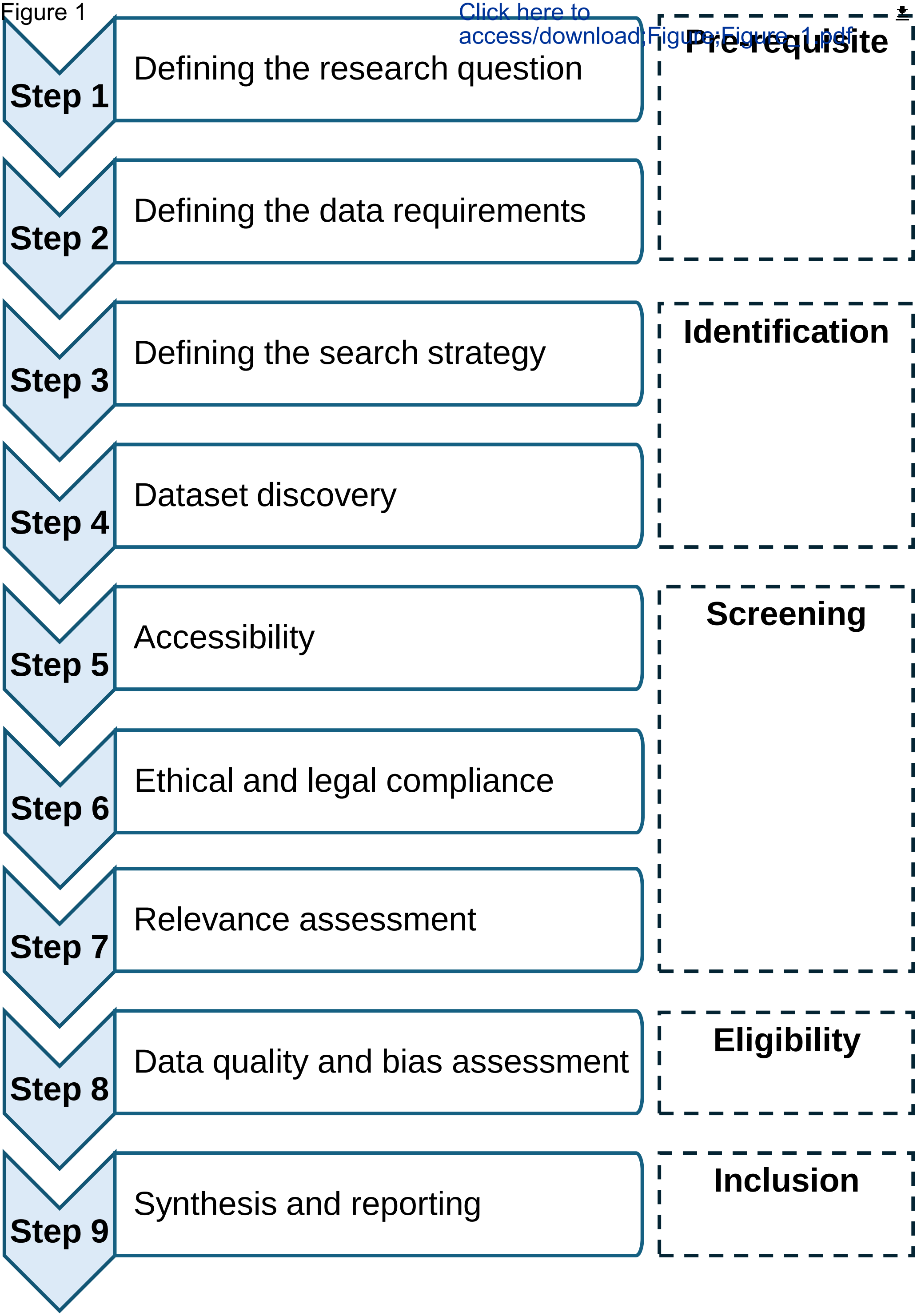

Figure 3

Click here to access/download;Figure;Figure_3.pdf

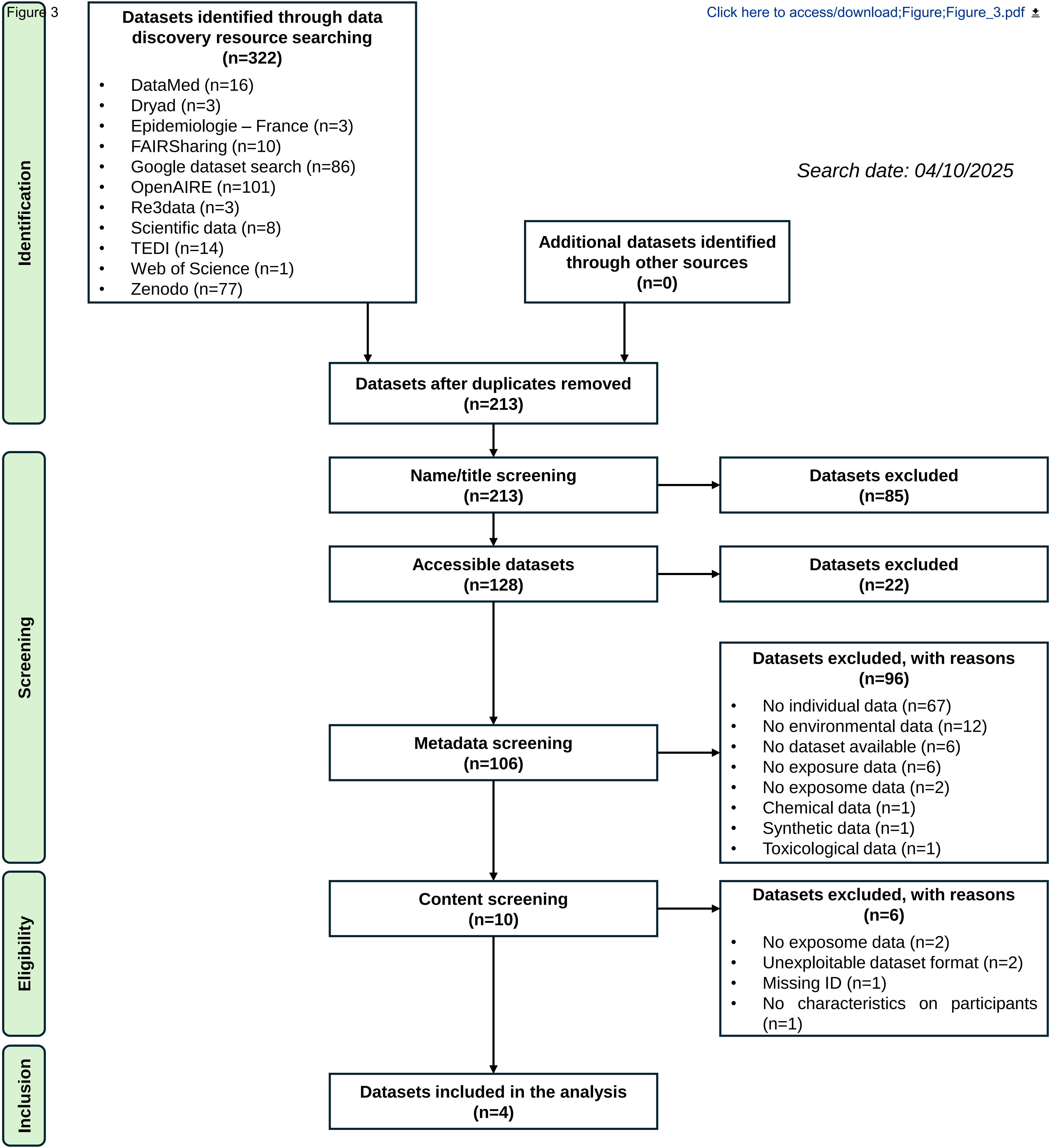

Figure 2

Click here to access/download;Figure;Figure_2.pdf

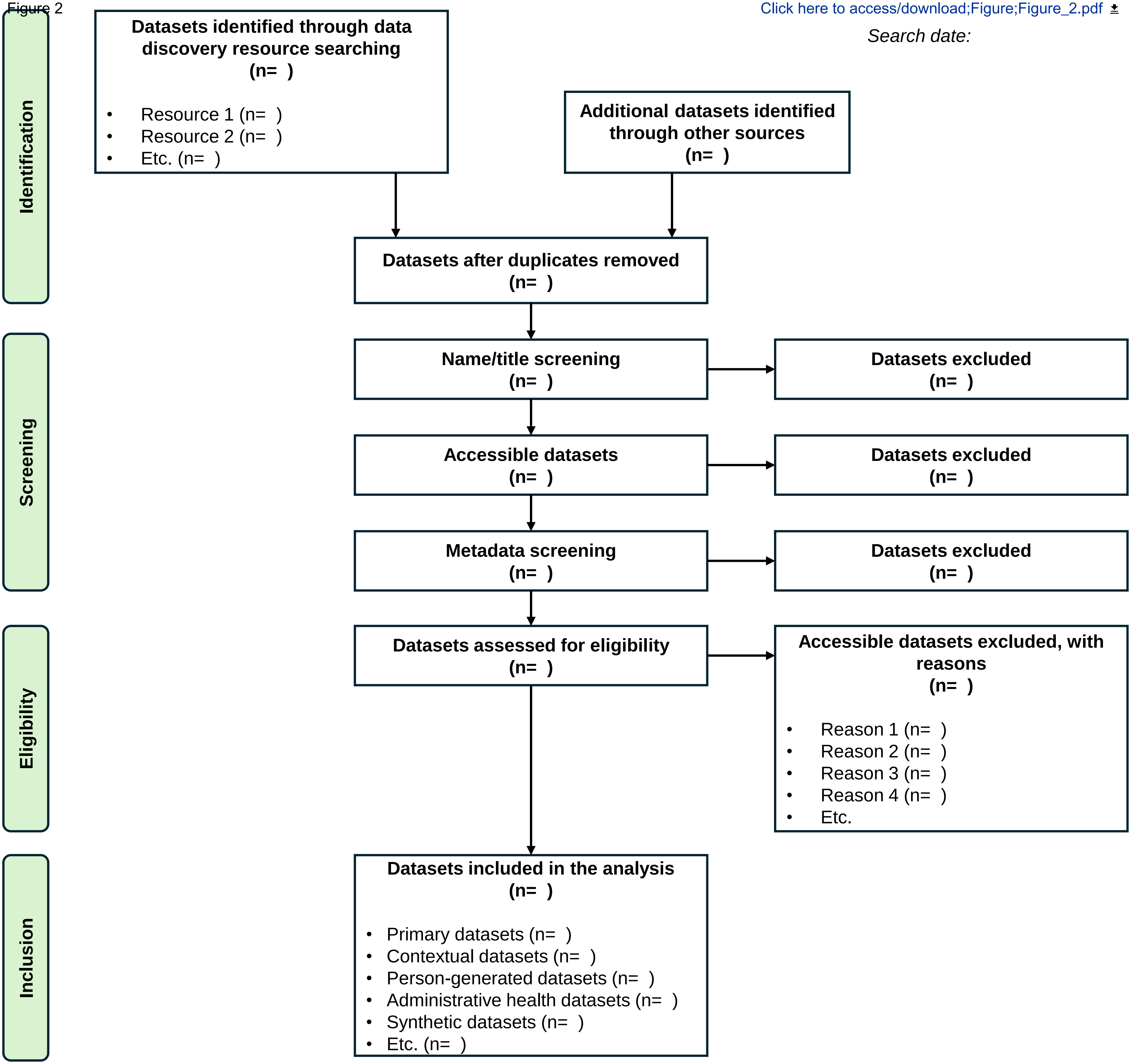

Click here to access/download
Supplementary Material
Table A.1.xlsx

Graphical Abstract

Datagraphy: toward a systematic approach to dataset discovery

Click here to access/download;Graphical

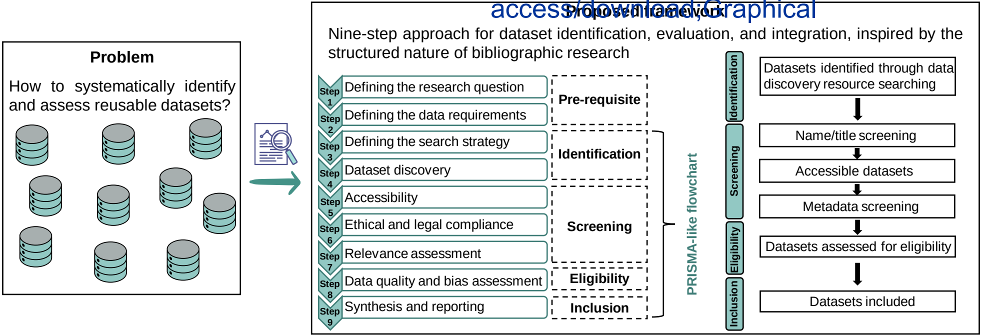

Supplement: giaf134_GIGA-D-25-00204_Original_Submission [file giaf134_giga-d-25-00204_original_submission.pdf]
