# Supplementary material for: Datagraphy: toward a systematic approach to dataset discovery
Source: Gigascience. 2025 Oct 22;14:giaf134. doi: 10.1093/gigascience/giaf134 (PMC12644982; doi:10.1093/gigascience/giaf134)

GigaScience

Datagraphy: toward a systematic approach to dataset discovery

--Manuscript Draft--

**Manuscript Number:**	GIGA-D-25-00204R1
**Full Title:**	Datagraphy: toward a systematic approach to dataset discovery
**Article Type:**	Review
**Funding Information:**	Agence Nationale de la Recherche (ANR-23-IACL-0006) — Not applicable<br>Agence Nationale de la Recherche (ANR-10-AIRT-0005) — Not applicable<br>Agence Nationale de la Recherche (ANR-15-IDEX-0002) — Not applicable
**Abstract:**	Data has become central to scientific discovery. While primary data collection remains vital, there is growing recognition of the benefits of reusing existing datasets. However, identifying suitable datasets for specific research questions is increasingly difficult due to the fragmentation and heterogeneity of the big data ecosystem. Despite the expansion of data sharing, efficient dataset discovery remains elusive, with limited empirical research on how datasets are identified, interpreted, and reused. Current dataset search practices often lack standardization, leading researchers to rely on convenience rather than systematic criteria. Unlike bibliographic research, dataset selection lacks a formal methodology, increasing the risks of bias, inefficiencies, and reduced generalizability. To address this gap, we introduce datagraphy, a structured approach to dataset identification and evaluation. Analogous to bibliographic methods but designed for datasets, datagraphy encompasses not only discovery but also critical assessment of dataset quality, relevance, interoperability, completeness, sustainability, and ethical use. By formalizing dataset search as a research practice, datagraphy seeks to improve transparency, reproducibility, and interdisciplinary collaboration, while also reducing research redundancy and environmental impact. We present a nine-step framework to operationalize datagraphy and explore challenges such as inconsistent metadata and variability among dataset discovery tools. This framework provides a foundation for systematically and reproducibly identifying and synthesizing reusable datasets. To demonstrate the application of the proposed framework, we conducted a datagraphic search focused on the exposome. We discuss major challenges faced by datagraphy with respect to metadata availability, repository heterogeneity, dataset accessibility, and dataset quality, and highlight how datagraphy could enhance transparency, reproducibility, and efficiency at the researcher level. Datagraphy is intended to complement repository-level improvements. Aligning researcher practices with standardized, machine-readable metadata, persistent identifiers, artificial intelligence integration, and lightweight packaging frameworks such as RO-Crates and FAIR Digital Objects could enable automated discovery and sustainable dataset reuse. By integrating structured researcher-level methodology with systemic improvements and community efforts, datagraphy could offer a scalable approach for systematic, FAIR-aligned data-driven research across disciplines.
**Corresponding Author:**	Pascal Petit, Ph.D.<br>Universite Grenoble Alpes<br>La Tronche Cedex, FRANCE
**Corresponding Author Secondary Information:**	
**Corresponding Author's Institution:**	Universite Grenoble Alpes
**Corresponding Author's Secondary Institution:**	
**First Author:**	Pascal Petit, Ph.D.
**First Author Secondary Information:**	
**Order of Authors:**	Pascal Petit, Ph.D.

Powered by Editorial Manager® and ProduXion Manager® from Aries Systems Corporation

	Nicolas Vuillerme, PhD, HDR
**Order of Authors Secondary Information:**	
**Response to Reviewers:**	Rebuttal letter<br><br>Dear Editor,<br><br>Please find attached the revised version of the manuscript number GIGA-D-25-00204 entitled "Datagraphy: toward a systematic approach to dataset discovery," which we are re-submitting for publication as an article in GigaScience.<br><br>We would like to warmly thank the reviewers for their constructive comments and suggestions that have been carefully taken into account in reworking this new version of the manuscript. The responses to reviewer questions and all changes made in the manuscript are listed below. A marked-up version of the manuscript with the changes to the text in red, blue, and/or green fonts was made.<br><br>Now, we hope that this revised version will be suitable for publication. In all cases, we remain open to any suggestions.<br><br>Thank you for considering our work.<br><br>Sincerely,<br>Pascal Petit, on behalf of all authors<br>Point by point response to reviewers<br><br>--<br>Reviewer #1<br>Dear Authors,<br><br>The concept that was proposed in this manuscript, datagraphy, makes it clear that there are real problems with the way current datasets are published through "generic" data repositories. The proposed framework shows a real and growing problem regarding inefficient and inconsistent dataset discovery. This problem however is in this approach tackled or perhaps bandaged by proposing a structured method toward the researcher. Although the nine steps mentioned, it remains an intensive exercise and when in a popular field would need to be done frequently to enable the discovery of new datasets.<br><br>This proposed framework however does not propose any approach to tackle the real problems. Which is the freedom data repositories have to implement their own procedures and methods for data discovery. Without this focus this sounds more like a bandaid than a solution towards the problem that is being tried to address here. In addition researchers that search for data often also deposit datasets. It might be a more, in the long term, solution if standards are proposed that aid in the discovery of datasets through standardised metadata templates that are submitted alongside the datasets such as in the form of Research Objects or RO-crates as they are essentially a lightweight approach to packaging research data with their metadata.<br><br>Overall I think the approach has merit but it does not tackle the real issue at hand.<br><br>Authors: Thank you for your review and your relevant comments. We value your time and effort.<br><br>We thank you for this valuable comment, which highlights an important limitation of our current framework and points toward relevant future directions. We fully agree that the challenges of dataset discovery cannot be resolved solely at the level of individual researchers. As you rightly note, the main root cause lies in the freedom that repositories have in implementing their own procedures, standards, and metadata requirements.<br>Our proposed framework for datagraphy, however, is not intended to replace systemic solutions but rather to provide researchers with a structured and transparent methodology for navigating the current heterogeneous ecosystem. In this sense, datagraphy can be seen as a pragmatic approach designed to improve reproducibility

and rigor under the existing conditions, while broader systemic changes are still under development.
We also agree that repository-level interventions, such as the adoption of standardized metadata templates, are indispensable to long-term progress. Your suggestion to consider Research Objects (ROs) and RO-Crates is thus particularly relevant. These initiatives provide a lightweight but powerful mechanism for packaging data together with machine-readable metadata, thereby enhancing discoverability and reusability.
We are grateful for this comment, which has helped us refine the positioning of our contribution. Our revision will therefore make clearer that while datagraphy improves transparency and reproducibility in the short term, it must evolve in tandem with repository-level initiatives that standardize metadata and enhance the FAIRness of data resources.

We have revised the manuscript to explicitly acknowledge the importance of such systemic solutions and to position datagraphy as complementary to ongoing standardization efforts. In particular, we have clarified in the discussion subsection that datagraphy is currently a "researcher-centric" framework designed to work within present limitations, while systemic and community-driven efforts are needed to address the root problem. We have also highlighted that datagraphy and community efforts should be pursued together. Datagraphy can provide the methodology for systematic dataset identification and evaluation, whereas systemic solutions (e.g., metadata standardization) will improve the underlying infrastructure for discovery.
Please refer to lines 33-43 in the revised version of the manuscript with track changes (or to lines 33-41 in the revised version without track changes): "We discuss major challenges faced by datagraphy with respect to metadata availability, repository heterogeneity, dataset accessibility, and dataset quality, and highlight how datagraphy could enhance transparency, reproducibility, and efficiency at the researcher level. Datagraphy is intended to complement repository-level improvements. Aligning researcher practices with standardized, machine-readable metadata, persistent identifiers, artificial intelligence integration, and lightweight packaging frameworks such as RO-Crates and FAIR Digital Objects could enable automated discovery and sustainable dataset reuse. By integrating structured researcher-level methodology with systemic improvements and community efforts, datagraphy could offer a scalable approach for systematic, FAIR-aligned, data-driven research across disciplines".
Please also refer to lines 185-190 in the revised version of the manuscript with track changes (or to lines 178-183 in the revised version without track changes): "At the same time, it must be acknowledged that this framework operates within the limitations of existing repository practices and infrastructures, which remain heterogeneous and largely unstandardized. Thus, the proposed methodology should be understood as a pragmatic, researcher-centric approach that enhances efficiency and rigor in dataset discovery, while recognizing that systemic repository-level improvements are still necessary to fully address the underlying challenges.".
Please also refer to lines 380-725 in the revised version of the manuscript with track changes (or to lines 369-652 in the revised version without track changes): "6. Discussion
6.1 Challenges
The successful implementation of datagraphy faces multiple challenges.

6.1.1 Metadata availability, heterogeneity, and quality
One of the primary obstacles is the limited availability of metadata, which is essential for assessing the existence and characteristics of datasets. Current deposition practices impose only minimal restrictions on the descriptors used [17]. The substantial variability in metadata and the lack of standardized protocols for data collection, storage, and format/structure across repositories create major barriers to the effective identification and reuse of datasets. While datagraphy could help researchers navigate this fragmented landscape more systematically, it does not resolve the underlying heterogeneity. In the absence of consensus on core repository services, each organization tends to implement systems aligned with its specific goals [64], making cross-repository integration challenging. For example, in data papers and data-focused journals, publication requirements and the amount of data that can be attached vary by journal, making it challenging to find comprehensive and useful content. In addition, limited accessibility and machine-readability of existing metadata, in particular for population health data, further hamper effective dataset discovery and reuse [76-78].
Metadata quality represents a further critical bottleneck. Because dataset search relies

heavily on metadata, it is imperative that descriptions are accurate and consistent [31]. Metadata are essential for establishing dataset existence, scope, and suitability for reuse, yet they are frequently incomplete, restricted to high-level descriptors, inconsistent, or non-standardized [20,30,31]. Metadata quality also varies across disciplines and repositories, reflecting differences in cultural practices and curation priorities [71]. Errors, inconsistencies, and incompleteness in metadata are common, sometimes diverging from the underlying dataset [30]. Consequently, relying solely on metadata constrains discoverability and hampers systematic evaluation of dataset fitness for purpose [30,31].

6.1.2 Data discovery infrastructure and landscape

One major issue for dataset discovery is the broad freedom repositories have to implement their own discovery procedures, metadata standards, and data packaging approaches [2,64]. This heterogeneity creates structural challenges that go beyond the capacity of individual researchers to address. While existing resources accept a wide variety of data types and formats, they generally do not attempt to integrate, harmonize, or assess the quality of deposited data. Datasets may be incomplete, sometimes intentionally so [25]. Complex datasets, such as collections of tables, longitudinal records, or multi-modal data, remain poorly supported [45]. Certain dataset types, including administrative health records, lack comprehensive catalogs and are underrepresented in existing data repositories [6]. Searchable dataset discovery resources, whether general repositories or domain-specific catalogs, also differ in functionality and coverage, including subject matter, geographical and temporal scope, language, and scientific domain [2,35]. Consequently, the data ecosystem is becoming increasingly fragmented and heterogeneous, creating a “needle in a haystack” paradox that further complicates dataset discovery [17], in particular in domains where data lakes may contain tens of thousands of tables [61].

Dataset search is largely keyword-based, restrictive, and iterative, often requiring multiple query reformulations to assess relevance [31,61]. Such keyword-based approaches, which rely on limited metadata, are insufficient for comprehensive dataset search and discovery [11,30,31]. They fail to capture dataset granularity, provenance, and methodological context [31,61]. Many repositories only support simple keyword-based queries, which limits users’ ability to express complex information needs [30]. Although some tools allow searches for spreadsheets or published data in formats such as CSV or JSON, most do not support complex datasets, such as collections of tables, text, or temporal data [11,31]. In addition, existing searchable data discovery resources often exhibit limited data discovery capabilities and provide only superficial query responses [11,31,61]. Translating search strategies and user queries across searchable dataset resources with differing syntax requirements can be a complex and time-consuming task [59]. To address this issue, the adoption of standardized vocabularies (e.g., taxonomies tailored to domain-specific terminology) is essential. One promising direction is the development of web-based search systems leveraging semantic metadata, as recently proposed in the field of information science for literature searches of scientific publications [79]. Current standards such as Wikidata, Schema.org, and Dublin Core provide structured vocabularies for describing digital resources in a consistent, machine-readable way, while domain-specific ontologies enable richer semantic annotation [31,32,44,65,80,81]. There is a growing need for richer, content-aware indexing and semantic search methods [11,30,61].

Several initiatives have developed dataset discovery indexes (DDIs) and search engines to mitigate repository heterogeneity and search limitations. Examples include DataMed, designed as a “PubMed for datasets” [62,63,82,83]; OmicsDI, which aggregates resources across proteomics, genomics, and metabolomics [40]; Auctus, a web crawler and search engine dedicated to datasets for data augmentation in machine learning [30]; and Google Dataset Search, which indexes a broad range of datasets published on the web [30,31]. These search engines attempt to index heterogeneous repositories by exposing standardized metadata fields. Most repositories, however, still support only simple searches over metadata [30], although some, such as Auctus, enable more complex queries incorporating spatial, temporal, and integration constraints based on dataset content [30]. While these approaches demonstrate the feasibility of federated search, they remain constrained by the heterogeneity and incompleteness of the underlying metadata [11,31]. Advanced approaches, including semantic search, artificial intelligence (AI)-assisted query reformulation [61], and content-aware indexing, offer potential improvements [11,30,61]. Nevertheless, search effectiveness continues to be limited primarily by

Powered by Editorial Manager® and ProduXion Manager® from Aries Systems Corporation

metadata heterogeneity and repository-specific practices [11,31].

6.1.3 Data accessibility, legal, and ethical considerations

Challenges also extend to repository functionality and scope. Dataset documentation and access conditions vary tremendously across resources, making selection difficult for researchers. Dataset accessibility is further complicated by licensing, ethical, and legal constraints. Licenses, data-use agreements (DUAs), and authentication requirements introduce additional barriers and shape who can access datasets and under what conditions [62]. Open-access datasets may be freely available, but many sensitive datasets require IRB approval, registered access, or multifactor authentication, adding layers of complexity and potential delays [60,62]. The time required to acquire existing datasets and obtain IRB approval can also vary considerably [28]. While some datasets can be directly downloaded from the web (e.g., Comparative Toxicogenomics Database [84]), others require multiple layers of permissions and security measures (e.g., SNDS [60]), and in certain cases, data must be analyzed within a dedicated data processing environment (e.g., SNDS [60]). These examples illustrate the wide diversity of access conditions researchers must navigate. Furthermore, national and international ethical and legal obligations can restrict data sharing, grouped analyses, and data deposition [25,85]. Regulations such as the European GDPR require organizations to implement robust data protection measures, including data retention and deletion protocols, which can impede data reuse efforts and may result in substantial financial penalties in case of non-compliance [23,86]. Finally, access to published data is not always guaranteed due to broken links, missing metadata, or a lack of author cooperation [25]. For example, in a large-scale study of around 900 articles published in Nature and Science between 2000 and 2019, 61% of papers that included “data available upon request” statements did not provide the data when contacted [25]. Broken links, missing metadata, or author unresponsiveness therefore remain major obstacles.

6.1.4 Data quality and reusability

Researchers frequently encounter poorly described and non-standardized data, which limits reusability [20]. Assessing dataset fitness is often difficult, as available metadata rarely capture essential attributes such as granularity, provenance, or methodological context [31,61]. As a result, substantial time and effort are often required to manually sift through large volumes of irrelevant datasets.

Another major challenge in assessing data quality and reusability lies in the original data collection methods and processes (i.e., data capture), which are beyond the control of secondary users and can highly vary [72,87]. In addition to variability in metadata completeness and repository practices, the lack of standardized indicators or score systems for assessing dataset reusability (e.g., FAIRness) and quality further complicates efforts to identify the most appropriate datasets for specific research questions [2,19,76]. Practical considerations such as transit costs, storage, and access proximity may also influence dataset reusability [67].

Sharing data via supplementary materials remains common practice and is highly encouraged by journals [88]. However, unlike public repositories, supplementary files are not necessarily persistently discoverable or archived. They frequently lack persistent identifiers (e.g., DOIs or accession numbers), making them vulnerable to link rot and content drift [88]. Moreover, supplementary datasets are often available only in aggregated or summary form, rather than as raw or individual-level data, which limits systematic reuse. Publishing datasets solely as linked data or supplementary materials is insufficient to support reproducibility and reuse, as such approaches fail to capture lifecycle information, provenance, versioning, and methodological context [89-91].

6.1.5 Data visibility, data decay, and sustainability

Systemic challenges such as dataset invisibility, data decay, broken links, and paywalls exacerbate discovery difficulties [25]. Some datasets remain inaccessible behind paywalls, making them invisible to many researchers [35], while others are never deposited in open repositories, resulting in so-called invisible data or dark data [14] and increasing the risk of data graveyards (i.e., unused data) [92]. Organizations frequently store vast numbers of tables and records in data lakes [61]. Over time, the availability of originally accessible data tends to decline (a phenomenon known as data decay), which can ultimately lead to data loss [25]. Estimates suggest that up to 80% of archived scientific research data are lost within 20 years [67].

Sustainability poses an additional systemic risk. Many data-sharing platforms lack

Powered by Editorial Manager® and ProduXion Manager® from Aries Systems Corporation

long-term funding models, which threatens not only the durability and accessibility of datasets but also the continued relevance of the information they host [93]. Without sustained funding and community support, data search tools risk becoming outdated or abandoned, which compromises their reliability and long-term utility for researchers. For example, DataMed has not received new additions since 2024 [62,63,82,83]. Some repositories risk becoming unsustainable “data lakes,” where uncurated, poorly described data accumulate without integration or retrieval mechanisms [35]. Without investment in long-term infrastructure, persistent identifiers, harmonized metadata standards, and robust governance structures, repository heterogeneity, metadata gaps, and fragmentation will persist [56]. In the absence of such measures, both discoverability and reuse remain severely undermined.

6.1.6 Standardized metadata frameworks and packaging

Efforts to improve data discoverability across the social sciences, biomedical domains, omics research, and public datasets emphasize the need for metadata harmonization, advanced search interfaces, and distributed resource indexing [32,40]. Implementing repository-wide FAIR-compliant practices and semantic metadata frameworks could help address existing limitations [38,39,81,94,95]. While the FAIR principles provide an overarching vision for dataset reuse, their adoption at the repository level remains highly uneven [20,64]. Two recent reviews, covering 35 [20] and 25 [64] data repositories, respectively, identified interoperability and sustainability as major obstacles to achieving FAIR compliance.

Repositories differ in the metadata standards they apply, the search interfaces they provide, and the approaches they adopt for data packaging. This diversity produces structural fragmentation that cannot be addressed at the level of individual researchers [2,64]. Most attempts to mitigate these limitations have focused on standardized, machine-readable metadata and packaging approaches. By adopting FAIR digital objects (FDOs) or research object (RO)-Crate packages, repositories could ensure that metadata, provenance, licensing, and dataset structure are consistently available, machine-actionable, and interoperable, thereby facilitating automated discovery and reuse across platforms [38,39,81,91,94-98].

ROs are structured bundles of data, methods, and metadata that capture lifecycle information, ownership, versioning, attribution, provenance, quality, and methodological context [89-91]. RO-Crate extends this concept by providing a lightweight, machine-readable packaging framework for aggregating research artifacts with their metadata and relationships, creating a multimodal scholarly knowledge graph that can help “FAIRify” and combine metadata from existing resources [91,94,97,99]. FDOs, in turn, are digital objects explicitly designed to be FAIR, embedding metadata, provenance, and other descriptive information in a machine-actionable format [38,65]. Other packaging standards, such as BDBags (Big Data Bags), provide mechanisms for content enumeration, fixity checking, and lightweight referencing without requiring centralized hosting [45,100]. The integration of canonical workflows, including pipelines based on the Common Workflow Language (CWL), could further support reproducibility and enable systematic evaluation of search queries and analyses across repositories [101,102].

Validation frameworks, reusability metrics, and machine-readable metadata derived from RO-Crates or FDOs could enable semi-automated or automated assessments of dataset fitness and quality [38,103,104]. For instance, the FAIRO framework has been proposed to measure the compliance of ROs with FAIR criteria [103], while reusability indicators and automated quality checks have been incorporated into RO and RO-Crate packaging [38,105]. However, the effectiveness of automation remains limited by the lack of harmonized metadata across repositories [65,89,106].

Additional complementary approaches could further enhance discoverability. Persistent identifiers (e.g., DOIs) can ensure long-term accessibility, prevent broken links, and support dataset citation [13,67]. The adoption of persistent identifiers is essential for datagraphy, yet remains underdeveloped [13,19]. Assigning persistent identifiers guarantees long-term accessibility, facilitates citation, and prevents link rot caused by website migrations [107]. Metadata harmonization frameworks, such as the Data Tag Suite (DATS), also enable standardized descriptions of heterogeneous datasets and support automated search and retrieval [62,63,108].

Despite these advances, the adoption of standardized packaging and metadata frameworks remains limited, constraining automation, reproducibility, and scalability in dataset discovery [65,89,106].

Powered by Editorial Manager® and ProduXion Manager® from Aries Systems Corporation

6.2 Potential implications and future directions

The aforementioned structural challenges underscore the necessity of harmonizing both repository-level infrastructures and researcher-level practices.

6.2.1 Datagraphy as a systematic research methodology

Establishing datagraphy as a recognized research methodology could have profound implications across scientific disciplines. By promoting systematic dataset selection, datagraphy may enhance the reliability and reproducibility of data reuse research, mitigate selection bias, facilitate transparent dataset reuse, and foster interdisciplinary collaboration through improved dataset discoverability. As scientific data grow in volume and complexity, the need for a structured approach to dataset selection becomes increasingly urgent. Integrating datagraphy as a foundational research practice would ensure that dataset selection adheres to the same methodological rigor as literature reviews, ultimately strengthening the integrity and impact of data-driven discoveries.

6.2.2 Standardization efforts

To advance datagraphy, standardization efforts are essential, much as they have been for bibliographic search. Establishing reporting guidelines analogous to PRISMA [36] could be a viable strategy. Another option could be extending existing frameworks (e.g., adapting PRISMA for dataset discovery). Unified guidelines for cloud architectures [109], the promotion of common data elements [110,111], and the standardization of reporting formats and metadata sharing remain essential priorities [112]. Richly described metadata in machine-readable formats will enhance interoperability, enabling efficient data harvesting, attribution, and semantic understanding [77,107,110-112]. Ongoing harmonization initiatives, such as those in toxicology [85], should be further supported and expanded.

6.2.3 Aligning researcher and repository-level practices

For datagraphy to realize its full potential, it must operate in synergy with repository-level improvements. Sustainable and reproducible datagraphy requires alignment with community standards for metadata and packaging. This includes the adoption of standardized, machine-readable metadata schemas (e.g., DATS), harmonized vocabularies, persistent identifiers (e.g., DOIs), and lightweight packaging formats such as RO-Crates, BDBags, Frictionless Data Packages, or FDOs [39,62,63,80,94,95,100,105,113]. Integrating provenance-aware workflows and schema-validated metadata could further strengthen reproducibility and automation [99,114,115]. These implementations would provide the foundations for machine-actionable dataset discovery and ensure that datasets remain discoverable, comparable, and citable across repositories [39,95,106]. Repository-level innovations, including semantic search, canonical workflows, and federated queries across FAIR-compliant repositories, can further reduce manual search effort while increasing reproducibility [10,101,106,116].

6.2.4 FAIRness, reusability, and sustainability

Developing sustainable and trustworthy FAIR-compliant searchable data discovery resources (e.g., data repositories) is essential for supporting datagraphy. The creation of a comprehensive search index could enable dataset comparison [32]. However, empirical insights into what makes a dataset more reusable remain limited [2]. Guidelines such as FAIR, which promote universal metadata standards, are essential for dataset comparison and integration [19,76]. While measuring "FAIRness" is not yet an established practice, several efforts (e.g., the FAIR metrics group) are paving the way [2]. Validation frameworks such as FAIRO [103] and automated FAIRness metrics [38,105] could provide objective indicators of dataset quality. Implementing FAIR principles enhances discoverability and reuse, ensuring seamless dataset discovery, access, and integration across diverse research domains [76,77,107]. Complementing the FAIR principles with the TRUST (Transparency, Responsibility, User focus, Sustainability, and Technology) principles could provide a more comprehensive framework for data sustainability [93]. In addition, operationalizing the FAIR Principles alongside other standards, such as the CARE principles, can enhance machine actionability while also ensuring that data are used appropriately across the entire data lifecycle [68].

Integrating dataset search tools with bibliographic databases and data management platforms represents a promising step toward improving the findability of datasets [34].

Powered by Editorial Manager® and ProduXion Manager® from Aries Systems Corporation

For instance, databases such as Web of Science and PubMed enable users to filter search results specifically for data papers. This can be achieved via a dedicated filter box in Web of Science or by appending searches with the term data[filter] in PubMed. However, this functionality is not yet available in other bibliographic databases. In contrast, some literature repositories like PubMed Central offer more sophisticated search capabilities that allow users to limit queries to articles associated with data. This can be done by utilizing specialized search operators, including hassuppdata, hasdataavail, hasdatacitations, and hasassociateddata, which target papers containing data availability statements or supplemental data files.

6.2.5 Governance and community initiatives

Community-driven initiatives will play a central role in advancing datagraphy. Projects such as the RDA [20], DCAT [2], DATACC (https://www.datacc.org/en/), and schema.org [2] can enhance datagraphy by improving dataset discoverability and enabling federated searches across multiple data catalogs. A unified data service is needed to efficiently retrieve relevant and reliable datasets [32]. For example, Wikidata provides a community-maintained knowledge graph that supports semantic enrichment and linking across datasets [31,65]. Another example is the Global Data Sharing Initiative that proposed a pipeline that collects relevant information from diverse sources, integrates multiple sharing streams, and merges them into a unified dataset for statistical analysis or secure data examination [117]. The European initiative DataGEMS [118] aims to address this challenge by developing an advanced data discovery platform based on FAIR principles. DataGEMS will integrate data sharing, discovery, and analysis into a comprehensive ecosystem covering the entire data lifecycle (i.e., storage, management, discovery, analysis, and reuse). This EU-funded initiative involves twelve partners across eight countries working to create open-source tools that facilitate access to FAIR-by-design datasets. By promoting data FAIRness, DataGEMS will bridge the gap between data providers and users, fostering a more efficient and transparent data-sharing ecosystem. Complementary efforts, such as Make Data Count [19], further legitimize datasets as scholarly outputs, enabling proper citation and attribution alongside traditional publications.

6.2.6 AI integration in dataset discovery

The integration of AI-based tools for automated dataset discovery could represent a promising avenue for enhancing datagraphy. For example, DataGEMS will leverage state-of-the-art data management, natural language processing, and machine learning to support dataset discovery and analysis across diverse data modalities, including tabular data, text documents, knowledge graphs, and images [118]. AI-based tools such as DataScout [61] and large language models (LLMs) promise to enhance dataset discovery through query reformulation, semantic filtering, and relevance scoring [119-124]. These innovations complement, rather than replace, necessary repository-level changes such as metadata harmonization and packaging. Together, technological innovation and metadata harmonization must evolve in concert, rather than in isolation.

6.3 Conclusion

Datagraphy and community-driven efforts should be advanced in synergy. Datagraphy provides a structured methodology for the systematic identification and evaluation of datasets, whereas systemic solutions (e.g., metadata standardization) strengthen the underlying infrastructure that supports dataset discovery. As a reproducible, pragmatic, and researcher-centered approach, datagraphy offers a valuable means of navigating the fragmented landscape of dataset discovery. However, its full effectiveness depends on addressing repository heterogeneity through the implementation of standardized, machine-readable metadata, persistent identifiers, provenance-aware workflows, and FAIR-compliant packaging formats such as RO-Crates or FDOs. By aligning datagraphy with repository-level standards and community best practices, researchers can substantially improve dataset discoverability and foster sustainable reuse. In this way, datagraphy functions both as a practical methodology and as a conceptual framework that underscores the necessity of harmonized infrastructure. When integrated with FAIR-aligned repositories and robust reporting guidelines, datagraphy has the potential to transform dataset selection into a transparent, durable, and scalable research practice, thereby enabling researchers to engage more effectively and confidently with increasingly complex data landscapes.”.

Powered by Editorial Manager® and ProduXion Manager® from Aries Systems Corporation

	--<br>Reviewer #2<br><br>This paper addresses an important topic, and I suggest that it be published with some revisions. I have included some areas for revision below:<br><br>- Re3Data is a listing of data repositories, not a data repository itself. For example, if I search for 'cancer' in Re3Data, I will be shown data repositories that specialize in cancer, not datasets on cancer. In your example on Line 275, please clarify what the three resources you located in Re3Data and if they were datasets or data repositories. If you located a dataset by searching a repository you located through Re3Data, please clarify that as well.<br>Authors: Thank you for your review and your relevant comments. We value your time and effort.<br><br>You are correct that Re3Data is a catalog of data repositories rather than a data repository itself. It functions as a registry that provides information about research data repositories across disciplines. Researchers can use Re3Data to identify suitable repositories for depositing or accessing datasets, but the platform does not host data directly. To reflect this distinction more accurately and to avoid any ambiguity, we have replaced the term “data repository” with “data repository catalog” in Table 1. Please refer to Table 1.<br><br>It is true that Re3Data allows filtering by content type, including datasets (e.g., https://www.re3data.org/search?query=cancer&contentType40%5B%5D=dataset). However, to identify the three resources mentioned in our example, we did not apply the content type filter. Instead, we used the general query (https://www.re3data.org/search?query=exposom*), which yielded only three results. For transparency and reproducibility, we have added the URLs of the searches used for all 11 resources in the Supplementary Materials (see Table A.1) and expanded the description of our approach in the section “5. Illustrative and working example: the exposome case.” Please refer to Table A.1 and to lines 342-346 in the revised manuscript with track changes (or to lines 331-335 in the clean version): “For five resources, filter or facet options were used, with the “catalogue” facet for Epidemiologie – France, the data paper option in Web of Science (from the document types filter option), the dataset option in FAIRSharing (from the object types filter), OpenAIRE (from the document type filter), and Zenodo (from the resource types filter). Regarding Re3Data, the content type filter to select datasets was not used because only three results were obtained with the general search query.”.<br><br>- Similarly FAIRSharing is a listing of data repositories (referred to as databases on their site), data policies, and data standards. Please clarify in your example on Line 275 what 10 resources your located in FAIRSharing and if they were data repositories, data policies, or data standards. If you located a dataset by searching a repository you located through FAIRSharing, please clarify that as well.<br>Authors: Thank you for your relevant comment. You are correct that it provides listings of different types of resources, including data repositories (referred to as databases on the site), data policies, and data standards. Consequently, FAIRSharing is a data catalog (cf. Table 1). Importantly, FAIRSharing also allows users to identify datasets by applying the appropriate filters. For example, when searching for “exposome,” one can refine the results by selecting the object type “dataset”:<br>https://fairsharing.org/search?q=exposome&objectTypes=dataset.<br>To clarify this point, we added a sentence in the revised version of the manuscript. Please refer to lines 342-346 in the revised manuscript with track changes (or to lines 331-335 in the clean version): “For five resources, filter or facet options were used, with the “catalogue” facet for Epidemiologie – France, the data paper option in Web of Science (from the document types filter option), the dataset option in FAIRSharing (from the object types filter), OpenAIRE (from the document type filter), and Zenodo (from the resource types filter). Regarding Re3Data, the content type filter to select datasets was not used because only three results were obtained with the general search query.”.<br>For transparency and reproducibility, we also added the URLs of the searches used in the supplemental materials. Please refer to Table A.1.

Powered by Editorial Manager® and ProduXion Manager® from Aries Systems Corporation

	- Missing in your overall workflow is ways to locate dataset contained in supplementary materials. PubMed Central, for example, allows users to limit searches to papers that have associated data (either in a Data Availability Statement or in the supplemental files). I understand if you decide not to include that type of search in your PRISMA-like workflow, but you should at least mention that the workflow will not locate those types of materials.<br>Authors: We acknowledge that our current workflow does not systematically capture datasets that are available only in the supplementary materials of publications. To address this limitation, we modified the workflow and explicitly incorporated bibliographic databases that provide such functionality. In particular, PubMed and PubMed Central were added to Table 1. Please refer to Table 1.<br>Please also refer to lines 258-259 in the revised version of the manuscript with track changes (or to lines 248-249 in the revised version without track changes): "It is also possible to locate datasets associated with papers in bibliographic databases, such as PubMed.".<br>Please also refer to lines 658-668 in the revised version of the manuscript with track changes (or to lines 601-609 in the revised version without track changes): "Integrating dataset search tools with bibliographic databases and data management platforms represents a promising step toward improving the findability of datasets [34]. For instance, databases such as Web of Science and PubMed enable users to filter search results specifically for data papers. This can be achieved via a dedicated filter box in Web of Science or by appending searches with the term data[filter] in PubMed. However, this functionality is not yet available in other bibliographic databases. In contrast, some literature repositories like PubMed Central offer more sophisticated search capabilities that allow users to limit queries to articles associated with data. This can be done by utilizing specialized search operators, including hassuppdata, hasdataavail, hasdatacitations, and hasassociateddata, which target papers containing data availability statements or supplemental data files.".<br><br>- Please clarify how the 11 dataset discovery platforms were chosen (line 243).<br>Authors: We followed your suggestion and clarified the criteria used to select the 11 dataset discovery platforms. Please refer to lines 335-342 in the revised version of the manuscript with track changes (or to lines 324-331 in the revised version without track changes): "The selection of the 11 dataset discovery resources was empirical and aimed at capturing, as far as possible, the heterogeneity and fragmentation of the current landscape. We therefore included different categories of resources: a web crawler (Google Dataset Search), a data aggregator (DataMed), domain-specific repositories (Epidemiologie – France and TEDI), a data-focused journal (Scientific Data), a bibliometric database (Web of Science), a data platform (OpenAIRE), a trusted data portal (FAIRSharing), as well as widely used general-purpose repositories (Dryad and Zenodo) and a repository catalog (Re3Data). Whenever possible, we preferentially selected resources that were openly accessible or freely available.".<br><br>- Consider mentioning the CARE Principles as another ethical consideration. The CARE Principles were created by Native Americans to help dictate how research data generated from indigenous populations should be governed and made available.<br>Authors: Thank you for your relevant comment. We followed your suggestion by including the CARE Principles as an additional example of ethical consideration. Please refer to lines 269-274 in the revised version of the manuscript with track changes (or to lines 258-264 in the revised version without track changes): "The central question at this stage is whether, and under what conditions, a dataset can be legally and ethically reused. For instance, the CARE Principles were created by Native Americans to guide how research data generated from Indigenous populations should be governed and made available [68-70].".<br>Please also refer to lines 653-656 in the revised version of the manuscript with track changes (or to lines 597-600 in the revised version without track changes): "In addition, operationalizing the FAIR Principles alongside other standards, such as the CARE Principles, can enhance machine actionability while also ensuring that data are used appropriately across the entire data lifecycle [68]".<br><br>- Around Line 380, you mention that Web of Science allows for filtering for data papers. Another existing connection between literature databases and data is the ability to filter PubMed Central results to papers that have associated data either described in a Data Availability or included in a supplemental file.

Powered by Editorial Manager® and ProduXion Manager® from Aries Systems Corporation

	Authors: You are right, thank you. We provided more details to acknowledge this point. Please refer to lines 658-668 in the revised version of the manuscript with track changes (or to lines 601-609 in the revised version without track changes): “Integrating dataset search tools with bibliographic databases and data management platforms represents a promising step toward improving the findability of datasets [34]. For instance, databases such as Web of Science and PubMed enable users to filter search results specifically for data papers. This can be achieved via a dedicated filter box in Web of Science or by appending searches with the term data[filter] in PubMed. However, this functionality is not yet available in other bibliographic databases. In contrast, some literature repositories like PubMed Central offer more sophisticated search capabilities that allow users to limit queries to articles associated with data. This can be done by utilizing specialized search operators, including hassuppdata, hasdataavail, hasdatacitations, and hasassociateddata, which target papers containing data availability statements or supplemental data files.”.<br><br>- You mentioned DataMed on Line 187. I do not think DataMed is regularly maintained at this point and you may not want to include it. For example, it does not allow you to search for data past 2024, and it only has 2 datasets from 2024.<br>Authors: We appreciate your comment regarding the current status of DataMed. The most recent update on its website is from November 2023. DataMed was initially funded by the NIH through the Big Data to Knowledge (BD2K) program as part of the BioCADDIE (Biomedical and HealthCAre Data Discovery and Indexing Engine) project. As this funding has now ended, no new records appear to have been added since 2024. Although DataMed is no longer actively maintained, we think that it still provides useful insights into dataset discovery strategies. To reflect this limitation, we added clarifying sentences in the discussion section.<br>Please refer to lines 532-534 in the revised version of the manuscript with track changes (or to lines 496-498 in the revised version without track changes): “Without sustained funding and community support, data search tools risk becoming outdated or abandoned, which compromises their reliability and long-term utility for researchers. For example, DataMed has not received new additions since 2024 [62,63,82,83].”.<br><br>- Similar to the note above, another challenge worth mentioning is that many data infrastructure projects are reliant on grant funding, meaning that some data catalogs or data search tools go out of date or are no longer maintained.<br>Authors: You are right. We have expanded the discussion section to explicitly address this important challenge. Please refer to lines 530-534 in the revised version of the manuscript with track changes (or to lines 494-498 in the revised version without track changes): “Sustainability poses an additional systemic risk. Many data-sharing platforms lack long-term funding models, which threatens not only the durability and accessibility of datasets but also the continued relevance of the information they host [Kilgus et al. 2024]. Without sustained funding and community support, data search tools risk becoming outdated or abandoned, which compromises their reliability and long-term utility for researchers. For example, DataMed has not received new additions since 2024 [62,63,82,83].”.<br><br>- In Table 1, you mention the NYU Data Catalog. It is not a government data portal. It is a data catalog that includes some government data.<br>Authors: You are right, we apologize for this mistake. We modified Table 1 accordingly. Please refer to Table 1.<br><br>Thank you for the opportunity to read your paper.
**Additional Information:**	
**Question**	**Response**
Are you submitting this manuscript to a special series or article collection?	No
**Experimental design and statistics**<br><br>Full details of the experimental design and	Yes

statistical methods used should be given in the Methods section, as detailed in our Minimum Standards Reporting Checklist. Information essential to interpreting the data presented should be made available in the figure legends.<br><br>Have you included all the information requested in your manuscript?	
**Resources**<br><br>A description of all resources used, including antibodies, cell lines, animals and software tools, with enough information to allow them to be uniquely identified, should be included in the Methods section. Authors are strongly encouraged to cite Research Resource Identifiers (RRIDs) for antibodies, model organisms and tools, where possible.<br><br>Have you included the information requested as detailed in our Minimum Standards Reporting Checklist?	Yes
**Availability of data and materials**<br><br>All datasets and code on which the conclusions of the paper rely must be either included in your submission or deposited in publicly available repositories (where available and ethically appropriate), referencing such data using a unique identifier in the references and in the "Availability of Data and Materials" section of your manuscript.<br><br>Have you have met the above requirement as detailed in our Minimum Standards Reporting Checklist?	Yes
GigaScience has policies and guidelines in place for the use of generative AI-writing tools such as ChatGPT. If you have used such writing tools to assist with writing the manuscript this must be	No

declared and cited in the text. Authors should not list AI-writing tools and other AI-assisted technologies as an author or co-author and should acknowledge that they are fully responsible for text generated or refined by AI-writing tools.<p><br><br>A summary of use (particularly in the introduction or among methods) needs to be included at the end of the paper, and the outputs should also be included as a supplementary file hosted in GigaDB or other open repositories. Please read our guidelines for more information. <p><br><br>By submitting to GigaScience, you are aware of the journal's AI-writing tools policy, and if you have declared use of such tools below, you have acknowledged this where appropriate in your manuscript and have made a summary of use and outputs available. <p><br><b>AI-assisted writing tools have been used in the preparation of this manuscript?	

Manuscript
Click here to access/download;Manuscript;Manuscript_2025-10-21.docx

Title: Datagraphy: toward a systematic approach to dataset discovery

Authors

Pascal Petit[1,*]; Nicolas Vuillerme[1,2]

[1] Univ. Grenoble Alpes, AGEIS, 38000 Grenoble, France

[2] Institut Universitaire de France, Paris, France

Correspondence (present address)

* Corresponding author at: Pascal Petit. Laboratoire AGEIS - Université Grenoble Alpes. Bureau 315. Bâtiment Jean Roget. UFR de Médecine. Domaine de La Merci. 38706 La Tronche Cedex, France. E-mail address: pascal.petit@univ-grenoble-alpes.fr (P. Petit). Telephone: + 33 4 76 63 71 04.

ORCID

Pascal Petit [0000-0001-9015-5230]; Nicolas Vuillerme [0000-0003-3773-393X]

Abstract

Data has become central to scientific discovery. While primary data collection remains vital, there is growing recognition of the benefits of reusing existing datasets. However, identifying suitable datasets for specific research questions is increasingly difficult due to the fragmentation and heterogeneity of the big data ecosystem. Despite the expansion of data sharing, efficient dataset discovery remains elusive, with limited empirical research on how datasets are identified, interpreted, and reused. Current dataset search practices often lack standardization, leading researchers to rely on convenience rather than systematic criteria. Unlike bibliographic research, dataset selection lacks a formal methodology, increasing the risks of bias, inefficiencies, and reduced generalizability. To address this gap, we introduce datagraphy, a structured approach to dataset identification and evaluation. Analogous to bibliographic methods but designed for datasets, datagraphy encompasses not only discovery but also critical assessment of dataset quality, relevance, interoperability, completeness, sustainability, and ethical use. By formalizing dataset search as a research practice, datagraphy seeks to improve transparency, reproducibility, and interdisciplinary collaboration, while also reducing research redundancy and environmental impact. We present a nine-step framework to operationalize datagraphy and explore challenges such as inconsistent metadata and variability among dataset discovery tools. This framework provides a foundation for systematically and reproducibly identifying and synthesizing reusable datasets. To demonstrate the application of the proposed framework, we conducted a datagraphic search focused on the exposome. We discuss major challenges faced by datagraphy with respect to metadata availability, repository heterogeneity, dataset accessibility, and dataset quality, and highlight how datagraphy could enhance transparency, reproducibility, and efficiency at the researcher level. Datagraphy is intended to complement repository-level improvements. Aligning researcher practices with standardized, machine-readable metadata, persistent identifiers, artificial intelligence integration, and lightweight packaging frameworks such as RO-Crates and FAIR Digital Objects could enable automated discovery and sustainable dataset reuse. By integrating structured researcher-level methodology with systemic improvements and community efforts, datagraphy could offer a scalable approach for systematic, FAIR-aligned data-driven research across disciplines.

Keywords: dataset discovery; data reuse; datagraphy; datagraphic search; research practice; information; exposome; big data; open data; open science

1. Background

Data is omnipresent, shaping nearly every aspect of our lives [1]. The digital revolution and the increasing reliance on technology have led to an exponential surge in data generation, creating a "data big bang." This transformation has marked the onset of the fourth industrial revolution, in which information has become a central pillar and data is widely regarded as the "new gold" of the 21st century [2,3]. Over the past years, data has evolved from being a mere byproduct of digital activities to a highly valuable asset whose worth increases with use [2]. The web now provides access to millions of data sources [2], which are becoming increasingly vast, complex, and heterogeneous as societies undergo continuous digitization [4]. These sources originate from diverse domains and are of different natures [5], including contextual data (e.g., air pollution), person-generated data (e.g., wearables, social media), administrative health data (e.g., electronic health records), and synthetic data (e.g., digital twins) [6,7].

This data revolution is profoundly reshaping the scientific landscape. In recent years, the scientific field has undergone an epistemological shift, transitioning from knowledge-driven to data-driven research [1,4,8]. Data has become a cornerstone of scientific discovery and is increasingly regarded as a form of scientific currency (data commodification) [4,8-10]. This shift has driven efforts to move away from isolated data silos towards more integrated, accessible, and reusable data ecosystems, where multiple data sources can be used or needed to address a research question [11]. To support this transition, major efforts have been made to establish mandates and standards promoting data sharing [12-14], including the adoption of the FAIR (Findable, Accessible, Interoperable, and Reusable) principles [2,15-17]. The ultimate goal of the FAIR principles is to optimize the reuse of digital assets populating the Internet [2,15-17].

While the collection of new data remains a central focus, there is growing recognition of the substantial benefits associated with reusing existing datasets [12,18,19]. For example, the European Union and the World Health Organization (WHO) have introduced supportive frameworks such as the Research Data Alliance (RDA) [20], the European Open Data Directive (Directive (EU) 2019/1024), the Data Governance Act (DGA) [21,22], the General Data Protection Regulation (GDPR) [21,23], and the European Health Data Space [21,23,24]. Nearly half of researchers frequently use data generated by other scientists [25]. Data reuse not only facilitates the validation and replication of findings but also

enables the exploration of extended or novel research questions [18,19]. When disseminated with sufficient quality and contextual information, data from diverse scientific communities can contribute to new knowledge through cross-disciplinary insights. Data reuse has become an established practice that mitigates unnecessary duplication of research, accelerates scientific progress, and optimizes resource allocation in terms of time, effort, staff, and costs, while limiting risks to research participants [9,12,18,19]. In this regard, the reuse of data represents a resource-efficient approach that helps reduce the environmental footprint of research [26,27]. Furthermore, leveraging existing data allows researchers to address high-impact scientific questions that would otherwise require substantial time and resources [28].

Beyond access, data reuse relies on effective data discovery [15], a fundamental aspect of the FAIR principles that involves identifying and locating relevant datasets [15-17]. The ability to find appropriate datasets is a prerequisite for their reuse [29]. However, locating relevant datasets remains challenging, often requiring researchers to navigate multiple resources, review numerous publications, and directly contact dataset owners or study authors [11,14,15,29-32]. As data sharing becomes more common, the complexity of dataset discovery is becoming increasingly evident [10,15,33]. Traditionally, researchers identified relevant datasets by consulting the literature, attending conferences, and engaging with colleagues [10]. In today’s data-rich environment, web searches have become the primary method for locating datasets [10,11,31], but other resources also exist. However, the success of such searches varies widely, depending on the expertise of the researcher, the tools employed, and, to some extent, chance [10]. Despite the growing need for efficient dataset discovery, there is still limited large-scale empirical evidence on how researchers locate, access, interpret, and reuse datasets [34]. Consequently, the importance of improving dataset discovery within the scientific community is paramount.

For decades, scientific research has relied on bibliographic searches to systematically identify and synthesize relevant literature, which is an essential process for supporting hypotheses, contextualizing findings, and ensuring methodological rigor [35,36]. However, in the era of data-driven science [20,37], the ability to select appropriate datasets is equally critical for research validity and reproducibility. Despite the increasing availability of large-scale, open-access datasets across scientific disciplines, researchers still lack standardized methodologies for systematically identifying, evaluating, and

selecting datasets that best address their specific research questions [6,11]. To bridge this gap, we introduce the concept of "*datagraphy*" (or "*datagraphic search*"), a structured approach analogous to bibliographic research but focused on datasets rather than publications. The proposed methodology is designed to support researchers in the systematic identification, evaluation, and documentation of datasets that align with their research objectives. By providing a structured approach, we aim to strengthen reproducibility, transparency, and methodological rigor in data reuse. Here, we highlight the potential of datagraphy as well as hurdles that need to be overcome.

2. Defining datagraphy

We propose to define datagraphy as the systematic process of identifying, evaluating, and documenting datasets most suitable for addressing specific research questions, akin to how bibliographic research is conducted to identify relevant scientific literature. Datagraphy extends beyond simple dataset discovery. It further advantageously incorporates the assessment of dataset quality, relevance, completeness, and ethical considerations [11], thereby ensuring that selected datasets align with research objectives in a rigorous and transparent manner. Datagraphy further emphasizes the detailed documentation of dataset provenance, licensing conditions, and metadata completeness, which could facilitate reproducibility and support integration across research projects [38,39]. By formalizing datagraphy as a research practice, we advocate enhancing reproducibility, mitigating selection bias, and improving the overall integrity of dataset reuse investigations.

3. Rationale and existing gaps

A variety of resources are already available for conducting datagraphy (Table 1). Existing datasets are dispersed across an increasing number of repositories, article supplements, academic journals, websites, and other platforms, each employing distinct metadata structures, data standards, and search functionalities [15,30,32,40,41].

For instance, domain-agnostic dataset search engines, such as *Google Dataset Search*, enable broad searches before directing users to specific repositories, where more targeted queries and dataset exploration can be conducted [31,34,42-45]. Data catalogs [14], such as *OccupationalCohorts.net* [46]

and *OccupationalExposureTools.net* [47], which provide structured inventories of data assets through curated metadata records. In addition to repositories and catalogs, data papers [48,49] serve as valuable resources for dataset discovery [50]. These scientific publications detail dataset collection, processing, and validation methodologies, thereby informing the research community about their availability, characteristics, and reuse potential [19]. Data papers undergo peer review and are published in both general scientific journals and specialized data journals, such as *GigaScience*, *Data in Brief*, and *Scientific Data* [19,51]. To improve data accessibility, many traditional scientific journals now require data availability statements and mandate that datasets be stored either as supplementary materials or in designated repositories [25]. Numerous data repositories and registries exist [52], including *Re3Data* [15,32,53], *Zenodo* [17,51], and *Dataverse* [18]. Government agencies also provide access to datasets, such as those available through the *NYU Libraries Data Sources* [14,43] and other national or regional data portals [2,54]. In addition, open platforms like *GitHub* and *Kaggle* host a range of datasets across multiple domains [2]. All of the aforementioned resources vary in scope, ranging from institutional (e.g., university-level) to international initiatives [29]. Some are domain-specific, such as *TEDI* for toxicology and public health [55], while others, like *Re3Data*, span multidisciplinary research areas [15,32,53] (Table 1). Access to these resources also differs, with some that are openly available (e.g., *Dataverse*), whereas others require institutional affiliation (e.g., *Web of Science*) or subscription-based access (e.g., *Dimensions.ia*). This heterogeneity underscores the necessity of a standardized approach to ensure comprehensive and unbiased dataset identification.

While the aforementioned resources facilitate dataset discovery, they primarily function as search engines rather than providing systematic evaluation frameworks [11,31]. Unlike literature searches, dataset selection is complicated by repository-level differences in metadata, submission procedures, and access policies, making reproducibility challenging [56]. In contrast, bibliographic research methodologies, such as systematic reviews and meta-analyses, follow rigorous protocols for literature selection and synthesis [36]. Current dataset search strategies lack standardization, as researchers often select datasets based on convenience and opportunity rather than through systematic assessment. Unlike bibliographic research, to the best of our knowledge, no formalized methodology exists for dataset selection, making dataset integration and comparison particularly challenging [19]. The absence of

structured dataset selection methodologies increases the risk of opportunistic dataset use, potentially introducing biases and limiting the generalizability of research findings.

The development of a standardized framework for dataset identification and evaluation is essential for strengthening the reliability and impact of dataset reuse research. Such a framework could not only enhance reproducibility but also maximize the scientific value of existing datasets and facilitate interdisciplinary research. To achieve these goals, it should incorporate explicit quality metrics, assess metadata completeness, and account for accessibility and compliance with FAIR principles, thereby ensuring that datasets are both discoverable and suitable for secondary analysis [38,39].

Table 1: Examples of digital searchable dataset discovery resources

Name	Type	Country	Domain	RA	DL	Characteristics
4TU.ResearchData	Data repository	International	Multidisciplinary	FA	yes	10 322 datasets
Auctus	Data search engine	International	Multidisciplinary	FA	yes	
CANUE (Canadian Urban Environmental Health Research Consortium)	Data portal	Canada	Environmental health	FR	yes	
DANS	Data repository	Netherlands	Multidisciplinary	FA	yes	313 603 datasets
Data	Data-focused journal	International	Multidisciplinary	OA	yes	
Data Catalog	Data catalog	EU	Multidisciplinary	FA	no	274 datasets
Data Citation Index	Dataset aggregator	International	Multidisciplinary	Sub	?	>15 million datasets, 453 repositories
Data Europa	Governmental data portal	EU	Multidisciplinary	FA	yes	1 857 283 datasets, 195 catalogues
Data in Brief	Data-focused journal	International	Multidisciplinary	OA	yes	
Data Repository Finder	Search tool	International	Multidisciplinary	FA	no	25 repositories
Data Science	Data-focused journal	International	Multidisciplinary	OA	yes	
Data Science Journal – Codata	Data-focused journal	International	Multidisciplinary	OA	yes	
data.gouv	Governmental data portal	France	Multidisciplinary	FA	yes	61 418 datasets
data.world	Data platform	International	Multidisciplinary	Sub	?	
Database Commons	Data catalog	International	Biological	FA	no	7 347 databases
Datacite	Data repository catalog	International	Multidisciplinary	FA	no	>20 million datasets, 3309 repositories
DataHub	Data catalog	International	Multidisciplinary	FA	yes	
DataMed	Open source data discovery system	International	Biomedical	FA	no	1 280 165 datasets, 49 repositories
DataOne	Data aggregator	International	Environment	FA	no	
Dataverse	Data repository	International	Multidisciplinary	FA	no	466 000 datasets
Dimensions.ia	Publication aggregator with dataset filter	International	Multidisciplinary	Sub	?	29 million datasets
Dryad	Data repository	International	Multidisciplinary	FA	yes	50 000 data publications
Earth data NASA	Data repository	International	Environment	FA	yes	10 749 datasets
EMIF Catalogue	Data catalog	EU	Health	FR	?	480 datasets
Environmental Data Initiative (EDI) Repository	Data repository	International	Environment	FA	yes	
Portail Epidémiologie – France	Data catalog	France	Public health	FA	no	1 098 datasets
EUDat	Data repository	EU	Multidisciplinary	FA	no	
European Health Data Space (EHDS)	Data platform	EU	Health	FA	?	
European Open Science Cloud (EOSC)	Data platform	EU	Multidisciplinary	FA	?	
F1000Research	Data-focused journal	International	Multidisciplinary	OA	yes	
FAIR environmental and health registry (FAIREHR)	Data registry	International	Public health, environmental health	FA	no	
FAIRDOM	Data management platform	International	Biology	FR	?	
FAIRsharing	Data catalog	International	Multidisciplinary	FA	no	2 318 datasets
FigShare	Data repository	International	Multidisciplinary	FA	yes	2 107 300
GigaDB	Data aggregator	International	Multidisciplinary	FA	yes	2 682 datasets
GigaScience	Data-focused journal	International	Multidisciplinary	OA	yes	
GitHub	Data platform	International	Multidisciplinary	FA	yes	
Google dataset search	Domain agnostic data search engine	International	Multidisciplinary	FA	no	>25 million datasets
Green data for health (GD4H)	Data catalog	France	Environment, environmental health	FA	no	177 datasets
Harvard Data Science Review	Data-focused journal	International	Multidisciplinary	OA	yes	
Kaggle	Data platform	International	Multidisciplinary	FR	yes	438 322 datasets
Mendeley Data	Data aggregator	International	Multidisciplinary	FA	yes	>20 million datasets
NYU Data Catalog	Data catalog	US	Health	FA	no	426 datasets
OccupationalCohorts.net	Data catalog	EU	Health	FA	no	164 datasets
OccupationalExposureTools.net	Data catalog	EU	Health	FA	no	11 datasets
OmicsDI	Data catalog	International	Health	FA	yes	4 914 243 datasets
Open Access Infrastructure for Research in Europe (OpenAIRE)	Research platform	EU	Multidisciplinary	FA	no	74 million datasets, 10 932 repositories
Open Science Framework (OSF)	Management platform	International	Multidisciplinary	FA	yes	2 600 datasets
OpenDoar	Repository catalog	International	Multidisciplinary	FA	no	5 982 repositories
Our World in Data	Data repository	International	Multidisciplinary	FA	yes	
PubMed	Bibliographic database	International	Multidisciplinary	FA	no	
PubMed Central (PMC)	Bibliographic database	International	Biomedical and life	FA	no	
Registry of Research Data Repositories (Re3Data)	Data repository catalog	International	Multidisciplinary	FA	no	3 331 repositories

RoHub	Research object management platform	International	Multidisciplinary	FA	yes	3 363 research objects
ScholeXplorer	Data platform	International	Multidisciplinary	Sub	?	1.15 billion datasets
Scientific Data	Data-focused journal	International	Multidisciplinary	OA	yes	
TEDI (Toxicological and Exposure Database Inventory)	Data catalog	International	Public health, toxicology, environmental health	FA	no	1 055 datasets
UK data service	Governmental data portal	UK	Multidisciplinary	FA	no	9 877 datasets
Web of Science	Bibliographic database	International	Multidisciplinary	Sub	no	
World Health Organization	Data portal	International	Health	FA	yes	
Zenodo	Data repository	International	Multidisciplinary	FA	yes	403 919 datasets

Note: DL: indicates whether datasets can be directly downloaded from the discovpubery resource; FA: freely accessible; FR: free registration required; OA: open-access publications; RA: type of access to the dataset discovery resource; Sub: subscription required. The searchable dataset resource names are provided as hyperlinks.

4. Proposed framework for datagraphic search

The principles of systematic searching, traditionally associated with systematic reviews and meta-analyses [36], can be effectively extended to dataset discovery. This structured approach aims to identify all relevant datasets within resource constraints, enhance transparency in the search process, and ensure reproducibility. By providing a rigorous and replicable framework for dataset selection, it mitigates subjective biases and fosters collaboration across disciplines, benefiting not only researchers but also industry professionals, stakeholders, and other interested parties. Consequently, datagraphy has the potential to empower a wide range of users (e.g., researchers and policymakers) by facilitating access to diverse types of knowledge. At the same time, it must be acknowledged that this framework operates within the limitations of existing repository practices and infrastructures, which remain heterogeneous and largely unstandardized. Thus, the proposed methodology should be understood as a pragmatic, researcher-centric approach that enhances efficiency and rigor in dataset discovery, while recognizing that systemic repository-level improvements are still necessary to fully address the underlying challenges.

To ensure the relevance and utility of a datagraphic search, authors are encouraged to provide a transparent, comprehensive, and accurate account of the rationale behind the search, the methodology employed (including dataset identification and selection criteria), and the key findings (e.g., dataset characteristics). To operationalize this process, we propose a nine-step framework that mirrors the systematic approach used in bibliographic research (Figure 1). The framework is intended to structure researcher activity and enhance reproducibility and efficiency.

Step 1 involves defining the research question, which represents a fundamental component of scientific inquiry [57]. The purpose of this step is to clearly articulate the rationale for seeking a dataset, whether for comparison, validation, or the development of a new study [10]. In some cases, this may also entail integrating data from multiple sources to construct a new dataset [31]. Establishing a well-defined research question helps determine the specific issues that should be addressed through dataset analysis but also provides direction for subsequent stages of datagraphy. Existing guidelines, such as those provided by the Joanna Briggs Institute, offer valuable support by applying structured frameworks, such

as the population, concept, and context criteria (PCC) [58]. This ensures that the scope of the datagraphic search remains coherent, transparent, and aligned with the overarching scientific objectives.

Step 2 involves specifying dataset requirements, that is, the characteristics a dataset must possess to serve the formulated purpose(s). These may include essential variables, study population, timeframes, geographic scope, granularity, and data formats [10]. Importantly, initial requirements and constraints may evolve as the search progresses [10]. By clearly defining these parameters, researchers can more effectively target datasets that are directly relevant to the research question while excluding irrelevant datasets early in the process, thereby improving both efficiency and rigor.

Step 3 involves defining the dataset search strategy, analogous to approaches used in bibliographic literature searches. Queries should be strategically designed while accounting for the heterogeneity of repository functionalities [10]. Several recommendations developed for systematic reviews can provide useful guidance in balancing between sensitivity and specificity [59]. However, because relevant information is often distributed across multiple datasets and resources, search strategies frequently need to be adapted [11,31]. For example, administrative health databases such as the French National Health Data System (SNDS) [60] may lack key epidemiological variables, such as environmental factors (e.g., air pollution, climate data) [6]. In such cases, distinct search strategies may be required to identify both administrative health records and complementary contextual datasets that can be integrated. Dataset search systems typically rely on query languages and information retrieval principles, in which information needs are expressed through keywords or faceted filters based on metadata attributes [31]. Some platforms, such as *DataONE*, support semantic technologies that automatically expand user-entered keywords to include relevant synonyms [10]. In contrast, if a search portal lacks this functionality, users must manually include appropriate synonyms to ensure comprehensive results [10,32]. Several systems also allow the use of search operators (e.g., OR, AND) to refine results, but several queries are often necessary to achieve adequate coverage [11,31]. Based on initial outputs, queries may need to be broadened or narrowed, mirroring the iterative refinement process common in bibliographic searching [10]. Repository-specific facets and filters, such as those for data format, type of analysis, or availability, can further improve efficiency by enabling the rapid identification of usable datasets [10,11,31]. Overall, dataset discovery is inherently iterative. Successive query reformulation,

combined with the use of filters and the inspection of preliminary results, progressively sharpens the search strategy [61]. Nevertheless, most repositories currently rely primarily on keyword-based searches over metadata, which often fail to capture the full content and context of datasets. This limitation highlights the need for iterative refinement and careful adaptation of strategies during the search process [30].

Step 4 focuses on dataset discovery, that is, the process of identifying datasets potentially suitable for the defined research purpose(s) [11]. Researchers can leverage various data repositories, platforms, catalogs, data papers, and other resources to identify potentially eligible/relevant datasets. Searches may be conducted globally using services such as *Google Dataset Search* [31], *Auctus* [30], and *DataMed* [30,31,62,63], or locally within individual repositories [31]. The choice of the dataset discovery resource(s) should consider factors such as domain relevance, repository trustworthiness, and technical features. In many cases, discipline-specific repositories provide the most effective means of discovery, as researchers with similar interests are more likely to store and share datasets within these specialized platforms [15]. Domain-specific portals further streamline the search process by offering interfaces and filters tailored to the needs of particular research fields [10]. Data aggregators such as *DataONE* and *DataMed* allow users to search multiple repositories through a single interface [10,31,63]. However, not all data discovery resources are equally trustworthy. For example, repositories certified by the *CoreTrustSeal* must meet 16 criteria related to accessibility, usability, reliability, and long-term data preservation [10,64]. Understanding the standards and practices a searchable dataset resource applies to its data and metadata can increase confidence in dataset quality and reusability [10]. Beyond repositories, datasets can also be located through publications using resources such as the Data Citation Index [11]. Data citation itself plays a critical role in making datasets findable and accessible by providing persistent identifiers and descriptive metadata, which ensure reliable referencing, tracking, and reuse [11,65]. It is also possible to locate datasets associated with papers in bibliographic databases, such as *PubMed*. Finally, a recent study has also outlined eleven practical tips for dataset discovery, providing a useful starting point or foundational guidance for this endeavor [10]. Other helpful guidelines and examples have also been proposed [66].

Step 5 evaluates whether a dataset is accessible and under what conditions. While some datasets can be downloaded directly from the web, others may require a subscription, direct contact with the authors, or approval from data custodians. Key access considerations include data format, file size, transfer costs, availability of mirrors, and data proximity [67]. Evaluating these factors at an early stage helps avoid wasted effort and ensures compliance with repository policies and usage restrictions [67].

Step 6 pertains to ensuring ethical and legal compliance. This includes verifying adherence to data-sharing policies, privacy regulations, and licensing constraints to safeguard responsible dataset use. The central question at this stage is whether, and under what conditions, a dataset can be legally and ethically reused. For instance, the CARE (Collective benefit, Authority to control, Responsibility, Ethics) principles were created by Native Americans to guide how research data generated from Indigenous populations should be governed and made available [68-70]. While open-access datasets generally involve minimal restrictions, sensitive data may require institutional review board (IRB) approval or multifactor authentication [62].

Step 7 involves assessing dataset relevance (eligibility) by evaluating its alignment with the research objectives, including considerations such as scope, granularity, and contextual applicability. An initial review of the metadata is often sufficient to determine whether a dataset meets the preliminary requirements defined in Steps 1 and 2 [10,66]. Some searchable dataset resources, such as *Figshare*, offer preview features that allow users to quickly evaluate the dataset's structure and content. Ideally, metadata should be accompanied by comprehensive documentation to support a thorough evaluation of the dataset's relevance and fitness for use. This includes details on data collection methods, quality assurance procedures, and prior applications of the data [10]. If a dataset fails to meet any of the established criteria, it may be advisable to exclude it from further consideration [10]. Data summarization tools have also been proposed to help in this endeavor [66].

Step 8 requires evaluating data quality by examining attributes such as provenance, completeness, representativeness, interoperability, generalizability, timeliness, validity, and potential limitations, including missing data or measurement errors [28,66,71]. Assessing dataset quality and fitness for purpose is paramount, as shared data may be erroneous or unsuitable for reuse [45]. Key considerations include whether the dataset contains the necessary variables to address the research question, whether

the data collection and its sampling methodology are appropriate, how variables are defined and measured, and whether the sample size is sufficient to ensure adequate statistical power. Other critical factors include the extent of missing data and, in longitudinal studies, the degree of loss to follow-up [10]. To facilitate a robust quality assessment, predefined metrics (e.g., accuracy, completeness, consistency, timeliness, currency, conformance, and uniqueness) can be applied [72]. This step also aims to assess whether data management or wrangling is required and, if so, to determine its potential scope [73]. The feasibility of dataset reuse may decline if the effort required to standardize a dataset (i.e., make it research-ready) for research purposes is disproportionately high relative to its potential benefits [73]. In addition, ensuring high dataset quality is essential for deriving meaningful insights [72]. For instance, a recent study offers guidance on evaluating dataset quality in the context of machine learning [72], which can serve as a valuable starting point for implementing Step 8 of the proposed framework. Reusability indicators such as machine-readability, data annotation, and data validation further ensure that datasets are reported in ways consistent with their intended use [38]. Understanding how datasets were produced, including their provenance and relationships to other sources, is also paramount for determining whether a dataset can be reused [65]. Repository-level quality indicators, FAIRness scores, and standardized metrics can provide objective measures of completeness, representativeness, and overall usability, supporting informed decisions about dataset selection and reuse.

Step 9 involves transparently documenting the dataset identification and selection process, following a structured approach similar to that of systematic reviews (e.g., a PRISMA-like flowchart [36]) (Figure 2). This documentation ensures clarity and reproducibility, ultimately strengthening the reliability of dataset reuse research. All steps, including queries, refinements, access conditions, and integration procedures, should be recorded in a reproducible and transparent manner.

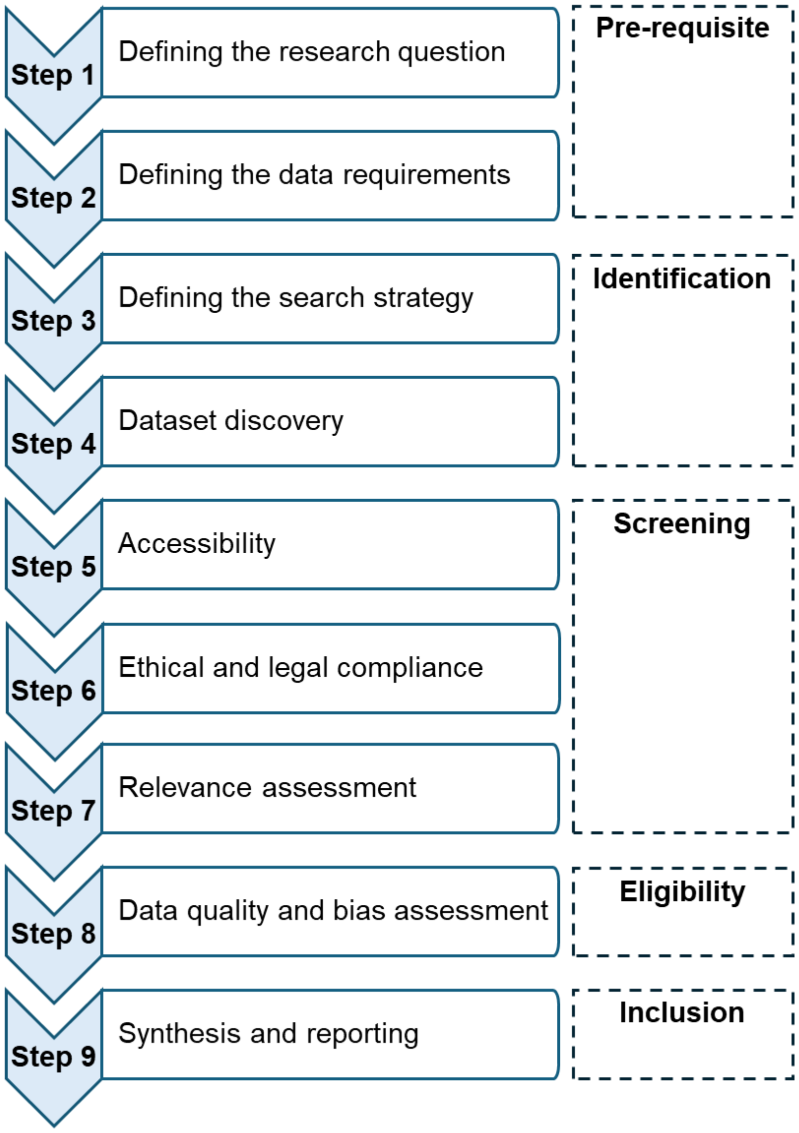

Figure 1: Proposed datagraphy framework

Operationalization of the datagraphy concept with a nine-step approach that mirrors the systematic method used in bibliographic research.

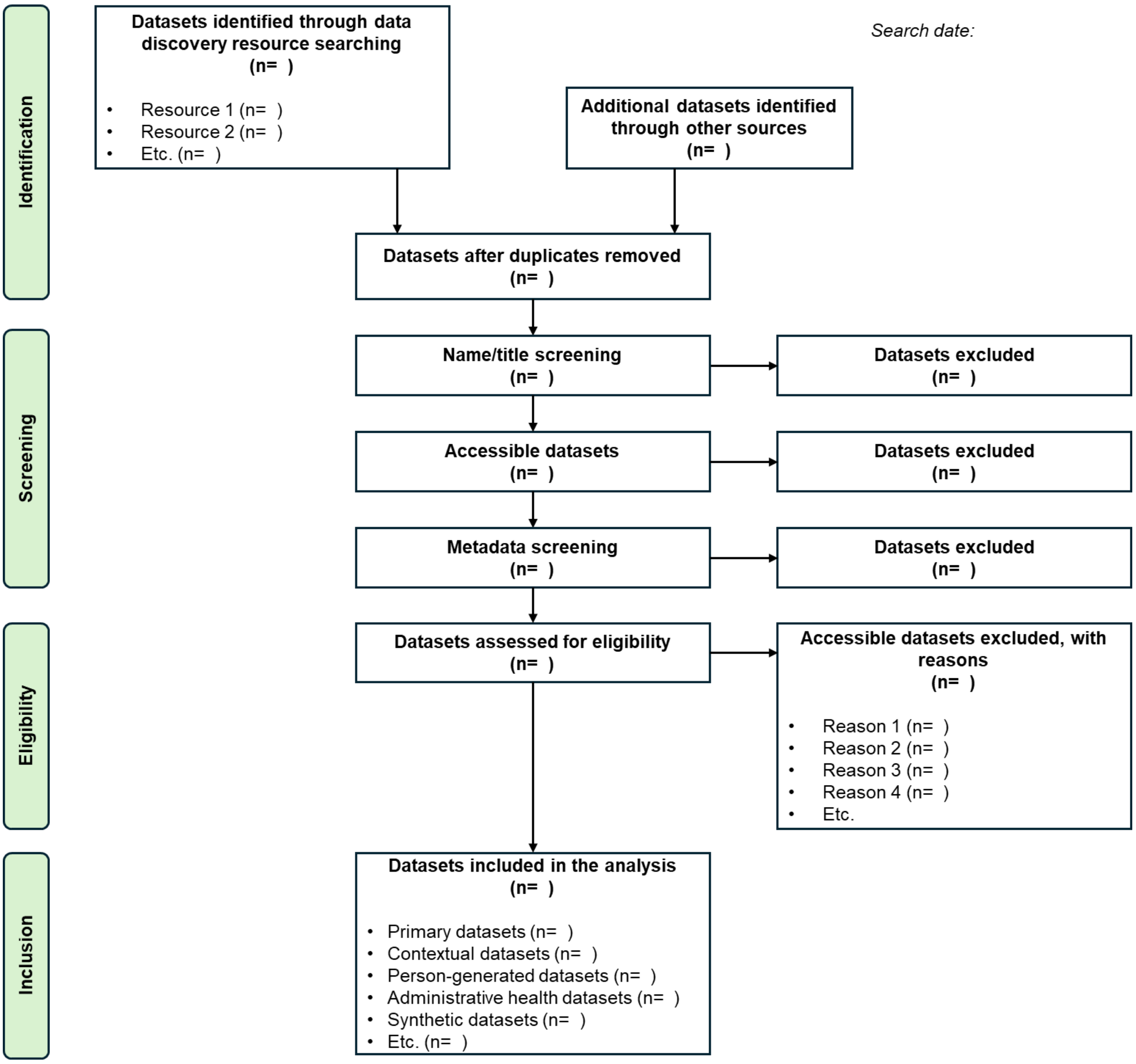

Figure 2: PRISMA-like flowchart for datagraphy

Flowchart illustrating the datagraphy concept.

5. Illustrative and working example: the exposome case

To demonstrate the application of the proposed framework, we conducted a datagraphic search focused on the exposome. The research question was formulated using the Joanna Briggs Institute's PCC framework [58]. The population of interest included any human subjects exposed to environmental factors. The concept encompassed any studies involving at least one health-related outcome. No restrictions were applied for context.

When possible, we used the same search query (i.e., exposom*) as in a prior bibliometric analysis on the exposome [7]. For searchable dataset resources that did not support wildcard characters, we used the simplified query "exposome." The search was carried out across 11 dataset discovery platforms, following a process inspired by the Preferred Reporting Items for Systematic Reviews and Meta-Analyses (PRISMA) guidelines [36] and the practical recommendations outlined by Gregory et al. [10] and Koesten et al. [66]. For this example, screening was performed by a single author (PP). To minimize bias due to temporal variability in dataset availability, all data were collected on the same day, April 10, 2025.

The selection of the 11 dataset discovery resources was empirical and aimed at capturing, as far as possible, the heterogeneity and fragmentation of the current landscape. We therefore included different categories of resources: a web crawler (*Google Dataset Search*), a data aggregator (*DataMed*), domain-specific repositories (*Epidemiologie – France* and *TEDI*), a data-focused journal (*Scientific Data*), a bibliographic database (*Web of Science*), a data platform (*OpenAIRE*), a trusted data portal (*FAIRSharing*), as well as widely used general-purpose repositories (*Dryad and Zenodo*) and a repository catalog (*Re3Data*). Whenever possible, we preferentially selected resources that were openly accessible or freely available. For five resources, filter or facet options were used, with the "catalogue" facet for *Epidemiologie - France*, the data paper option in *Web of Science* (from the document types filter), the dataset option in *FAIRSharing* (from the object types filter), *OpenAIRE* (from the document type filter), and *Zenodo* (from the resource types filter). Regarding *Re3Data*, the content type filter to select datasets was not used because only three results were obtained with the general search query.

To ensure the relevance and representativeness of the included datasets, titles/names, metadata, and dataset content were assessed based on predefined inclusion and exclusion criteria (Table 2). When titles or metadata lacked sufficient information, full-content screening was conducted. Datasets were included if they addressed the human exposome. Exclusion criteria encompassed datasets involving only animal or in vitro data, those irrelevant to the exposome concept, or those lacking individual-level data on exposures, outcomes, and participant characteristics. For this illustrative example, we deliberately excluded datasets that contained adverse outcome data without environmental exposure/factor

information, even though some could potentially be linked to contextual environmental datasets (e.g., via geographic data).

Table 2: Criteria used for the dataset selection

**Question**	**Description**	**Answer**	
		no	yes/can't tell
**Stage 1: Screening dataset name/title**			
Q_{11}	Does the name/title mention terms related to exposome?	0	1
Q_{12}	Does the name/title mention terms related to humans?	0	1
Q_{13}	Is the name/title in English or French?	0	1
$S_1 = Q_{11} \times Q_{12} \times Q_{13}$; *Dataset eligible for stage 2 if* $S_1 = 1$			
**Stage 2: Dataset accessibility**			
Q_{21}	Is the dataset freely accessible?	0	1
Q_{22}	Can the dataset be reused for research purposes?	0	1
$S_2 = Q_{21} \times Q_{22}$; *Dataset eligible for stage 3 if* $S_2 = 1$			
**Stage 3: Screening dataset metadata**			
Q_{31}	Is there a data dictionary explaining the dataset content?	0	1
Q_{32}	Does the metadata mention terms related to exposome?	0	1
Q_{33}	Does the metadata mention terms related to humans?	0	1
Q_{34}	Is there any individual data available?	0	1
Q_{35}	Is the data real (not synthetic)?	0	1
$S_3 = Q_{31} \times Q_{32} \times Q_{33} \times Q_{34} \times Q_{35}$; *Dataset eligible for stage 4 if* $S_3 = 1$			
**Stage 4: Screening dataset content**			
Q_{41}	Is the data in English or French?	0	1
Q_{42}	Does the dataset structure/format allow its reuse?	0	1
Q_{43}	Does the dataset contain individual data?	0	1
Q_{44}	Does the dataset contain an ID for each participant?	0	1
Q_{45}	Does the dataset contain exposure data?	0	1
Q_{46}	Does the dataset contain participants' characteristics?	0	1
Q_{47}	Does the dataset contain outcome data (e.g., presence or absence of a disease)?	0	1
$S_4 = Q_{41} \times Q_{42} \times Q_{43} \times Q_{44} \times Q_{45} \times Q_{46} \times Q_{47}$; *Dataset eligible for inclusion if* $S_4 = 1$			
Score = $S_1 \times S_2 \times S_3 \times S_4$; ***Dataset selected for review/analysis if score = 1***			

Note: Q: question, S: score.

Results from the datagraphic search are presented in Figure 3, with step-by-step details provided in Table A.1 (Supplementary Materials). Of the 11 searchable dataset resources, six supported the use of the wildcard query "exposom*." A total of 322 datasets were initially retrieved. After removing 109 duplicates (34%), 213 unique records remained for screening. Duplicate identification was challenging due to variations in dataset names and the presence of subsets nested within larger datasets. In cases where subsets offered no additional unique information, only the original dataset was retained.

Title-based screening excluded 85 datasets (40%). Of the remaining 128, 22 (17%) were inaccessible, resulting in 106 datasets for metadata screening. Of these, 96 (91%) were excluded, most commonly due to the absence of individual-level data (n=67, 70%) or a lack of exposure/environmental data (n=20, 21%).

The final screening phase involved a full content review of the remaining 10 datasets, from which 4 met the inclusion criteria. These included two datasets from the National Health and Nutrition Examination Survey (NHANES) [49,74], one from a Pakistani cohort study [48], and one from the EXPOsOMICS Personal Exposure Monitoring Study [75]. Dataset quality assessment was not conducted for this illustrative example.

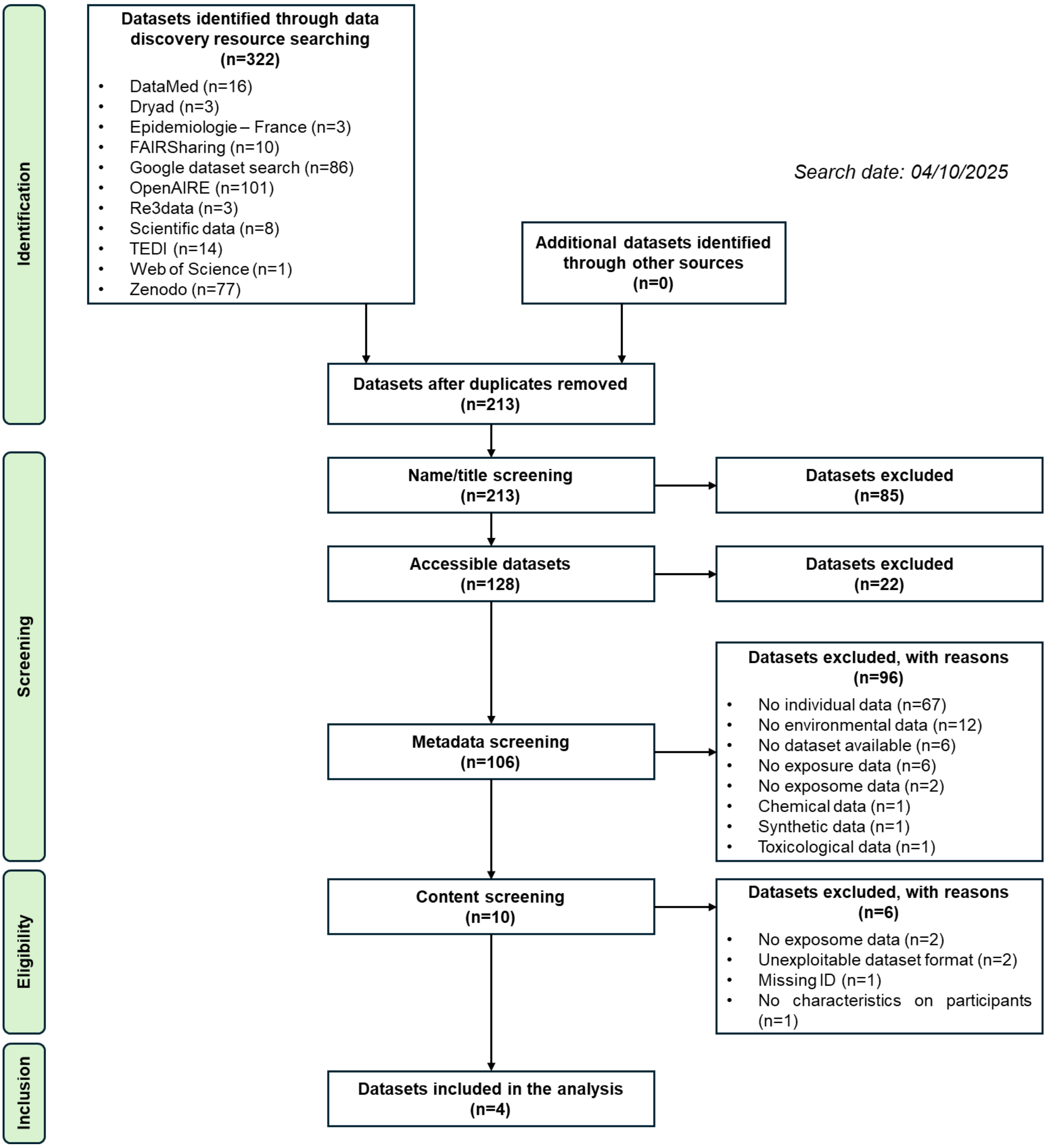

Figure 3: PRISMA-like flowchart of the exposome datagraphic search

Flowchart summarizing the results of the exposome datagraphic search.

6. Discussion

6.1 Challenges

The successful implementation of datagraphy faces multiple challenges.

6.1.1 Metadata availability, heterogeneity, and quality

One of the primary obstacles is the limited availability of metadata, which is essential for assessing the existence and characteristics of datasets. Current deposition practices impose only minimal restrictions on the descriptors used [17]. The substantial variability in metadata and the lack of standardized protocols for data collection, storage, and format/structure across repositories create major barriers to the effective identification and reuse of datasets. While datagraphy could help researchers navigate this fragmented landscape more systematically, it does not resolve the underlying heterogeneity. In the absence of consensus on core repository services, each organization tends to implement systems aligned with its specific goals [64], making cross-repository integration challenging. For example, in data papers and data-focused journals, publication requirements and the amount of data that can be attached vary by journal, making it challenging to find comprehensive and useful content. In addition, limited accessibility and machine-readability of existing metadata, in particular for population health data, further hamper effective dataset discovery and reuse [76-78].

Metadata quality represents a further critical bottleneck. Because dataset search relies heavily on metadata, it is imperative that descriptions are accurate and consistent [31]. Metadata are essential for establishing dataset existence, scope, and suitability for reuse, yet they are frequently incomplete, restricted to high-level descriptors, inconsistent, or non-standardized [20,30,31]. Metadata quality also varies across disciplines and repositories, reflecting differences in cultural practices and curation priorities [71]. Errors, inconsistencies, and incompleteness in metadata are common, sometimes diverging from the underlying dataset [30]. Consequently, relying solely on metadata constrains discoverability and hampers systematic evaluation of dataset fitness for purpose [30,31].

6.1.2 Data discovery infrastructure and landscape

One major issue for dataset discovery is the broad freedom repositories have to implement their own discovery procedures, metadata standards, and data packaging approaches [2,64]. This heterogeneity creates structural challenges that go beyond the capacity of individual researchers to address. While existing resources accept a wide variety of data types and formats, they generally do not attempt to integrate, harmonize, or assess the quality of deposited data. Datasets may be incomplete, sometimes intentionally so [25]. Complex datasets, such as collections of tables, longitudinal records, or multi-modal data, remain poorly supported [45]. Certain dataset types, including administrative health records, lack comprehensive catalogs and are underrepresented in existing data repositories [6]. Searchable dataset discovery resources, whether general repositories or domain-specific catalogs, also differ in functionality and coverage, including subject matter, geographical and temporal scope, language, and scientific domain [2,35]. Consequently, the data ecosystem is becoming increasingly fragmented and heterogeneous, creating a "needle in a haystack" paradox that further complicates dataset discovery [17], in particular in domains where data lakes may contain tens of thousands of tables [61].

Dataset search is largely keyword-based, restrictive, and iterative, often requiring multiple query reformulations to assess relevance [31,61]. Such keyword-based approaches, which rely on limited metadata, are insufficient for comprehensive dataset search and discovery [11,30,31]. They fail to capture dataset granularity, provenance, and methodological context [31,61]. Many repositories only support simple keyword-based queries, which limits users' ability to express complex information needs [30]. Although some tools allow searches for spreadsheets or published data in formats such as *CSV* or *JSON*, most do not support complex datasets, such as collections of tables, text, or temporal data [11,31]. In addition, existing searchable data discovery resources often exhibit limited data discovery capabilities and provide only superficial query responses [11,31,61]. Translating search strategies and user queries across searchable dataset resources with differing syntax requirements can be a complex and time-consuming task [59]. To address this issue, the adoption of standardized vocabularies (e.g., taxonomies tailored to domain-specific terminology) is essential. One promising direction is the development of web-based search systems leveraging semantic metadata, as recently proposed in the field of information science for literature searches of scientific publications [79]. Current standards such as *Wikidata*,

Schema.org, and *Dublin Core* provide structured vocabularies for describing digital resources in a consistent, machine-readable way, while domain-specific ontologies enable richer semantic annotation [31,32,44,65,80,81]. There is a growing need for richer, content-aware indexing and semantic search methods [11,30,61].

Several initiatives have developed dataset discovery indexes (DDIs) and search engines to mitigate repository heterogeneity and search limitations. Examples include *DataMed*, designed as a "*PubMed* for datasets" [62,63,82,83]; *OmicsDI*, which aggregates resources across proteomics, genomics, and metabolomics [40]; *Auctus*, a web crawler and search engine dedicated to datasets for data augmentation in machine learning [30]; and *Google Dataset Search*, which indexes a broad range of datasets published on the web [30,31]. These search engines attempt to index heterogeneous repositories by exposing standardized metadata fields. Most repositories, however, still support only simple searches over metadata [30], although some, such as *Auctus*, enable more complex queries incorporating spatial, temporal, and integration constraints based on dataset content [30]. While these approaches demonstrate the feasibility of federated search, they remain constrained by the heterogeneity and incompleteness of the underlying metadata [11,31]. Advanced approaches, including semantic search, artificial intelligence (AI)-assisted query reformulation [61], and content-aware indexing, offer potential improvements [11,30,61]. Nevertheless, search effectiveness continues to be limited primarily by metadata heterogeneity and repository-specific practices [11,31].

6.1.3 Data accessibility, legal, and ethical considerations

Challenges also extend to repository functionality and scope. Dataset documentation and access conditions vary tremendously across resources, making selection difficult for researchers. Dataset accessibility is further complicated by licensing, ethical, and legal constraints. Licenses, data-use agreements (DUAs), and authentication requirements introduce additional barriers and shape who can access datasets and under what conditions [62]. Open-access datasets may be freely available, but many sensitive datasets require IRB approval, registered access, or multifactor authentication, adding layers of complexity and potential delays [60,62]. The time required to acquire existing datasets and obtain IRB approval can also vary considerably [28]. While some datasets can be directly downloaded from

the web (e.g., Comparative Toxicogenomics Database [84]), others require multiple layers of permissions and security measures (e.g., SNDS [60]), and in certain cases, data must be analyzed within a dedicated data processing environment (e.g., SNDS [60]). These examples illustrate the wide diversity of access conditions researchers must navigate. Furthermore, national and international ethical and legal obligations can restrict data sharing, grouped analyses, and data deposition [25,85]. Regulations such as the European GDPR require organizations to implement robust data protection measures, including data retention and deletion protocols, which can impede data reuse efforts and may result in substantial financial penalties in case of non-compliance [23,86].

Finally, access to published data is not always guaranteed due to broken links, missing metadata, or a lack of author cooperation [25]. For example, in a large-scale study of around 900 articles published in *Nature* and *Science* between 2000 and 2019, 61% of papers that included "data available upon request" statements did not provide the data when contacted [25]. Broken links, missing metadata, or author unresponsiveness therefore remain major obstacles.

6.1.4 Data quality and reusability

Researchers frequently encounter poorly described and non-standardized data, which limits reusability [20]. Assessing dataset fitness is often difficult, as available metadata rarely capture essential attributes such as granularity, provenance, or methodological context [31,61]. As a result, substantial time and effort are often required to manually sift through large volumes of irrelevant datasets.

Another major challenge in assessing data quality and reusability lies in the original data collection methods and processes (i.e., data capture), which are beyond the control of secondary users and can highly vary [72,87]. In addition to variability in metadata completeness and repository practices, the lack of standardized indicators or score systems for assessing dataset reusability (e.g., FAIRness) and quality further complicates efforts to identify the most appropriate datasets for specific research questions [2,19,76]. Practical considerations such as transit costs, storage, and access proximity may also influence dataset reusability [67].

Sharing data via supplementary materials remains common practice and is highly encouraged by journals [88]. However, unlike public repositories, supplementary files are not necessarily persistently

discoverable or archived. They frequently lack persistent identifiers (e.g., DOIs or accession numbers), making them vulnerable to link rot and content drift [88]. Moreover, supplementary datasets are often available only in aggregated or summary form, rather than as raw or individual-level data, which limits systematic reuse. Publishing datasets solely as linked data or supplementary materials is insufficient to support reproducibility and reuse, as such approaches fail to capture lifecycle information, provenance, versioning, and methodological context [89-91].

6.1.5 Data visibility, data decay, and sustainability

Systemic challenges such as dataset invisibility, data decay, broken links, and paywalls exacerbate discovery difficulties [25]. Some datasets remain inaccessible behind paywalls, making them invisible to many researchers [35], while others are never deposited in open repositories, resulting in so-called invisible data or dark data [14] and increasing the risk of data graveyards (i.e., unused data) [92]. Organizations frequently store vast numbers of tables and records in data lakes [61]. Over time, the availability of originally accessible data tends to decline (a phenomenon known as data decay), which can ultimately lead to data loss [25]. Estimates suggest that up to 80% of archived scientific research data are lost within 20 years [67].

Sustainability poses an additional systemic risk. Many data-sharing platforms lack long-term funding models, which threatens not only the durability and accessibility of datasets but also the continued relevance of the information they host [93]. Without sustained funding and community support, data search tools risk becoming outdated or abandoned, which compromises their reliability and long-term utility for researchers. For example, *DataMed* has not received new additions since 2024 [62,63,82,83]. Some repositories risk becoming unsustainable "data lakes," where uncurated, poorly described data accumulate without integration or retrieval mechanisms [35]. Without investment in long-term infrastructure, persistent identifiers, harmonized metadata standards, and robust governance structures, repository heterogeneity, metadata gaps, and fragmentation will persist [56]. In the absence of such measures, both discoverability and reuse remain severely undermined.

6.1.6 Standardized metadata frameworks and packaging

Efforts to improve data discoverability across the social sciences, biomedical domains, omics research, and public datasets emphasize the need for metadata harmonization, advanced search interfaces, and distributed resource indexing [32,40]. Implementing repository-wide FAIR-compliant practices and semantic metadata frameworks could help address existing limitations [38,39,81,94,95]. While the FAIR principles provide an overarching vision for dataset reuse, their adoption at the repository level remains highly uneven [20,64]. Two recent reviews, covering 35 [20] and 25 [64] data repositories, respectively, identified interoperability and sustainability as major obstacles to achieving FAIR compliance.

Repositories differ in the metadata standards they apply, the search interfaces they provide, and the approaches they adopt for data packaging. This diversity produces structural fragmentation that cannot be addressed at the level of individual researchers [2,64]. Most attempts to mitigate these limitations have focused on standardized, machine-readable metadata and packaging approaches. By adopting FAIR digital objects (FDOs) or research object (RO)-Crate packages, repositories could ensure that metadata, provenance, licensing, and dataset structure are consistently available, machine-actionable, and interoperable, thereby facilitating automated discovery and reuse across platforms [38,39,81,91,94-98].

ROs are structured bundles of data, methods, and metadata that capture lifecycle information, ownership, versioning, attribution, provenance, quality, and methodological context [89-91]. RO-Crate extends this concept by providing a lightweight, machine-readable packaging framework for aggregating research artifacts with their metadata and relationships, creating a multimodal scholarly knowledge graph that can help "*FAIRify*" and combine metadata from existing resources [91,94,97,99]. FDOs, in turn, are digital objects explicitly designed to be FAIR, embedding metadata, provenance, and other descriptive information in a machine-actionable format [38,65]. Other packaging standards, such as BDBags (Big Data Bags), provide mechanisms for content enumeration, fixity checking, and lightweight referencing without requiring centralized hosting [45,100]. The integration of canonical workflows, including pipelines based on the Common Workflow Language (CWL), could further support reproducibility and enable systematic evaluation of search queries and analyses across repositories [101,102].

Validation frameworks, reusability metrics, and machine-readable metadata derived from RO-Crates or FDOs could enable semi-automated or automated assessments of dataset fitness and quality [38,103,104]. For instance, the FAIRO framework has been proposed to measure the compliance of ROs with FAIR criteria [103], while reusability indicators and automated quality checks have been incorporated into RO and RO-Crate packaging [38,105]. However, the effectiveness of automation remains limited by the lack of harmonized metadata across repositories [65,89,106].

Additional complementary approaches could further enhance discoverability. Persistent identifiers (e.g., DOIs) can ensure long-term accessibility, prevent broken links, and support dataset citation [13,67]. The adoption of persistent identifiers is essential for datagraphy, yet remains underdeveloped [13,19]. Assigning persistent identifiers guarantees long-term accessibility, facilitates citation, and prevents link rot caused by website migrations [107]. Metadata harmonization frameworks, such as the Data Tag Suite (DATS), also enable standardized descriptions of heterogeneous datasets and support automated search and retrieval [62,63,108].

Despite these advances, the adoption of standardized packaging and metadata frameworks remains limited, constraining automation, reproducibility, and scalability in dataset discovery [65,89,106].

6.2 Potential implications and future directions

The aforementioned structural challenges underscore the necessity of harmonizing both repository-level infrastructures and researcher-level practices.

6.2.1 Datagraphy as a systematic research methodology

Establishing datagraphy as a recognized research methodology could have profound implications across scientific disciplines. By promoting systematic dataset selection, datagraphy may enhance the reliability and reproducibility of data reuse research, mitigate selection bias, facilitate transparent dataset reuse, and foster interdisciplinary collaboration through improved dataset discoverability. As scientific data grow in volume and complexity, the need for a structured approach to dataset selection becomes increasingly urgent. Integrating datagraphy as a foundational research practice would ensure that dataset

selection adheres to the same methodological rigor as literature reviews, ultimately strengthening the integrity and impact of data-driven discoveries.

6.2.2 Standardization efforts

To advance datagraphy, standardization efforts are essential, much as they have been for bibliographic search. Establishing reporting guidelines analogous to PRISMA [36] could be a viable strategy. Another option could be extending existing frameworks (e.g., adapting PRISMA for dataset discovery). Unified guidelines for cloud architectures [109], the promotion of common data elements [110,111], and the standardization of reporting formats and metadata sharing remain essential priorities [112]. Richly described metadata in machine-readable formats will enhance interoperability, enabling efficient data harvesting, attribution, and semantic understanding [77,107,110-112]. Ongoing harmonization initiatives, such as those in toxicology [85], should be further supported and expanded.

6.2.3 Aligning researcher and repository-level practices

For datagraphy to realize its full potential, it must operate in synergy with repository-level improvements. Sustainable and reproducible datagraphy requires alignment with community standards for metadata and packaging. This includes the adoption of standardized, machine-readable metadata schemas (e.g., DATS), harmonized vocabularies, persistent identifiers (e.g., DOIs), and lightweight packaging formats such as RO-Crates, BDBags, Frictionless Data Packages, or FDOs [39,62,63,80,94,95,100,105,113]. Integrating provenance-aware workflows and schema-validated metadata could further strengthen reproducibility and automation [99,114,115]. These implementations would provide the foundations for machine-actionable dataset discovery and ensure that datasets remain discoverable, comparable, and citable across repositories [39,95,106]. Repository-level innovations, including semantic search, canonical workflows, and federated queries across FAIR-compliant repositories, can further reduce manual search effort while increasing reproducibility [10,101,106,116].

6.2.4 FAIRness, reusability, and sustainability

Developing sustainable and trustworthy FAIR-compliant searchable data discovery resources (e.g., data repositories) is essential for supporting datagraphy. The creation of a comprehensive search index could enable dataset comparison [32]. However, empirical insights into what makes a dataset more reusable remain limited [2]. Guidelines such as FAIR, which promote universal metadata standards, are essential for dataset comparison and integration [19,76]. While measuring "*FAIRness*" is not yet an established practice, several efforts (e.g., the *FAIR metrics* group) are paving the way [2]. Validation frameworks such as FAIRO [103] and automated FAIRness metrics [38,105] could provide objective indicators of dataset quality. Implementing FAIR principles enhances discoverability and reuse, ensuring seamless dataset discovery, access, and integration across diverse research domains [76,77,107]. Complementing the FAIR principles with the TRUST (Transparency, Responsibility, User focus, Sustainability, and Technology) principles could provide a more comprehensive framework for data sustainability [93]. In addition, operationalizing the FAIR Principles alongside other standards, such as the CARE principles, can enhance machine actionability while also ensuring that data are used appropriately across the entire data lifecycle [68].

Integrating dataset search tools with bibliographic databases and data management platforms represents a promising step toward improving the findability of datasets [34]. For instance, databases such as *Web of Science* and *PubMed* enable users to filter search results specifically for data papers. This can be achieved via a dedicated filter box in *Web of Science* or by appending searches with the term *data[filter]* in *PubMed*. However, this functionality is not yet available in other bibliographic databases. In contrast, some literature repositories like *PubMed Central* offer more sophisticated search capabilities that allow users to limit queries to articles associated with data. This can be done by utilizing specialized search operators, including *hassuppdata*, *hasdataavail*, *hasdatacitations*, and *hasassociateddata*, which target papers containing data availability statements or supplemental data files.

6.2.5 Governance and community initiatives

Community-driven initiatives will play a central role in advancing datagraphy. Projects such as the *RDA* [20], *DCAT* [2], *DATACC*, and *schema.org* [2] can enhance datagraphy by improving dataset discoverability and enabling federated searches across multiple data catalogs. A unified data service is

needed to efficiently retrieve relevant and reliable datasets [32]. For example, *Wikidata* provides a community-maintained knowledge graph that supports semantic enrichment and linking across datasets [31,65]. Another example is the Global Data Sharing Initiative that proposed a pipeline that collects relevant information from diverse sources, integrates multiple sharing streams, and merges them into a unified dataset for statistical analysis or secure data examination [117]. The European initiative *DataGEMS* [118] aims to address this challenge by developing an advanced data discovery platform based on FAIR principles. *DataGEMS* will integrate data sharing, discovery, and analysis into a comprehensive ecosystem covering the entire data lifecycle (i.e., storage, management, discovery, analysis, and reuse). This EU-funded initiative involves twelve partners across eight countries working to create open-source tools that facilitate access to FAIR-by-design datasets. By promoting data FAIRness, *DataGEMS* will bridge the gap between data providers and users, fostering a more efficient and transparent data-sharing ecosystem. Complementary efforts, such as *Make Data Count* [19], further legitimize datasets as scholarly outputs, enabling proper citation and attribution alongside traditional publications.

6.2.6 AI integration in dataset discovery

The integration of AI-based tools for automated dataset discovery could represent a promising avenue for enhancing datagraphy. For example, *DataGEMS* will leverage state-of-the-art data management, natural language processing, and machine learning to support dataset discovery and analysis across diverse data modalities, including tabular data, text documents, knowledge graphs, and images [118]. AI-based tools such as *DataScout* [61] and large language models (LLMs) promise to enhance dataset discovery through query reformulation, semantic filtering, and relevance scoring [119-124]. These innovations complement, rather than replace, necessary repository-level changes such as metadata harmonization and packaging. Together, technological innovation and metadata harmonization must evolve in concert, rather than in isolation.

6.3 Conclusion

Datagraphy and community-driven efforts should be advanced in synergy. Datagraphy provides a structured methodology for the systematic identification and evaluation of datasets, whereas systemic solutions (e.g., metadata standardization) strengthen the underlying infrastructure that supports dataset discovery. As a reproducible, pragmatic, and researcher-centered approach, datagraphy offers a valuable means of navigating the fragmented landscape of dataset discovery. However, its full effectiveness depends on addressing repository heterogeneity through the implementation of standardized, machine-readable metadata, persistent identifiers, provenance-aware workflows, and FAIR-compliant packaging formats such as RO-Crates or FDOs. By aligning datagraphy with repository-level standards and community best practices, researchers can substantially improve dataset discoverability and foster sustainable reuse. In this way, datagraphy functions both as a practical methodology and as a conceptual framework that underscores the necessity of harmonized infrastructure. When integrated with FAIR-aligned repositories and robust reporting guidelines, datagraphy has the potential to transform dataset selection into a transparent, durable, and scalable research practice, thereby enabling researchers to engage more effectively and confidently with increasingly complex data landscapes.

List of abbreviations

AI: artificial intelligence

CARE: Collective benefit, Authority to control, Responsibility, Ethics

CWL: common workflow language

DATS: Data Tag Suite

DDI: dataset discovery index

DGA: Data Governance Act

DOI: digital identifier of an object

DUA: data-use agreement

EU: European Union

FAIR: Findable, Accessible, Interoperable, and Reusable

FDO: FAIR digital object

GDPR: General Data Protection Regulation

IRB: Institutional Review Board

LLM: large language model

NHANES: National Health and Nutrition Examination Survey

PCC: population, concept, and context criteria

PRISMA: Preferred Reporting Items for Systematic Reviews and Meta-Analyses

RDA: Research Data Alliance

RO: research object

SNDS: French National Health Data System

TRUST: Transparency, Responsibility, User focus, Sustainability, and Technology

WHO: World Health Organization

Acknowledgments

None.

Declaration of interests

The authors declare that they have no known competing financial interests or personal relationships that could have appeared to influence the work reported in this paper.

Funding

This work was partially supported by funding from the French government, managed by the National Research Agency (ANR) under the France 2030 program [ANR-23-IACL-0006] and under the "Investissements d'avenir" program [ANR-10-AIRT-0005 and ANR-15-IDEX-0002]. The funders played no role in study design, data collection, analysis and interpretation of data, or the writing of this manuscript.

Author's contribution

Pascal Petit: Conceptualization, Methodology, Software, Validation, Investigation, Data Curation, Writing - Original Draft, Writing - Review & Editing, Visualization, Supervision, Project administration, Funding acquisition.

Nicolas Vuillerme: Methodology, Validation, Writing - Review & Editing, Supervision, Project administration, Funding acquisition, Resources.

Data availability

The authors confirm that the data supporting the findings of this study are available within the article.

References

1. Pastorino R, De Vito C, Migliara G, Glocker K, Binenbaum I, Ricciardi W, et al. Benefits and challenges of Big Data in healthcare: an overview of the European initiatives. Eur J Public Health. 2019;29(Supplement_3):23-27. doi:10.1093/eurpub/ckz168.
2. Koesten L, Vougiouklis P, Simperl E, Groth P. Dataset Reuse: Toward Translating Principles to Practice. Patterns (N Y). 2020;1(8):100136. doi:10.1016/j.patter.2020.100136.
3. Peterson ED. Machine Learning, Predictive Analytics, and Clinical Practice: Can the Past Inform the Present?. JAMA. 2019;322(23):2283-2284. doi:10.1001/jama.2019.17831.
4. Li C, Huang M. Environmental Sustainability in the Age of Big Data: Opportunities and Challenges for Business and Industry. Environ Sci Pollut Res Int. 2023;30(56):119001-119015. doi:10.1007/s11356-023-30301-5.
5. Näher AF, Vorisek CN, Klopfenstein SAI, Lehne M, Thun S, Alsalamah S, et al. Secondary data for global health digitalisation. Lancet Digit Health. 2023;5(2):e93-e101. The Lancet. Digital health, 5(2), e93–e101. doi:10.1016/S2589-7500(22)00195-9.
6. Petit P, Vuillerme N. Leveraging Administrative Health Databases to Address Health Challenges in Farming Populations: Scoping Review and Bibliometric Analysis (1975-2024). JMIR Public Health Surveill. 2025;11:e62939. doi:10.2196/62939.
7. Petit P, Vuillerme N. Global research trends on the human exposome: a bibliometric analysis (2005-2024). Environ Sci Pollut Res Int. 2025;32(13):7808-7833. doi:10.1007/s11356-025-36197-7.
8. Tenopir C, Rice NM, Allard S, Baird L, Borycz J, Christian L, et al. Data sharing, management, use, and reuse: Practices and perceptions of scientists worldwide. PLoS One. 2020;15(3):e0229003. doi:10.1371/journal.pone.0229003.
9. Chabilall J, Brown Q, Cengiz N, Moodley K. Data as scientific currency: Challenges experienced by researchers with sharing health data in sub-Saharan Africa. PLOS Digit Health. 2024;3(10):e0000635. doi:10.1371/journal.pdig.0000635.

10. Gregory K, Khalsa SJ, Michener WK, Psomopoulos FE, de Waard A, Wu M. Eleven quick tips for finding research data. PLoS Comput Biol. 2018;14(4):e1006038. doi:10.1371/journal.pcbi.1006038.
11. Paton NW, Chen J, Wu Z. Dataset Discovery and Exploration: A Survey. ACM Comput Surv. 2023;56(4):1-37. doi:10.1145/3626521.
12. Doolan DM, Winters J, Nouredini S. Answering Research Questions Using an Existing Data Set. Med Res Arch. 2017;5(9).
13. Pierce HH, Dev A, Statham E, Bierer BE. Credit data generators for data reuse. Nature. 2019;570(7759):30-32. doi:10.1038/d41586-019-01715-4.
14. Yee M, Surkis A, Lamb I, Contaxis N. The NYU Data Catalog: a modular, flexible infrastructure for data discovery. J Am Med Inform Assoc. 2023;30(10):1693-1700. doi:10.1093/jamia/ocad125.
15. Contaxis N, Clark J, Dellureficio A, Gonzales S, Mannheimer S, Oxley PR, et al. Ten simple rules for improving research data discovery. PLoS Comput Biol. 2022;18(2):e1009768. doi:10.1371/journal.pcbi.1009768.
16. Vesteghem C, Brøndum RF, Sønderkær M, Sommer M, Schmitz A, Bødker JS, et al. Implementing the FAIR Data Principles in precision oncology: review of supporting initiatives. Brief Bioinform. 2020;21(3):936-945. doi:10.1093/bib/bbz044.
17. Wilkinson MD, Dumontier M, Aalbersberg IJ, Appleton G, Axton M, Baak A, et al. The FAIR Guiding Principles for scientific data management and stewardship. Sci Data. 2016;3:160018. doi:10.1038/sdata.2016.18.
18. Dul J, van Raaij E, Caputo A. Advancing scientific inquiry through data reuse: Necessary condition analysis with archival data. Strateg Chang. 2024;33:35–40. doi:10.1002/jsc.2562.
19. Sielemann K, Hafner A, Pucker B. The reuse of public datasets in the life sciences: potential risks and rewards. PeerJ. 2020;8:e9954. doi:10.7717/peerj.9954.
20. Guillot P, Bøgsted M, Vesteghem C. FAIR sharing of health data: a systematic review of applicable solutions. Health Technol. 2023;13:869–882. doi:10.1007/s12553-023-00789-5.

21. Lalova-Spinks T, Meszaros J, Huys I. The application of data altruism in clinical research through empirical and legal analysis lenses. Front Med (Lausanne). 2023;10:1141685. doi:10.3389/fmed.2023.1141685.
22. Shabani M. The Data Governance Act and the EU's move towards facilitating data sharing. Mol Syst Biol. 2021;17(3):e10229. doi:10.15252/msb.202110229.
23. Vlahou A, Hallinan D, Apweiler R, Argiles A, Beige J, Benigni A, et al. Data Sharing Under the General Data Protection Regulation: Time to Harmonize Law and Research Ethics?. Hypertension. 2021;77(4):1029-1035. doi:10.1161/HYPERTENSIONAHA.120.16340.
24. Raab R, Küderle A, Zakreuskaya A, Stern AD, Klucken J, Kaissis G, et al. Federated electronic health records for the European Health Data Space. Lancet Digit Health. 2023;5(11):e840-e847. doi:10.1016/S2589-7500(23)00156-5.
25. Tedersoo L, Küngas R, Oras E, Köster K, Eenmaa H, Leijen Ä, et al. Data sharing practices and data availability upon request differ across scientific disciplines. Sci Data. 2021;8(1):192. doi:10.1038/s41597-021-00981-0.
26. Aleixandre-Benavent R, Vidal-Infer A, Alonso-Arroyo A, Peset F, Ferrer Sapena A. Research Data Sharing in Spain: Exploring Determinants, Practices, and Perceptions. Data. 2020;5(2):29. doi:10.3390/data5020029.
27. World Bank. Open data for sustainable development. Policy Note ICT01. August 2015. World Bank. 2015. Retrieved from https://thedocs.worldbank.org/en/doc/741081441230716917-0190022015/original/OpenDataforSustainabledevelopmentPNFINALONLINESeptember1.pdf. Accessed April, 10, 2025.
28. Smith AK, Ayanian JZ, Covinsky KE, Landon BE, McCarthy EP, Wee CC, et al. Conducting high-value secondary dataset analysis: an introductory guide and resources. J Gen Intern Med. 2011;26(8):920-929. doi:10.1007/s11606-010-1621-5.
29. Trifan A, Oliveira JL. Patient data discovery platforms as enablers of biomedical and translational research: A systematic review. J Biomed Inform. 2019;93:103154. doi:10.1016/j.jbi.2019.103154.

30. Castelo S, Rampin R, Santos A, Bessa A, Chirigati F, Freire J. Auctus: A Dataset Search Engine for Data Discovery and Augmentation. VLDB. 2021;14(12):2791-2794. doi:10.14778/3476311.3476346.

31. Chapman A, Simperl E, Koesten L, Konstantinidis G, Ibanez LD, Kacprzak E, et al. Dataset search: a survey. VLDB J. 2019;29(1):251-272. doi:10.1007/s00778-019-00564-x.

32. Krämer T, Klas CP, Hausstein B. A data discovery index for the social sciences. Sci Data. 2018;5:180064. doi:10.1038/sdata.2018.64.

33. Inau ET, Sack J, Waltemath D, Zeleke AA. Initiatives, Concepts, and Implementation Practices of the Findable, Accessible, Interoperable, and Reusable Data Principles in Health Data Stewardship: Scoping Review. J Med Internet Res. 2023;25:e45013. doi:10.1093/10.2196/45013.

34. Gregory K, Groth P, Scharnhorst A, Wyatt S. Lost or found? Discovering data needed for research. Harvard Data Sci Rev. 2020;2. doi:10.1162/99608f92.e38165eb.

35. Gusenbauer M. Search where you will find most: Comparing the disciplinary coverage of 56 bibliographic databases. Scientometrics. 2022;127(5):2683-2745. doi:10.1007/s11192-022-04289-7.

36. Page MJ, McKenzie JE, Bossuyt PM, Boutron I, Hoffmann TC, Mulrow CD, et al. The PRISMA 2020 statement: an updated guideline for reporting systematic reviews. BMJ. 2021;372:n71. doi:10.1136/bmj.n71.

37. Schmitt CP, Stingone JA, Rajasekar A, Cui Y, Du X, Duncan C, Heacock M, et al. A roadmap to advance exposomics through federation of data. Exposome. 2023;3(1):osad010. doi:10.1093/exposome/osad010.

38. Blumberg KL, Ponsero AJ, Bomhoff M, Wood-Charlson EM, DeLong EF, Hurwitz BL. Ontology-Enriched Specifications Enabling Findable, Accessible, Interoperable, and Reusable Marine Metagenomic Datasets in Cyberinfrastructure Systems. Front Microbiol. 2021;12:765268. doi:10.3389/fmicb.2021.765268.

39. Soiland-Reyes S, Sefton P, Castro LJ, Coppens F, Garijo D, Leo S, et al. Creating lightweight FAIR Digital Objects with RO-Crate. Res Ideas Outcomes, 2022;8:e93937. doi:10.3897/rio.8.e93937.
40. Perez-Riverol Y, Bai M, da Veiga Leprevost F, Squizzato S, Park YM, Haug K, et al. Discovering and linking public omics data sets using the Omics Discovery Index. Nat Biotechnol. 2017;35(5):406-409. doi:10.1038/nbt.3790.
41. Poline JB, Kennedy DN, Sommer FT, Ascoli GA, Van Essen DC, Ferguson AR, et al. Is Neuroscience FAIR? A Call for Collaborative Standardisation of Neuroscience Data. Neuroinformatics. 2022;20(2):507-512. doi:10.1007/s12021-021-09557-0.
42. Gregory K. A dataset describing data discovery and reuse practices in research. Sci Data. 2020;7(1):232. doi:10.1038/s41597-020-0569-5.
43. Wickham RJ. Secondary Analysis Research. J Adv Pract Oncol. 2019;10(4):395-400. doi:10.6004/jadpro.2019.10.4.7.
44. Tsueng G, Cano MAA, Bento J, Czech C, Kang M, Pache L, et al. Developing a standardized but extendable framework to increase the findability of infectious disease datasets. Sci Data. 2023;10(1):99. doi:10.1038/s41597-023-01968-9.
45. Dempsey W, Foster I, Fraser S, Kesselman C. Sharing Begins at Home: How Continuous and Ubiquitous FAIRness Can Enhance Research Productivity and Data Reuse. Harv Data Sci Rev. 2022;4(3):10.1162/99608f92.44d21b86. doi:10.1162/99608f92.44d21b86.
46. Kogevinas M, Schlünssen V, Mehlum IS, Turner MC. The OMEGA-NET International Inventory of Occupational Cohorts. Ann Work Expo Health. 2020;64(6):565-568. doi:10.1093/annweh/wxaa039.
47. Peters S. Although a valuable method in occupational epidemiology, job-exposure -matrices are no magic fix. Scand J Work Environ Health. 2020;46(3):231-234. doi:10.5271/sjweh.3894.
48. Gul F, Herrema H, Davids M, Keating C, Nasir A, Ijaz UZ, Javed S. Gut microbial ecology and exposome of a healthy Pakistani cohort. Gut Pathog. 2024;16(1):5. doi:10.1186/s13099-024-00596-x.

49. Patel CJ, Pho N, McDuffie M, Easton-Marks J, Kothari C, Kohane IS, et al. A database of human exposomes and phenomes from the US National Health and Nutrition Examination Survey. Sci Data. 2016;3:160096. doi:10.1038/sdata.2016.96.
50. Dimitrova M, Meyer R, Buttigieg PL, Georgiev T, Zhelezov G, Demirov S, et al. A streamlined workflow for conversion, peer review, and publication of genomics metadata as omics data papers. Gigascience. 2021;10(5):giab034. doi:10.1093/gigascience/giab034.
51. Suhr B, Dungl J, Stocker A. Search, reuse and sharing of research data in materials science and engineering-A qualitative interview study. PLoS One. 2020;15(9):e0239216. doi:10.1371/journal.pone.0239216.
52. Peng G, Gross WS, Edmunds R. Crosswalks among stewardship maturity assessment approaches promoting trustworthy FAIR data and repositories. Sci Data. 2022;9(1):576. doi:10.1038/s41597-022-01683-x.
53. Pampel H, Weisweiler NL, Strecker D, Witt M, Vierkant P, Elger K, et al. re3data - Indexing the Global Research Data Repository Landscape Since 2012. Sci Data. 2023;10(1):571. doi:10.1038/s41597-023-02462-y.
54. Doolan DM, Froelicher ES. Using an existing data set to answer new research questions: a methodological review. Res Theory Nurs Pract. 2009;23(3):203-215. doi:10.1891/1541-6577.23.3.203.
55. Petit P. Toxicological and Exposure Database Inventory: A review. Int J Hyg Environ Health. 2022;246:114055. doi:10.1016/j.ijheh.2022.114055.
56. Borgman CL, Darch PT, Sands AE, Wallis JC, Traweek S. The Ups and Downs of Knowledge Infrastructures in Science: Implications for Data Management. Proceedings of the IEEE/ACM Joint Conference on Digital Libraries. 2014. doi:10.1109/JCDL.2014.6970177.
57. Barroga E, Matanguihan GJ. A Practical Guide to Writing Quantitative and Qualitative Research Questions and Hypotheses in Scholarly Articles. J Korean Med Sci. 2022;37(16):e121. doi:10.3346/jkms.2022.37.e121.

58. Joanna Briggs Institute. Joanna Briggs Institute Reviewers' manual 2015: methodology for JBI scoping reviews. 2015. Retrieved from https://reben.com.br/revista/wp-content/uploads/2020/10/Scoping.pdf. Accessed April 3, 2025.

59. Bramer WM, de Jonge GB, Rethlefsen ML, Mast F, Kleijnen J. A systematic approach to searching: an efficient and complete method to develop literature searches. J Med Libr Assoc. 2018;106(4):531-541. doi:10.5195/jmla.2018.283.

60. Maillard O, Bun R, Laanani M, Verga-Gérard A, Leroy T, Gault N, et al. Use of the French National Health Data System (SNDS) in pharmacoepidemiology: A systematic review in its maturation phase. Therapie. 2024;79(6):659-669. doi:10.1016/j.therap.2024.05.003.

61. Lin R, Chopra B, Lin W, Shankar S, Hulsebos M, Parameswaran AG. Rethinking dataset discovery with DataScout. arXiv. 2025;arXiv:2507.18971v1. doi:10.48550/arXiv.2507.18971.

62. Alter G, Gonzalez-Beltran A, Ohno-Machado L, Rocca-Serra P. The Data Tags Suite (DATS) model for discovering data access and use requirements. Gigascience. 2020;9(2):giz165. doi:10.1093/gigascience/giz165.

63. Sansone SA, Gonzalez-Beltran A, Rocca-Serra P, Alter G, Grethe JS, Xu H, et al. DATS, the data tag suite to enable discoverability of datasets. Sci Data. 2017;4:170059. doi:10.1038/sdata.2017.59.

64. Banzi R, Canham S, Kuchinke W, Krleza-Jeric K, Demotes-Mainard J, Ohmann C. Evaluation of repositories for sharing individual-participant data from clinical studies. Trials. 2019;20(1):169. doi:10.1186/s13063-019-3253-3.

65. Groth P, Cousijn H, Clark T, Goble C. FAIR Data Reuse - the Path through Data Citation. Data Intell. 2020;2(1-2):78-86. doi:10.1162/dint_a_00030.

66. Koesten L, Simperl E, Blount T, Kacprzak E, Tennison J. Everything you always wanted to know about a dataset: Studies in data summarization. Int J Hum-Comput St. 2020;135:102367. doi:10.1016/j.ijhcs.2019.10.004.

67. Juty N, Wimalaratne SM, Soiland-Reyes S, Kunze J, Goble CA, Clark T. Unique, Persistent, Resolvable: Identifiers as the Foundation of FAIR. Data Intell. 2020;2(1-2):30-39. doi:10.1162/dint_a_00025.

68. Carroll SR, Herczog E, Hudson M, Russell K, Stall S. Operationalizing the CARE and FAIR Principles for Indigenous data futures. Sci Data. 2021;8(1):108. doi:10.1038/s41597-021-00892-0.
69. Carroll SR, Garba I, Plevel R, Small-Rodriguez D, Hiratsuka VY, Hudson M, et al. Using Indigenous Standards to Implement the CARE Principles: Setting Expectations through Tribal Research Codes. Front Genet. 2022;13:823309. doi:https://doi.org/10.3389/fgene.2022.823309.
70. Robinson E, Buys M, Chodacki J, Garzas K, Monfort S, Nancarrow C, et al. FAIR Island: real-world examples of place-based open science. Gigascience. 2023;12:giad004. doi:10.1093/gigascience/giad004.
71. Rousidis D, Garoufallou E, Balatsoukas P, Sicilia MA. Data Quality Issues and Content Analysis for Research Data Repositories: The Case of Dryad. Let's Put Data to Use: Digital Scholarship for the Next Generation. 2014:49-58. doi:10.3233/978-1-61499-409-1-49.
72. Gong Y, Liu G, Xue Y, Li R, Meng L. A survey on dataset quality in machine learning. Inf Softw Technol. 2023;162:107268. doi:10.1016/j.infsof.2023.107268.
73. Grath-Lone LM, Jay MA, Blackburn R, Gordon E, Zylbersztejn A, Wiljaars L, et al. What makes administrative data "research-ready"? A systematic review and thematic analysis of published literature. Int J Popul Data Sci. 2022;7(1):1718. doi:10.23889/ijpds.v6i1.1718.
74. Nguyen V, Middleton LYM, Zhao N, Huang L, Verly E, Kvasnicka, et al. Cleaned NHANES 1988-2018 [dataset]. Figshare. 2025;v9. doi:10.6084/m9.figshare.21743372.v9.
75. Oosterwegel MJ, Ibi D, Portengen L, Probst-Hensch N, Tarallo S, Naccarati A, et al. Processed metabolomic data from the EXPOsOMICS Personal Exposure Monitoring study [dataset]. Environ Sci Technol. 2023;57(34). doi:10.1021/acs.est.3c03233.
76. Alvarez-Romero C, Bernabeu-Wittel M, Luis Parra-Calderón C, Rodríguez Mejías S, Martínez-García A. Desiderata for discoverability and FAIR adoption of health data hubs. J Biomed Inform. 2024;157:104700. doi:10.1016/j.jbi.2024.104700.
77. Amadi D, Kiwuwa-Muyingo S, Bhattacharjee T, Taylor A, Kiragga A, Ochola M, et al. Making Metadata Machine-Readable as the First Step to Providing Findable, Accessible, Interoperable,

and Reusable Population Health Data: Framework Development and Implementation Study. Online J Public Health Inform. 2024;16:e56237. doi:10.2196/56237.

78. Leipzig J, Nüst D, Hoyt CT, Ram K, Greenberg J. The role of metadata in reproducible computational research. Patterns (N Y). 2021;2(9):100322. doi:10.1016/j.patter.2021.100322.
79. Huettemann S, Mueller RM, Dinter B. Designing ontology-based search systems for research articles. Int J Inf Manage. 2025;83:102901. doi:10.1016/j.ijinfomgt.2025.102901.
80. Belhajjame K, Zhao J, Garijo D, Hettne K, Palma R, Corcho O, et al. The Research Object Suite of Ontologies: Sharing and Exchanging Research Data and Methods on the Open Web. arXiv. 2014. doi:10.48550/arXiv.1401.4307.
81. Hauser E, Gallenmüller S, Carle G. RO-Crate for Testbeds: Automated Packaging of Experimental Results. Proceedings of the 2024 IFIP Networking Conference (IFIP Networking). 2024. doi:10.23919/IFIPNetworking62109.2024.10619057.
82. Chen X, Gururaj AE, Ozyurt B, Liu R, Soysal E, Cohen T, et al. DataMed - an open source discovery index for finding biomedical datasets. J Am Med Inform Assoc. 2018;25(3):300-308. doi:10.1093/jamia/ocx121.
83. Ohno-Machado L, Sansone SA, Alter G, Fore I, Grethe J, Xu H, et al. Finding useful data across multiple biomedical data repositories using DataMed. Nat Genet. 2017;49(6):816-819. doi:10.1038/ng.3864.
84. Davis AP, Wiegers TC, Johnson RJ, Sciaky D, Wiegers J, Mattingly CJ. Comparative Toxicogenomics Database (CTD): update 2023. Nucleic Acids Res. 2023;51(D1):D1257-D1262. doi:10.1093/nar/gkac833.
85. Zare Jeddi M, Galea KS, Viegas S, Fantke P, Louro H, Theunis J, et al. FAIR environmental and health registry (FAIREHR)- supporting the science to policy interface and life science research, development and innovation. Front Toxicol. 2023;5:1116707. doi:10.3389/ftox.2023.1116707.
86. Staunton C, Slokenberga S, Mascalzoni D. The GDPR and the research exemption: considerations on the necessary safeguards for research biobanks. Eur J Hum Genet. 2019;27(8):1159-1167. doi:10.1038/s41431-019-0386-5.

87. Christen P, Schnell R. Thirty-three myths and misconceptions about population data: from data capture and processing to linkage. Int J Popul Data Sci. 2023;8(1):2115. doi:10.23889/ijpds.v8i1.2115.

88. Anderson NR, Tarczy-Hornoch P, Bumgarner RE. On the persistence of supplementary resources in biomedical publications. BMC Bioinformatics. 2006;7:260. doi:10.1186/1471-2105-7-260.

89. Bechhofer S, Buchan I, De Roure D, Missier P, Ainsworth J, Bhagat J, et al. Why linked data is not enough for scientists. Futur Gener Comp Syst. 2013;29(2):599-611. doi:10.1016/j.future.2011.08.004.

90. Garcia-Silva A, Gomez-Perez JM, Palma R, Krystek M, Mantovani S, Foglini F, et al. Enabling FAIR Research in Earth Science through Research Objects. Futur Gener Comp Syst. 2019;98:550-564. doi:10.48550/arXiv.1809.10617.

91. Soiland-Reyes S, Sefton P, Crosas M, Castro LJ, Coppens F, Fernandez JM, et al. Packaging research artefacts with RO-Crate. Data Sci. 2022;5(2):97-138. doi:10.3233/DS-210053.

92. Custer S, Sethi T. Avoiding Data Graveyards: Insights from Data Producers & Users in Three Countries. Williamsburg, VA: AidData at William & Mary. 2017. Retrieved from https://developmentgateway.org/wp-content/uploads/2020/10/Avoiding-Data-Graveyards-Final-Report.pdf. Accessed April 10, 2025.

93. Kilgus T, Nowak A, Gersch M, Fürstenau D. Sustainability in Secondary Use of Health Data - A Scoping Review. Stud Health Technol Inform. 2024;316:398-402. doi:10.3233/SHTI240431.

94. Carragáin EO, Goble C, Sefton P, Soiland-Reyes. RO-Crate, a lightweight approach to Research Object data packaging. Proceedings of the Workshop on Research Objects 2019 (RO2019). 2019. doi:10.5281/zenodo.3337883.

95. Jacob D, Ehrenmann F, David R, Tran J, Mirande-Ney C, Chaumeil P. An ecosystem for producing and sharing metadata within the web of FAIR Data. Gigascience. 2025;14:giae111. doi:10.1093/gigascience/giae111.

96. Ainsworth J, Cunningham J, Buchan I. eLab: bringing together people, data and methods to enhance knowledge discovery in healthcare settings. Stud Health Technol Inform. 2012;175:39-48. doi:10.3233/978-1-61499-054-3-39.
97. Castro LJ, Soiland-Reyes S, Rebholz-Schuhmann. RO-Crates meets FAIR Digital Objects. Proceedings of the Conference on Research Data Infrastructure. 2023;1. doi:10.52825/CoRDI.v1i.396.
98. Leo S, Crusoe MR, Rodríguez-Navas L, Sirvent R, Kanitz A, De Geest P, et al. Recording provenance of workflow runs with RO-Crate. PLoS One. 2024;19(9):e0309210. doi:10.1371/journal.pone.0309210.
99. Sirvent R, Conejero J, Lordan F, Ejarque J, Rodriguez-Navas L, Fernandez JM. Automatic, Efficient and Scalable Provenance Registration for FAIR HPC Workflows. Proceedings of the 2022 IEEE/ACM Workshop on Workflows in Support of Large-Scale Science (WORKS). 2023. doi:10.1109/WORKS56498.2022.00006.
100. Chard K, D'Arcy M, Heavner B, Foster I, Kesselman C, Madduri R. I'll Take That to Go: Big Data Bags and Minimal Identifiers for Exchange of Large, Complex Datasets. Proceedings of the 2016 IEEE International Conference on Big Data (Big Data). 2016. doi:10.1109/BigData.2016.7840618.
101. Hardisty A, Brack P, Goble C, Livermore L, Scott B, Groom Q, et al. The Specimen Data Refinery: A Canonical Workflow Framework and FAIR Digital Object Approach to Speeding up Digital Mobilisation of Natural History Collections. Data Intell. 2022;4(2):320-341. doi:10.1162/dint_a_00134.
102. Khan FZ, Soiland-Reyes S, Sinnott RO, Lonie A, Goble C, Crusoe MR. Sharing interoperable workflow provenance: A review of best practices and their practical application in CWLProv. Gigascience. 2019;8(11):giz095. doi:10.1093/gigascience/giz095.
103. González E, Benítez A, Garijo D. FAIROs: Towards FAIR Assessment in Research Objects. 2022. In: Silvello G et al. Linking Theory and Practice of Digital Libraries. TPDL 2022. Lecture Notes in Computer Science, vol 13541. Springer, Cham. doi:10.1007/978-3-031-16802-4_6.

104. Niehues A, de Visser C, Hagenbeek FA, Kulkarni P, Pool R, Karu N, et al. A multi-omics data analysis workflow packaged as a FAIR Digital Object. Gigascience. 2024;13:giad115. doi:10.1093/gigascience/giad115.

105. Engstfeld AK, Hermann JM, Hörmann NG, Rüth J. A Lightweight File System Based Approach to Getting Data Ready for Data Management Solutions. Data Sci J. 2025;24. doi:10.5334/dsj-2025-013.

106. Wittenburg P, Hardisty A, Le Franc Y, Mozaffari A, Peer L, Skvortsov NA, et al. Canonical Workflows to Make Data FAIR. Data Intell. 2022;4(2):286-305. doi:10.1162/dint_a_00132.

107. Bayer JM, Scully RA, Dlabola EK, Courtwright JL, Hirsch CL, Hockman-Wert D, et al. Sharing FAIR monitoring program data improves discoverability and reuse. Environ Monit Assess. 2023;195(10):1141. doi:10.1007/s10661-023-11788-4.

108. Welter D, Rocca-Serra P, Grouès V, Sallam N, Ancien F, Shabani A, et al. The Translational Data Catalog - discoverable biomedical datasets. Sci Data. 2023;10(1):470. doi:10.1093/10.1038/s41597-023-02258-0.

109. Holub P, Kohlmayer F, Prasser F, Mayrhofer MT, Schlünder I, Martin GM, et al. Enhancing Reuse of Data and Biological Material in Medical Research: From FAIR to FAIR-Health. Biopreserv Biobank. 2018;16(2):97-105. doi:10.1089/bio.2017.0110.

110. Basu A, Warzel D, Eftekhari A, Kirby JS, Freymann J, Knable J, et al. Call for Data Standardization: Lessons Learned and Recommendations in an Imaging Study. JCO Clin Cancer Inform. 2019;3:1-11. doi:10.1200/CCI.19.00056.

111. Pan H, Bakalov V, Cox L, Engle ML, Erickson SW, Feolo M, et al. Identifying Datasets for Cross-Study Analysis in dbGaP using PhenX. Sci Data. 2022;9(1):532. doi:10.1038/s41597-022-01660-4.

112. Habermann T. Metadata and Reuse: Antidotes to Information Entropy. Patterns (N Y). 2020;1(1):100004. doi:10.1016/j.patter.2020.100004.

113. Gonzalez-Beltran AN, Masuzzo P, Ampe C, Bakker GJ, Besson S, Eibl RH, et al. Community standards for open cell migration data. Gigascience. 2020;9(5):giaa041. doi:10.1093/gigascience/giaa041.

114. Schröder M, Staehlke S, Groth P, Nebe JB, Spors S, Krüger F. Structure-based knowledge acquisition from electronic lab notebooks for research data provenance documentation. J Biomed Semantics. 2022;13(1):4. doi:10.1186/s13326-021-00257-x.

115. Wagner MM, Hogan WR, Levander JD, Diller M. Towards Machine-FAIR: Representing software and datasets to facilitate reuse and scientific discovery by machines. J Biomed Inform. 2024;154:104647. doi:10.1016/j.jbi.2024.104647.

116. Hardisty A, Saarenmaa H, Casino A, Dillen M, Gödderz K, Groom Q, et al. Conceptual design blueprint for the DiSSCo digitization infrastructure - DELIVERABLE D8.1. Res Ideas Outcomes. 2020;6:e54280. doi:10.3897/rio.6.e54280.

117. Pirmani A, De Brouwer E, Geys L, Parciak T, Moreau Y, Peeters LM. The Journey of Data Within a Global Data Sharing Initiative: A Federated 3-Layer Data Analysis Pipeline to Scale Up Multiple Sclerosis Research. JMIR Med Inform. 2023;11:e48030. doi:10.2196/48030.

118. DataGEMS. Data Discovery Platform with Generalized Exploratory, Management, and Search Capabilities. 2025. Retrieved from https://doi.org/10.3030/101188416. Accessed April 10, 2025.

119. An Q, Ying C, Zhu Y, Xu Y, Zhang M, Wang J. LEDD: Large Language Model-Empowered Data Discovery in Data Lakes. arXiv. 2025. doi:10.48550/arXiv.2502.15182.

120. Kreutz CK, Perry A, Friedrich T. Data Discovery using LLMs -- A Study of Data User Behaviour. 2025. doi:10.48550/arXiv.2507.04444.

121. Jiang S, Sørbø S, Tinn P, Karim SF, Roman D. LLMDap: LLM-based Data Profiling and Sharing. Proceedings of the VLDB 2025 Workshop: 3rd Data EConomy Workshop (DEC). 2025. Retrieved from https://www.vldb.org/2025/Workshops/VLDB-Workshops-2025/DEC/DEC25_5.pdf. Accessed September 15, 2025.

122. Zhang Y, Khan SA, Mahmud A, Yang H, Lavin A, Levin M, et al. Exploring the role of large language models in the scientific method: from hypothesis to discovery. npj Artificial Intelligence. 2025;1:14. doi:10.1038/s44387-025-00019-5.

123. Zheng T, Deng Z, Tsang HT, Wang W, Bai J, Wang Z, et al. From Automation to Autonomy: A Survey on Large Language Models in Scientific Discovery. arXiv. 2025. doi:10.48550/arXiv.2505.13259.

124. Marini P, Santos A, Contaxis N, Freire J. Proceedings of the Fifth Workshop on Scholarly Document Processing (SDP 2025). 2025;114-123. doi:10.18653/v1/2025.sdp-1.10.

Figure 1

Click here to access/download;Figure;Figure_1.pdf

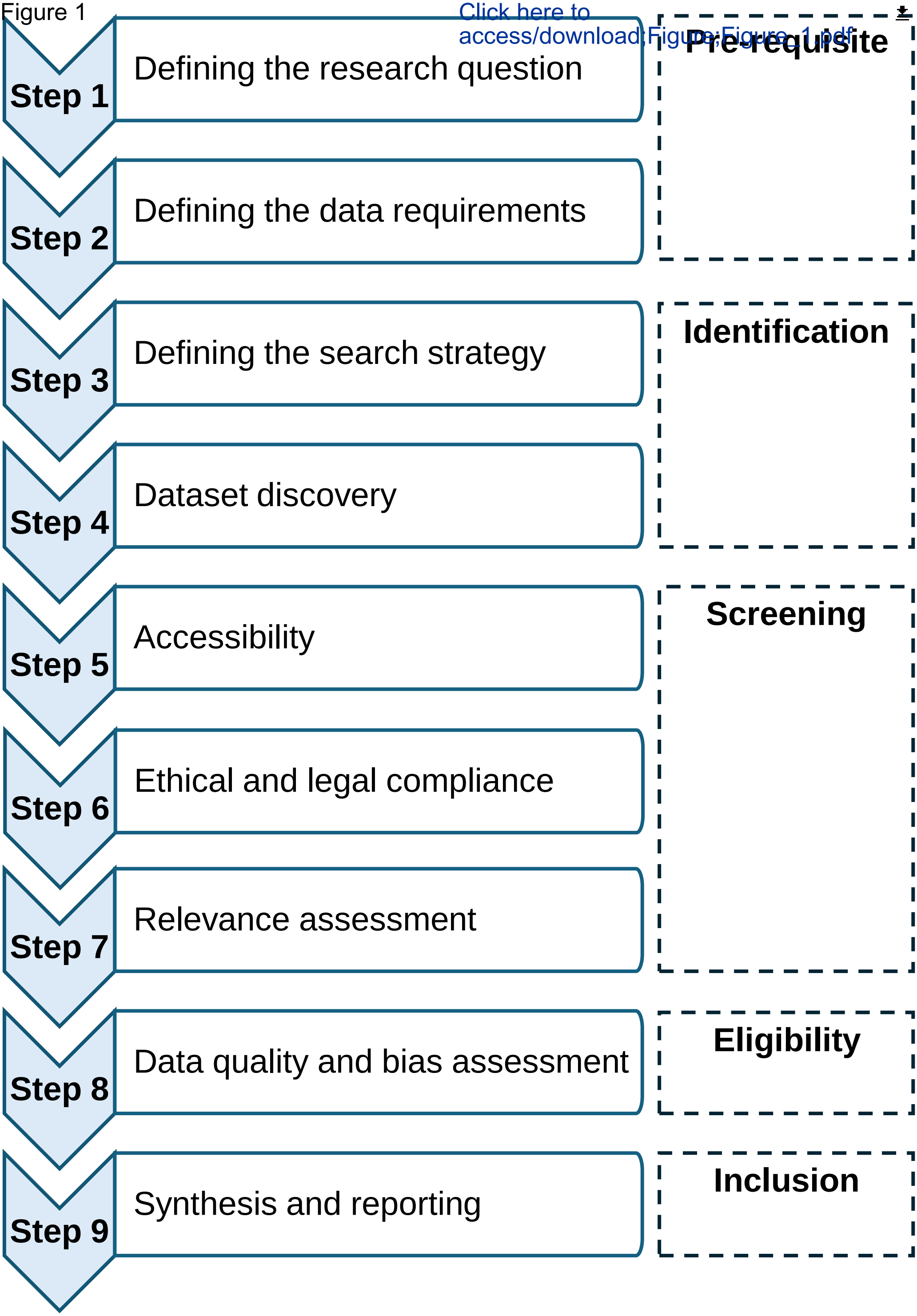

Click here to access/download;Figure;Figure_3.pdf

Figure 3

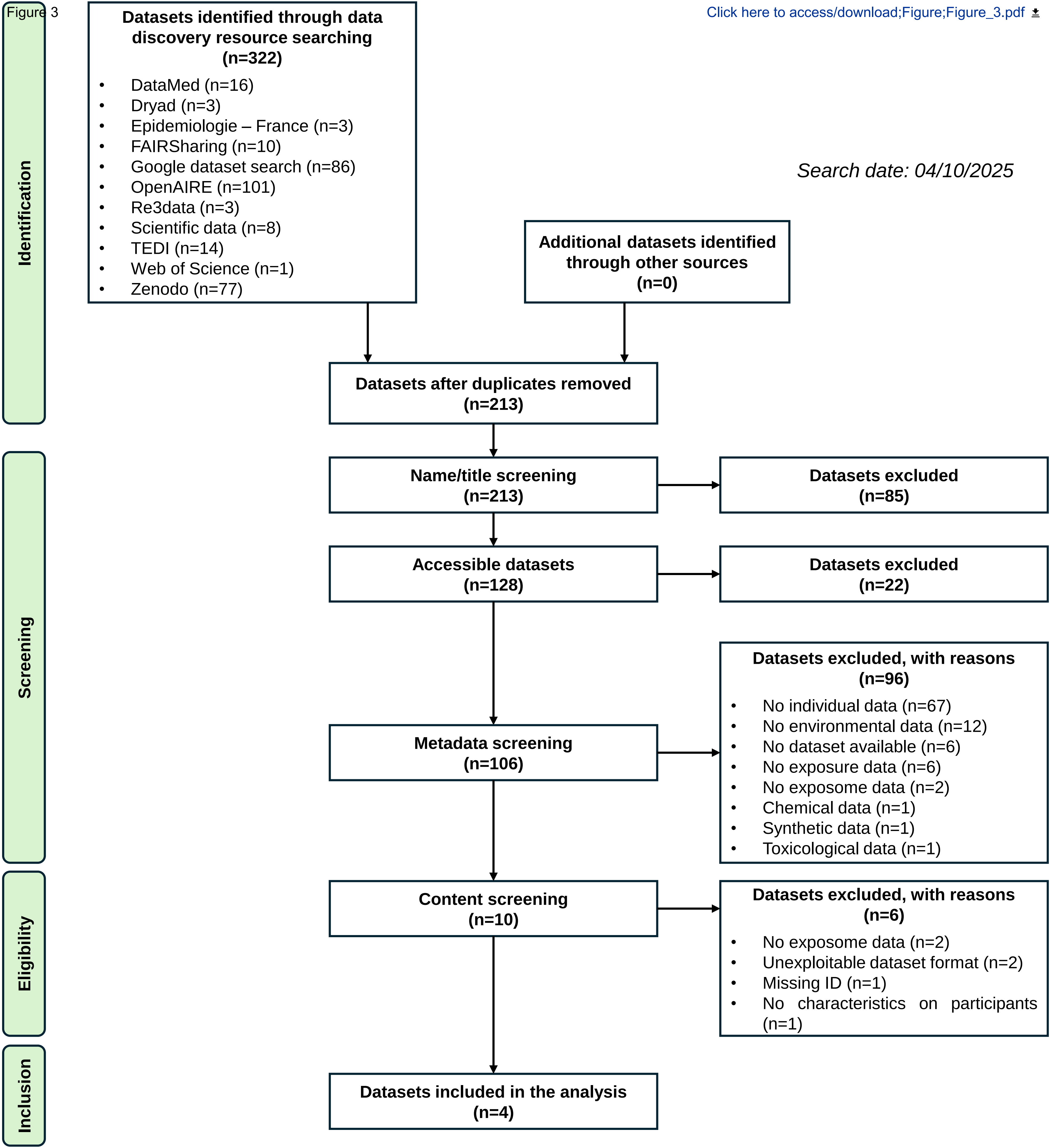

Figure 2

Click here to access/download;Figure;Figure_2.pdf

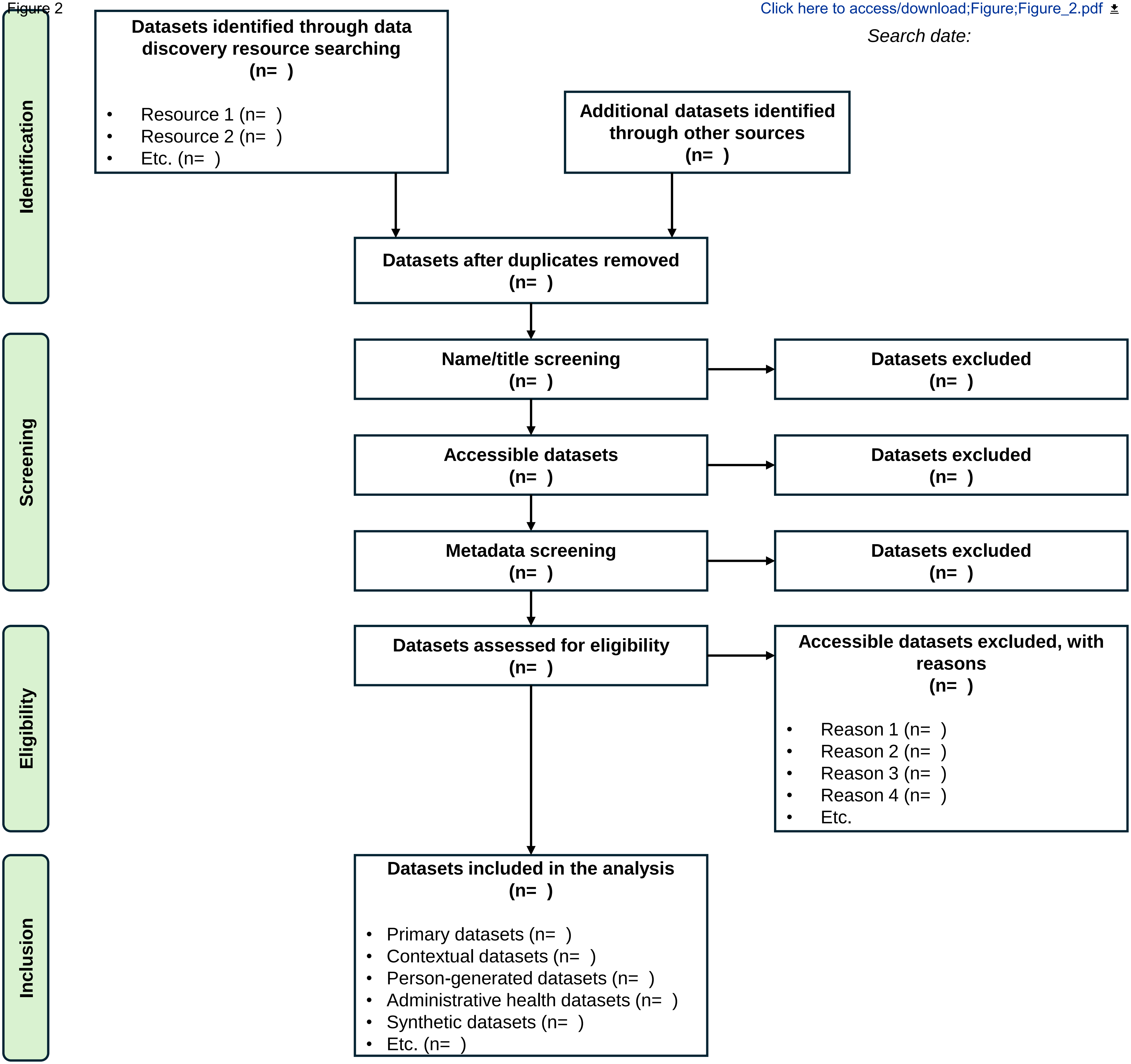

Graphical Abstract

Datagraphy: toward a systematic approach to dataset discovery

Click here to access/download;Graphical

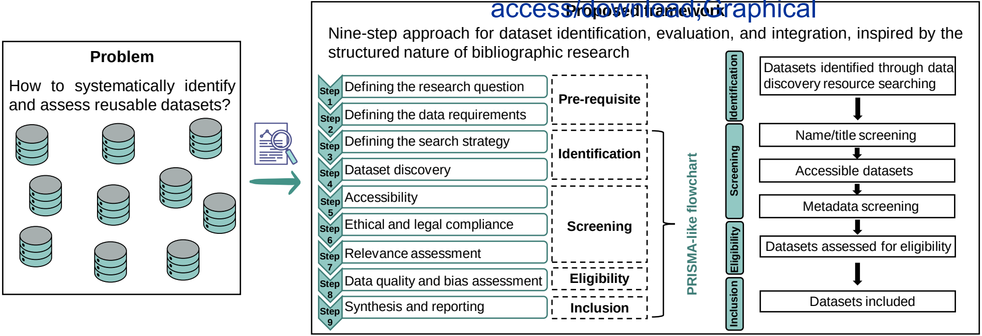

Supplement: giaf134_GIGA-D-25-00204_Revision_1 [file giaf134_giga-d-25-00204_revision_1.pdf]
