# Supplementary material for: Datagraphy: toward a systematic approach to dataset discovery
Source: Gigascience. 2025 Oct 22;14:giaf134. doi: 10.1093/gigascience/giaf134 (PMC12644982; doi:10.1093/gigascience/giaf134)
Supplement: giaf134_Authors_Response_To_Reviewer_Comments_Original_Submission [file giaf134_authors_response_to_reviewer_comments_original_submission.pdf]

Author's Response To Reviewer Comments

Rebuttal letter

Dear Editor,

Please find attached the revised version of the manuscript number GIGA-D-25-00204 entitled “Datagraphy: toward a systematic approach to dataset discovery,” which we are re-submitting for publication as an article in GigaScience.

We would like to warmly thank the reviewers for their constructive comments and suggestions that have been carefully taken into account in reworking this new version of the manuscript. The responses to reviewer questions and all changes made in the manuscript are listed below. A marked-up version of the manuscript with the changes to the text in red, blue, and/or green fonts was made.

Now, we hope that this revised version will be suitable for publication. In all cases, we remain open to any suggestions.

Thank you for considering our work.

Sincerely,
Pascal Petit, on behalf of all authors
Point by point response to reviewers

Reviewer #1
Dear Authors,

The concept that was proposed in this manuscript, datagraphy, makes it clear that there are real problems with the way current datasets are published through "generic" data repositories. The proposed framework shows a real and growing problem regarding inefficient and inconsistent dataset discovery. This problem however is in this approach tackled or perhaps bandaged by proposing a structured method toward the researcher. Although the nine steps mentioned, it remains an intensive exercise and when in a popular field would need to be done frequently to enable the discovery of new datasets.

This proposed framework however does not propose any approach to tackle the real problems. Which is the freedom data repositories have to implement their own procedures and methods for data discovery. Without this focus this sounds more like a bandaid than a solution towards the problem that is being tried to address here. In addition researchers that search for data often also deposit datasets. It might be a more, in the long term, solution if standards are proposed that aid in the discovery of datasets through standardised metadata templates that are submitted alongside the datasets such as in the form of Research Objects or RO-crates as they are essentially a lightweight approach to packaging research data with their metadata.

Overall I think the approach has merit but it does not tackle the real issue at hand.

Authors: Thank you for your review and your relevant comments. We value your time and effort.

We thank you for this valuable comment, which highlights an important limitation of our current framework and points toward relevant future directions. We fully agree that the challenges of dataset discovery cannot be resolved solely at the level of individual researchers. As you rightly note, the main root cause lies in the freedom that repositories have in implementing their own procedures, standards, and metadata requirements.
Our proposed framework for datagraphy, however, is not intended to replace systemic solutions but rather to provide researchers with a structured and transparent methodology for navigating the current

heterogeneous ecosystem. In this sense, datagraphy can be seen as a pragmatic approach designed to improve reproducibility and rigor under the existing conditions, while broader systemic changes are still under development.
We also agree that repository-level interventions, such as the adoption of standardized metadata templates, are indispensable to long-term progress. Your suggestion to consider Research Objects (ROs) and RO-Crates is thus particularly relevant. These initiatives provide a lightweight but powerful mechanism for packaging data together with machine-readable metadata, thereby enhancing discoverability and reusability.
We are grateful for this comment, which has helped us refine the positioning of our contribution. Our revision will therefore make clearer that while datagraphy improves transparency and reproducibility in the short term, it must evolve in tandem with repository-level initiatives that standardize metadata and enhance the FAIRness of data resources.

We have revised the manuscript to explicitly acknowledge the importance of such systemic solutions and to position datagraphy as complementary to ongoing standardization efforts. In particular, we have clarified in the discussion subsection that datagraphy is currently a "researcher-centric" framework designed to work within present limitations, while systemic and community-driven efforts are needed to address the root problem. We have also highlighted that datagraphy and community efforts should be pursued together. Datagraphy can provide the methodology for systematic dataset identification and evaluation, whereas systemic solutions (e.g., metadata standardization) will improve the underlying infrastructure for discovery.
Please refer to lines 33-43 in the revised version of the manuscript with track changes (or to lines 33-41 in the revised version without track changes): "We discuss major challenges faced by datagraphy with respect to metadata availability, repository heterogeneity, dataset accessibility, and dataset quality, and highlight how datagraphy could enhance transparency, reproducibility, and efficiency at the researcher level. Datagraphy is intended to complement repository-level improvements. Aligning researcher practices with standardized, machine-readable metadata, persistent identifiers, artificial intelligence integration, and lightweight packaging frameworks such as RO-Crates and FAIR Digital Objects could enable automated discovery and sustainable dataset reuse. By integrating structured researcher-level methodology with systemic improvements and community efforts, datagraphy could offer a scalable approach for systematic, FAIR-aligned, data-driven research across disciplines".
Please also refer to lines 185-190 in the revised version of the manuscript with track changes (or to lines 178-183 in the revised version without track changes): "At the same time, it must be acknowledged that this framework operates within the limitations of existing repository practices and infrastructures, which remain heterogeneous and largely unstandardized. Thus, the proposed methodology should be understood as a pragmatic, researcher-centric approach that enhances efficiency and rigor in dataset discovery, while recognizing that systemic repository-level improvements are still necessary to fully address the underlying challenges.".
Please also refer to lines 380-725 in the revised version of the manuscript with track changes (or to lines 369-652 in the revised version without track changes): "6. Discussion
6.1 Challenges
The successful implementation of datagraphy faces multiple challenges.

6.1.1 Metadata availability, heterogeneity, and quality
One of the primary obstacles is the limited availability of metadata, which is essential for assessing the existence and characteristics of datasets. Current deposition practices impose only minimal restrictions on the descriptors used [17]. The substantial variability in metadata and the lack of standardized protocols for data collection, storage, and format/structure across repositories create major barriers to the effective identification and reuse of datasets. While datagraphy could help researchers navigate this fragmented landscape more systematically, it does not resolve the underlying heterogeneity. In the absence of consensus on core repository services, each organization tends to implement systems aligned with its specific goals [64], making cross-repository integration challenging. For example, in data papers and data-focused journals, publication requirements and the amount of data that can be attached vary by journal, making it challenging to find comprehensive and useful content. In addition, limited accessibility and machine-readability of existing metadata, in particular for population health data, further hamper effective dataset discovery and reuse [76-78].
Metadata quality represents a further critical bottleneck. Because dataset search relies heavily on

metadata, it is imperative that descriptions are accurate and consistent [31]. Metadata are essential for establishing dataset existence, scope, and suitability for reuse, yet they are frequently incomplete, restricted to high-level descriptors, inconsistent, or non-standardized [20,30,31]. Metadata quality also varies across disciplines and repositories, reflecting differences in cultural practices and curation priorities [71]. Errors, inconsistencies, and incompleteness in metadata are common, sometimes diverging from the underlying dataset [30]. Consequently, relying solely on metadata constrains discoverability and hampers systematic evaluation of dataset fitness for purpose [30,31].

6.1.2 Data discovery infrastructure and landscape

One major issue for dataset discovery is the broad freedom repositories have to implement their own discovery procedures, metadata standards, and data packaging approaches [2,64]. This heterogeneity creates structural challenges that go beyond the capacity of individual researchers to address. While existing resources accept a wide variety of data types and formats, they generally do not attempt to integrate, harmonize, or assess the quality of deposited data. Datasets may be incomplete, sometimes intentionally so [25]. Complex datasets, such as collections of tables, longitudinal records, or multi-modal data, remain poorly supported [45]. Certain dataset types, including administrative health records, lack comprehensive catalogs and are underrepresented in existing data repositories [6]. Searchable dataset discovery resources, whether general repositories or domain-specific catalogs, also differ in functionality and coverage, including subject matter, geographical and temporal scope, language, and scientific domain [2,35]. Consequently, the data ecosystem is becoming increasingly fragmented and heterogeneous, creating a “needle in a haystack” paradox that further complicates dataset discovery [17], in particular in domains where data lakes may contain tens of thousands of tables [61].

Dataset search is largely keyword-based, restrictive, and iterative, often requiring multiple query reformulations to assess relevance [31,61]. Such keyword-based approaches, which rely on limited metadata, are insufficient for comprehensive dataset search and discovery [11,30,31]. They fail to capture dataset granularity, provenance, and methodological context [31,61]. Many repositories only support simple keyword-based queries, which limits users’ ability to express complex information needs [30]. Although some tools allow searches for spreadsheets or published data in formats such as CSV or JSON, most do not support complex datasets, such as collections of tables, text, or temporal data [11,31]. In addition, existing searchable data discovery resources often exhibit limited data discovery capabilities and provide only superficial query responses [11,31,61]. Translating search strategies and user queries across searchable dataset resources with differing syntax requirements can be a complex and time-consuming task [59]. To address this issue, the adoption of standardized vocabularies (e.g., taxonomies tailored to domain-specific terminology) is essential. One promising direction is the development of web-based search systems leveraging semantic metadata, as recently proposed in the field of information science for literature searches of scientific publications [79]. Current standards such as Wikidata, Schema.org, and Dublin Core provide structured vocabularies for describing digital resources in a consistent, machine-readable way, while domain-specific ontologies enable richer semantic annotation [31,32,44,65,80,81]. There is a growing need for richer, content-aware indexing and semantic search methods [11,30,61]. Several initiatives have developed dataset discovery indexes (DDIs) and search engines to mitigate repository heterogeneity and search limitations. Examples include DataMed, designed as a “PubMed for datasets” [62,63,82,83]; OmicsDI, which aggregates resources across proteomics, genomics, and metabolomics [40]; Auctus, a web crawler and search engine dedicated to datasets for data augmentation in machine learning [30]; and Google Dataset Search, which indexes a broad range of datasets published on the web [30,31]. These search engines attempt to index heterogeneous repositories by exposing standardized metadata fields. Most repositories, however, still support only simple searches over metadata [30], although some, such as Auctus, enable more complex queries incorporating spatial, temporal, and integration constraints based on dataset content [30]. While these approaches demonstrate the feasibility of federated search, they remain constrained by the heterogeneity and incompleteness of the underlying metadata [11,31]. Advanced approaches, including semantic search, artificial intelligence (AI)-assisted query reformulation [61], and content-aware indexing, offer potential improvements [11,30,61]. Nevertheless, search effectiveness continues to be limited primarily by metadata heterogeneity and repository-specific practices [11,31].

6.1.3 Data accessibility, legal, and ethical considerations

Challenges also extend to repository functionality and scope. Dataset documentation and access conditions vary tremendously across resources, making selection difficult for researchers. Dataset accessibility is

further complicated by licensing, ethical, and legal constraints. Licenses, data-use agreements (DUAs), and authentication requirements introduce additional barriers and shape who can access datasets and under what conditions [62]. Open-access datasets may be freely available, but many sensitive datasets require IRB approval, registered access, or multifactor authentication, adding layers of complexity and potential delays [60,62]. The time required to acquire existing datasets and obtain IRB approval can also vary considerably [28]. While some datasets can be directly downloaded from the web (e.g., Comparative Toxicogenomics Database [84]), others require multiple layers of permissions and security measures (e.g., SNDS [60]), and in certain cases, data must be analyzed within a dedicated data processing environment (e.g., SNDS [60]). These examples illustrate the wide diversity of access conditions researchers must navigate. Furthermore, national and international ethical and legal obligations can restrict data sharing, grouped analyses, and data deposition [25,85]. Regulations such as the European GDPR require organizations to implement robust data protection measures, including data retention and deletion protocols, which can impede data reuse efforts and may result in substantial financial penalties in case of non-compliance [23,86].

Finally, access to published data is not always guaranteed due to broken links, missing metadata, or a lack of author cooperation [25]. For example, in a large-scale study of around 900 articles published in Nature and Science between 2000 and 2019, 61% of papers that included “data available upon request” statements did not provide the data when contacted [25]. Broken links, missing metadata, or author unresponsiveness therefore remain major obstacles.

6.1.4 Data quality and reusability

Researchers frequently encounter poorly described and non-standardized data, which limits reusability [20]. Assessing dataset fitness is often difficult, as available metadata rarely capture essential attributes such as granularity, provenance, or methodological context [31,61]. As a result, substantial time and effort are often required to manually sift through large volumes of irrelevant datasets.

Another major challenge in assessing data quality and reusability lies in the original data collection methods and processes (i.e., data capture), which are beyond the control of secondary users and can highly vary [72,87]. In addition to variability in metadata completeness and repository practices, the lack of standardized indicators or score systems for assessing dataset reusability (e.g., FAIRness) and quality further complicates efforts to identify the most appropriate datasets for specific research questions [2,19,76]. Practical considerations such as transit costs, storage, and access proximity may also influence dataset reusability [67].

Sharing data via supplementary materials remains common practice and is highly encouraged by journals [88]. However, unlike public repositories, supplementary files are not necessarily persistently discoverable or archived. They frequently lack persistent identifiers (e.g., DOIs or accession numbers), making them vulnerable to link rot and content drift [88]. Moreover, supplementary datasets are often available only in aggregated or summary form, rather than as raw or individual-level data, which limits systematic reuse. Publishing datasets solely as linked data or supplementary materials is insufficient to support reproducibility and reuse, as such approaches fail to capture lifecycle information, provenance, versioning, and methodological context [89-91].

6.1.5 Data visibility, data decay, and sustainability

Systemic challenges such as dataset invisibility, data decay, broken links, and paywalls exacerbate discovery difficulties [25]. Some datasets remain inaccessible behind paywalls, making them invisible to many researchers [35], while others are never deposited in open repositories, resulting in so-called invisible data or dark data [14] and increasing the risk of data graveyards (i.e., unused data) [92]. Organizations frequently store vast numbers of tables and records in data lakes [61]. Over time, the availability of originally accessible data tends to decline (a phenomenon known as data decay), which can ultimately lead to data loss [25]. Estimates suggest that up to 80% of archived scientific research data are lost within 20 years [67].

Sustainability poses an additional systemic risk. Many data-sharing platforms lack long-term funding models, which threatens not only the durability and accessibility of datasets but also the continued relevance of the information they host [93]. Without sustained funding and community support, data search tools risk becoming outdated or abandoned, which compromises their reliability and long-term utility for researchers. For example, DataMed has not received new additions since 2024 [62,63,82,83]. Some repositories risk becoming unsustainable “data lakes,” where uncurated, poorly described data accumulate without integration or retrieval mechanisms [35]. Without investment in long-term

infrastructure, persistent identifiers, harmonized metadata standards, and robust governance structures, repository heterogeneity, metadata gaps, and fragmentation will persist [56]. In the absence of such measures, both discoverability and reuse remain severely undermined.

6.1.6 Standardized metadata frameworks and packaging

Efforts to improve data discoverability across the social sciences, biomedical domains, omics research, and public datasets emphasize the need for metadata harmonization, advanced search interfaces, and distributed resource indexing [32,40]. Implementing repository-wide FAIR-compliant practices and semantic metadata frameworks could help address existing limitations [38,39,81,94,95]. While the FAIR principles provide an overarching vision for dataset reuse, their adoption at the repository level remains highly uneven [20,64]. Two recent reviews, covering 35 [20] and 25 [64] data repositories, respectively, identified interoperability and sustainability as major obstacles to achieving FAIR compliance.

Repositories differ in the metadata standards they apply, the search interfaces they provide, and the approaches they adopt for data packaging. This diversity produces structural fragmentation that cannot be addressed at the level of individual researchers [2,64]. Most attempts to mitigate these limitations have focused on standardized, machine-readable metadata and packaging approaches. By adopting FAIR digital objects (FDOs) or research object (RO)-Crate packages, repositories could ensure that metadata, provenance, licensing, and dataset structure are consistently available, machine-actionable, and interoperable, thereby facilitating automated discovery and reuse across platforms [38,39,81,91,94-98].

ROs are structured bundles of data, methods, and metadata that capture lifecycle information, ownership, versioning, attribution, provenance, quality, and methodological context [89-91]. RO-Crate extends this concept by providing a lightweight, machine-readable packaging framework for aggregating research artifacts with their metadata and relationships, creating a multimodal scholarly knowledge graph that can help “FAIRify” and combine metadata from existing resources [91,94,97,99]. FDOs, in turn, are digital objects explicitly designed to be FAIR, embedding metadata, provenance, and other descriptive information in a machine-actionable format [38,65]. Other packaging standards, such as BDBags (Big Data Bags), provide mechanisms for content enumeration, fixity checking, and lightweight referencing without requiring centralized hosting [45,100]. The integration of canonical workflows, including pipelines based on the Common Workflow Language (CWL), could further support reproducibility and enable systematic evaluation of search queries and analyses across repositories [101,102].

Validation frameworks, reusability metrics, and machine-readable metadata derived from RO-Crates or FDOs could enable semi-automated or automated assessments of dataset fitness and quality [38,103,104]. For instance, the FAIRO framework has been proposed to measure the compliance of ROs with FAIR criteria [103], while reusability indicators and automated quality checks have been incorporated into RO and RO-Crate packaging [38,105]. However, the effectiveness of automation remains limited by the lack of harmonized metadata across repositories [65,89,106].

Additional complementary approaches could further enhance discoverability. Persistent identifiers (e.g., DOIs) can ensure long-term accessibility, prevent broken links, and support dataset citation [13,67]. The adoption of persistent identifiers is essential for datagraphy, yet remains underdeveloped [13,19]. Assigning persistent identifiers guarantees long-term accessibility, facilitates citation, and prevents link rot caused by website migrations [107]. Metadata harmonization frameworks, such as the Data Tag Suite (DATS), also enable standardized descriptions of heterogeneous datasets and support automated search and retrieval [62,63,108].

Despite these advances, the adoption of standardized packaging and metadata frameworks remains limited, constraining automation, reproducibility, and scalability in dataset discovery [65,89,106].

6.2 Potential implications and future directions

The aforementioned structural challenges underscore the necessity of harmonizing both repository-level infrastructures and researcher-level practices.

6.2.1 Datagraphy as a systematic research methodology

Establishing datagraphy as a recognized research methodology could have profound implications across scientific disciplines. By promoting systematic dataset selection, datagraphy may enhance the reliability and reproducibility of data reuse research, mitigate selection bias, facilitate transparent dataset reuse, and foster interdisciplinary collaboration through improved dataset discoverability. As scientific data grow in volume and complexity, the need for a structured approach to dataset selection becomes increasingly urgent. Integrating datagraphy as a foundational research practice would ensure that dataset selection

adheres to the same methodological rigor as literature reviews, ultimately strengthening the integrity and impact of data-driven discoveries.

6.2.2 Standardization efforts

To advance datagraphy, standardization efforts are essential, much as they have been for bibliographic search. Establishing reporting guidelines analogous to PRISMA [36] could be a viable strategy. Another option could be extending existing frameworks (e.g., adapting PRISMA for dataset discovery). Unified guidelines for cloud architectures [109], the promotion of common data elements [110,111], and the standardization of reporting formats and metadata sharing remain essential priorities [112]. Richly described metadata in machine-readable formats will enhance interoperability, enabling efficient data harvesting, attribution, and semantic understanding [77,107,110-112]. Ongoing harmonization initiatives, such as those in toxicology [85], should be further supported and expanded.

6.2.3 Aligning researcher and repository-level practices

For datagraphy to realize its full potential, it must operate in synergy with repository-level improvements. Sustainable and reproducible datagraphy requires alignment with community standards for metadata and packaging. This includes the adoption of standardized, machine-readable metadata schemas (e.g., DATS), harmonized vocabularies, persistent identifiers (e.g., DOIs), and lightweight packaging formats such as RO-Crates, BDBags, Frictionless Data Packages, or FDOs [39,62,63,80,94,95,100,105,113]. Integrating provenance-aware workflows and schema-validated metadata could further strengthen reproducibility and automation [99,114,115]. These implementations would provide the foundations for machine-actionable dataset discovery and ensure that datasets remain discoverable, comparable, and citable across repositories [39,95,106]. Repository-level innovations, including semantic search, canonical workflows, and federated queries across FAIR-compliant repositories, can further reduce manual search effort while increasing reproducibility [10,101,106,116].

6.2.4 FAIRness, reusability, and sustainability

Developing sustainable and trustworthy FAIR-compliant searchable data discovery resources (e.g., data repositories) is essential for supporting datagraphy. The creation of a comprehensive search index could enable dataset comparison [32]. However, empirical insights into what makes a dataset more reusable remain limited [2]. Guidelines such as FAIR, which promote universal metadata standards, are essential for dataset comparison and integration [19,76]. While measuring "FAIRness" is not yet an established practice, several efforts (e.g., the FAIR metrics group) are paving the way [2]. Validation frameworks such as FAIRO [103] and automated FAIRness metrics [38,105] could provide objective indicators of dataset quality. Implementing FAIR principles enhances discoverability and reuse, ensuring seamless dataset discovery, access, and integration across diverse research domains [76,77,107]. Complementing the FAIR principles with the TRUST (Transparency, Responsibility, User focus, Sustainability, and Technology) principles could provide a more comprehensive framework for data sustainability [93]. In addition, operationalizing the FAIR Principles alongside other standards, such as the CARE principles, can enhance machine actionability while also ensuring that data are used appropriately across the entire data lifecycle [68].

Integrating dataset search tools with bibliographic databases and data management platforms represents a promising step toward improving the findability of datasets [34]. For instance, databases such as Web of Science and PubMed enable users to filter search results specifically for data papers. This can be achieved via a dedicated filter box in Web of Science or by appending searches with the term data[filter] in PubMed. However, this functionality is not yet available in other bibliographic databases. In contrast, some literature repositories like PubMed Central offer more sophisticated search capabilities that allow users to limit queries to articles associated with data. This can be done by utilizing specialized search operators, including hassuppdata, hasdataavail, hasdatacitations, and hasassociateddata, which target papers containing data availability statements or supplemental data files.

6.2.5 Governance and community initiatives

Community-driven initiatives will play a central role in advancing datagraphy. Projects such as the RDA [20], DCAT [2], DATACC (https://www.datacc.org/en/), and schema.org [2] can enhance datagraphy by improving dataset discoverability and enabling federated searches across multiple data catalogs. A unified data service is needed to efficiently retrieve relevant and reliable datasets [32]. For example, Wikidata provides a community-maintained knowledge graph that supports semantic enrichment and linking across datasets [31,65]. Another example is the Global Data Sharing Initiative that proposed a pipeline that

collects relevant information from diverse sources, integrates multiple sharing streams, and merges them into a unified dataset for statistical analysis or secure data examination [117]. The European initiative DataGEMS [118] aims to address this challenge by developing an advanced data discovery platform based on FAIR principles. DataGEMS will integrate data sharing, discovery, and analysis into a comprehensive ecosystem covering the entire data lifecycle (i.e., storage, management, discovery, analysis, and reuse). This EU-funded initiative involves twelve partners across eight countries working to create open-source tools that facilitate access to FAIR-by-design datasets. By promoting data FAIRness, DataGEMS will bridge the gap between data providers and users, fostering a more efficient and transparent data-sharing ecosystem. Complementary efforts, such as Make Data Count [19], further legitimize datasets as scholarly outputs, enabling proper citation and attribution alongside traditional publications.

6.2.6 AI integration in dataset discovery
The integration of AI-based tools for automated dataset discovery could represent a promising avenue for enhancing datagraphy. For example, DataGEMS will leverage state-of-the-art data management, natural language processing, and machine learning to support dataset discovery and analysis across diverse data modalities, including tabular data, text documents, knowledge graphs, and images [118]. AI-based tools such as DataScout [61] and large language models (LLMs) promise to enhance dataset discovery through query reformulation, semantic filtering, and relevance scoring [119-124]. These innovations complement, rather than replace, necessary repository-level changes such as metadata harmonization and packaging. Together, technological innovation and metadata harmonization must evolve in concert, rather than in isolation.

6.3 Conclusion
Datagraphy and community-driven efforts should be advanced in synergy. Datagraphy provides a structured methodology for the systematic identification and evaluation of datasets, whereas systemic solutions (e.g., metadata standardization) strengthen the underlying infrastructure that supports dataset discovery. As a reproducible, pragmatic, and researcher-centered approach, datagraphy offers a valuable means of navigating the fragmented landscape of dataset discovery. However, its full effectiveness depends on addressing repository heterogeneity through the implementation of standardized, machine-readable metadata, persistent identifiers, provenance-aware workflows, and FAIR-compliant packaging formats such as RO-Crates or FDOs. By aligning datagraphy with repository-level standards and community best practices, researchers can substantially improve dataset discoverability and foster sustainable reuse. In this way, datagraphy functions both as a practical methodology and as a conceptual framework that underscores the necessity of harmonized infrastructure. When integrated with FAIR-aligned repositories and robust reporting guidelines, datagraphy has the potential to transform dataset selection into a transparent, durable, and scalable research practice, thereby enabling researchers to engage more effectively and confidently with increasingly complex data landscapes.”.

Reviewer #2

This paper addresses an important topic, and I suggest that it be published with some revisions. I have included some areas for revision below:

- Re3Data is a listing of data repositories, not a data repository itself. For example, if I search for 'cancer' in Re3Data, I will be shown data repositories that specialize in cancer, not datasets on cancer. In your example on Line 275, please clarify what the three resources you located in Re3Data and if they were datasets or data repositories. If you located a dataset by searching a repository you located through Re3Data, please clarify that as well.
Authors: Thank you for your review and your relevant comments. We value your time and effort.

You are correct that Re3Data is a catalog of data repositories rather than a data repository itself. It functions as a registry that provides information about research data repositories across disciplines. Researchers can use Re3Data to identify suitable repositories for depositing or accessing datasets, but the platform does not host data directly. To reflect this distinction more accurately and to avoid any ambiguity, we have replaced the term “data repository” with “data repository catalog” in Table 1. Please refer to Table 1.

It is true that Re3Data allows filtering by content type, including datasets (e.g., https://www.re3data.org/search?query=cancer&contentType40%5B%5D=dataset). However, to identify the three resources mentioned in our example, we did not apply the content type filter. Instead, we used the general query (https://www.re3data.org/search?query=exposom*), which yielded only three results. For transparency and reproducibility, we have added the URLs of the searches used for all 11 resources in the Supplementary Materials (see Table A.1) and expanded the description of our approach in the section "5. Illustrative and working example: the exposome case." Please refer to Table A.1 and to lines 342-346 in the revised manuscript with track changes (or to lines 331-335 in the clean version): "For five resources, filter or facet options were used, with the "catalogue" facet for Epidemiologie – France, the data paper option in Web of Science (from the document types filter option), the dataset option in FAIRSharing (from the object types filter), OpenAIRE (from the document type filter), and Zenodo (from the resource types filter). Regarding Re3Data, the content type filter to select datasets was not used because only three results were obtained with the general search query.".

- Similarly FAIRSharing is a listing of data repositories (referred to as databases on their site), data policies, and data standards. Please clarify in your example on Line 275 what 10 resources your located in FAIRSharing and if they were data repositories, data policies, or data standards. If you located a dataset by searching a repository you located through FAIRSharing, please clarify that as well.
Authors: Thank you for your relevant comment. You are correct that it provides listings of different types of resources, including data repositories (referred to as databases on the site), data policies, and data standards. Consequently, FAIRSharing is a data catalog (cf. Table 1). Importantly, FAIRSharing also allows users to identify datasets by applying the appropriate filters. For example, when searching for "exposome," one can refine the results by selecting the object type "dataset": https://fairsharing.org/search?q=exposome&objectTypes=dataset.
To clarify this point, we added a sentence in the revised version of the manuscript. Please refer to lines 342-346 in the revised manuscript with track changes (or to lines 331-335 in the clean version): "For five resources, filter or facet options were used, with the "catalogue" facet for Epidemiologie – France, the data paper option in Web of Science (from the document types filter option), the dataset option in FAIRSharing (from the object types filter), OpenAIRE (from the document type filter), and Zenodo (from the resource types filter). Regarding Re3Data, the content type filter to select datasets was not used because only three results were obtained with the general search query.".
For transparency and reproducibility, we also added the URLs of the searches used in the supplemental materials. Please refer to Table A.1.

- Missing in your overall workflow is ways to locate dataset contained in supplementary materials. PubMed Central, for example, allows users to limit searches to papers that have associated data (either in a Data Availability Statement or in the supplemental files). I understand if you decide not to include that type of search in your PRISMA-like workflow, but you should at least mention that the workflow will not locate those types of materials.
Authors: We acknowledge that our current workflow does not systematically capture datasets that are available only in the supplementary materials of publications. To address this limitation, we modified the workflow and explicitly incorporated bibliographic databases that provide such functionality. In particular, PubMed and PubMed Central were added to Table 1. Please refer to Table 1.
Please also refer to lines 258-259 in the revised version of the manuscript with track changes (or to lines 248-249 in the revised version without track changes): "It is also possible to locate datasets associated with papers in bibliographic databases, such as PubMed.".
Please also refer to lines 658-668 in the revised version of the manuscript with track changes (or to lines 601-609 in the revised version without track changes): "Integrating dataset search tools with bibliographic databases and data management platforms represents a promising step toward improving the findability of datasets [34]. For instance, databases such as Web of Science and PubMed enable users to filter search results specifically for data papers. This can be achieved via a dedicated filter box in Web of Science or by appending searches with the term data[filter] in PubMed. However, this functionality is not yet available in other bibliographic databases. In contrast, some literature repositories like PubMed Central offer more sophisticated search capabilities that allow users to limit queries to articles associated with data. This can be done by utilizing specialized search operators, including hassuppdata, hasdataavail, hasdatacitations, and hasassociateddata, which target papers containing data availability statements or supplemental data

files.”.

- Please clarify how the 11 dataset discovery platforms were chosen (line 243).
Authors: We followed your suggestion and clarified the criteria used to select the 11 dataset discovery platforms. Please refer to lines 335-342 in the revised version of the manuscript with track changes (or to lines 324-331 in the revised version without track changes): “The selection of the 11 dataset discovery resources was empirical and aimed at capturing, as far as possible, the heterogeneity and fragmentation of the current landscape. We therefore included different categories of resources: a web crawler (Google Dataset Search), a data aggregator (DataMed), domain-specific repositories (Epidemiologie – France and TEDI), a data-focused journal (Scientific Data), a bibliometric database (Web of Science), a data platform (OpenAIRE), a trusted data portal (FAIRSharing), as well as widely used general-purpose repositories (Dryad and Zenodo) and a repository catalog (Re3Data). Whenever possible, we preferentially selected resources that were openly accessible or freely available.”.

- Consider mentioning the CARE Principles as another ethical consideration. The CARE Principles were created by Native Americans to help dictate how research data generated from indigenous populations should be governed and made available.
Authors: Thank you for your relevant comment. We followed your suggestion by including the CARE Principles as an additional example of ethical consideration.
Please refer to lines 269-274 in the revised version of the manuscript with track changes (or to lines 258-264 in the revised version without track changes): “The central question at this stage is whether, and under what conditions, a dataset can be legally and ethically reused. For instance, the CARE Principles were created by Native Americans to guide how research data generated from Indigenous populations should be governed and made available [68-70].”.
Please also refer to lines 653-656 in the revised version of the manuscript with track changes (or to lines 597-600 in the revised version without track changes): “In addition, operationalizing the FAIR Principles alongside other standards, such as the CARE Principles, can enhance machine actionability while also ensuring that data are used appropriately across the entire data lifecycle [68]”.

- Around Line 380, you mention that Web of Science allows for filtering for data papers. Another existing connection between literature databases and data is the ability to filter PubMed Central results to papers that have associated data either described in a Data Availability or included in a supplemental file.
Authors: You are right, thank you. We provided more details to acknowledge this point. Please refer to lines 658-668 in the revised version of the manuscript with track changes (or to lines 601-609 in the revised version without track changes): “Integrating dataset search tools with bibliographic databases and data management platforms represents a promising step toward improving the findability of datasets [34]. For instance, databases such as Web of Science and PubMed enable users to filter search results specifically for data papers. This can be achieved via a dedicated filter box in Web of Science or by appending searches with the term data[filter] in PubMed. However, this functionality is not yet available in other bibliographic databases. In contrast, some literature repositories like PubMed Central offer more sophisticated search capabilities that allow users to limit queries to articles associated with data. This can be done by utilizing specialized search operators, including hassuppdata, hasdataavail, hasdatacitations, and hasassociateddata, which target papers containing data availability statements or supplemental data files.”.

- You mentioned DataMed on Line 187. I do not think DataMed is regularly maintained at this point and you may not want to include it. For example, it does not allow you to search for data past 2024, and it only has 2 datasets from 2024.
Authors: We appreciate your comment regarding the current status of DataMed. The most recent update on its website is from November 2023. DataMed was initially funded by the NIH through the Big Data to Knowledge (BD2K) program as part of the BioCADDIE (Biomedical and HealthCAre Data Discovery and Indexing Engine) project. As this funding has now ended, no new records appear to have been added since 2024. Although DataMed is no longer actively maintained, we think that it still provides useful insights into dataset discovery strategies. To reflect this limitation, we added clarifying sentences in the discussion section.
Please refer to lines 532-534 in the revised version of the manuscript with track changes (or to lines 496-498 in the revised version without track changes): “Without sustained funding and community support,

data search tools risk becoming outdated or abandoned, which compromises their reliability and long-term utility for researchers. For example, DataMed has not received new additions since 2024 [62,63,82,83].".

- Similar to the note above, another challenge worth mentioning is that many data infrastructure projects are reliant on grant funding, meaning that some data catalogs or data search tools go out of date or are no longer maintained.
Authors: You are right. We have expanded the discussion section to explicitly address this important challenge. Please refer to lines 530-534 in the revised version of the manuscript with track changes (or to lines 494-498 in the revised version without track changes): "Sustainability poses an additional systemic risk. Many data-sharing platforms lack long-term funding models, which threatens not only the durability and accessibility of datasets but also the continued relevance of the information they host [Kilgus et al. 2024]. Without sustained funding and community support, data search tools risk becoming outdated or abandoned, which compromises their reliability and long-term utility for researchers. For example, DataMed has not received new additions since 2024 [62,63,82,83].".

- In Table 1, you mention the NYU Data Catalog. It is not a government data portal. It is a data catalog that includes some government data.
Authors: You are right, we apologize for this mistake. We modified Table 1 accordingly. Please refer to Table 1.

Thank you for the opportunity to read your paper.

Editorial Manager®

Cookie Preference Center

We use cookies which are necessary to make our site work. We may also use additional cookies to analyze, improve and personalize our content and your digital experience. For more information, see our Cookie Policy.

You may choose not to allow some types of cookies. However, blocking some types may impact your experience of our site and the services we are able to offer. See the different category headings below to find out more or change your settings.

Allow all

Manage Consent Preferences

Strictly Necessary Cookies

Always active

These cookies are necessary for the website to function and cannot be switched off in our systems. They are usually only set in response to actions made by you which amount to a request for services, such as setting your privacy preferences, logging in or filling in forms. You can set your browser to block or alert you about these cookies, but some parts of the site will not then work.

Functional Cookies

These cookies enable the website to provide enhanced functionality and personalisation. They may be set by us or by third party providers whose services we have added to our pages. If you do not allow these cookies then some or all of these services may not function properly.

Performance Cookies

These cookies allow us to count visits and traffic sources so we can measure and improve the performance of our site. They help us to know which pages are the most and least popular and see how visitors move around the site.

Targeting Cookies

These cookies may be set through our site by our advertising partners. They may be used by those companies to build a profile of your interests and show you relevant adverts on other sites. If you do not allow these cookies, you will experience less targeted advertising.

Cookie List

Clear

Apply Cancel
Consent Leg.Interest

Confirm my choices

Powered by onetrus
