# Supplementary material for: Datagraphy: toward a systematic approach to dataset discovery
Source: Gigascience. 2025 Oct 22;14:giaf134. doi: 10.1093/gigascience/giaf134 (PMC12644982; doi:10.1093/gigascience/giaf134)
Supplement: giaf134_Reviewer_1_Report_Original_Submission — Jasper Jan Koehorst -- 7/13/2025 [file giaf134_reviewer_1_report_original_submission.pdf]

Reviewer Report

Title: Datagraphy: toward a systematic approach to dataset discovery

Version: Original Submission **Date:** 7/13/2025

Reviewer name: Jasper Jan Koehorst

Reviewer Comments to Author:

Dear Authors,The concept that was proposed in this manuscript, datagraphy, makes it clear that there are real problems with the way current datasets are published through "generic" data repositories. The proposed framework shows a real and growing problem regarding inefficient and inconsistent dataset discovery. This problem however is in this approach tackled or perhaps bandaged by proposing a structured method toward the researcher. Although the nine steps mentioned, it remains an intensive exercise and when in a popular field would need to be done frequently to enable the discovery of new datasets.This proposed framework however does not propose any approach to tackle the real problems. Which is the freedom data repositories have to implement their own procedures and methods for data discovery. Without this focus this sounds more like a bandaid than a solution towards the problem that is being tried to address here. In addition researchers that search for data often also deposit datasets. It might be a more, in the long term, solution if standards are proposed that aid in the discovery of datasets through standardised metadata templates that are submitted alongside the datasets such as in the form of Research Objects or RO-crates as they are essentially a lightweight approach to packaging research data with their metadata.Overall I think the approach has merit but it does not tackle the real issue at hand.

Level of Interest

Please indicate how interesting you found the manuscript: Choose an item.

Quality of Written English

Please indicate the quality of language in the manuscript: Choose an item.

Declaration of Competing Interests

Please complete a declaration of competing interests, considering the following questions:

- Have you in the past five years received reimbursements, fees, funding, or salary from an organisation that may in any way gain or lose financially from the publication of this manuscript, either now or in the future?
- Do you hold any stocks or shares in an organisation that may in any way gain or lose financially from the publication of this manuscript, either now or in the future?

- Do you hold or are you currently applying for any patents relating to the content of the manuscript?
- Have you received reimbursements, fees, funding, or salary from an organization that holds or has applied for patents relating to the content of the manuscript?
- Do you have any other financial competing interests?
- Do you have any non-financial competing interests in relation to this paper?

If you can answer no to all of the above, write 'I declare that I have no competing interests' below. If your reply is yes to any, please give details below.

I declare that I have no competing interests

I agree to the open peer review policy of the journal. I understand that my name will be included on my report to the authors and, if the manuscript is accepted for publication, my named report including any attachments I upload will be posted on the website along with the authors' responses. I agree for my report to be made available under an Open Access Creative Commons CC-BY license (http://creativecommons.org/licenses/by/4.0/). I understand that any comments which I do not wish to be included in my named report can be included as confidential comments to the editors, which will not be published.

Choose an item.

To further support our reviewers, we have joined with Publons, where you can gain additional credit to further highlight your hard work (see: https://publons.com/journal/530/gigascience). On publication of this paper, your review will be automatically added to Publons, you can then choose whether or not to claim your Publons credit. I understand this statement.

Yes Choose an item.
