# Supplementary material for: Datagraphy: toward a systematic approach to dataset discovery
Source: Gigascience. 2025 Oct 22;14:giaf134. doi: 10.1093/gigascience/giaf134 (PMC12644982; doi:10.1093/gigascience/giaf134)
Supplement: giaf134_Reviewer_2_Report_Original_Submission — Nicole Contaxis -- 7/16/2025 [file giaf134_reviewer_2_report_original_submission.pdf]

Reviewer Report

Title: Datagraphy: toward a systematic approach to dataset discovery

Version: Original Submission **Date:** 7/16/2025

Reviewer name: Nicole Contaxis

Reviewer Comments to Author:

This paper addresses an important topic, and I suggest that it be published with some revisions. I have included some areas for revision below:- Re3Data is a listing of data repositories, not a data repository itself. For example, if I search for 'cancer' in Re3Data, I will be shown data repositories that specialize in cancer, not datasets on cancer. In your example on Line 275, please clarify what the three resources you located in Re3Data and if they were datasets or data repositories. If you located a dataset by searching a repository you located through Re3Data, please clarify that as well.- Similarly FAIRSharing is a listing of data repositories (referred to as databases on their site), data policies, and data standards. Please clarify in your example on Line 275 what 10 resources your located in FAIRSharing and if they were data repositories, data policies, or data standards. If you located a dataset by searching a repository you located through FAIRSharing, please clarify that as well.- Missing in your overall workflow is ways to locate dataset contained in supplementary materials. PubMed Central, for example, allows users to limit searches to papers that have associated data (either in a Data Availability Statement or in the supplemental files). I understand if you decide not to include that type of search in your PRISMA-like workflow, but you should at least mention that the workflow will not locate those types of materials.- Please clarify how the 11 dataset discovery platforms were chosen (line 243).- Consider mentioning the CARE Principles as another ethical consideration. The CARE Principles were created by Native Americans to help dictate how research data generated from indigenous populations should be governed and made available.- Around Line 380, you mention that Web of Science allows for filtering for data papers. Another existing connection between literature databases and data is the ability to filter PubMed Central results to papers that have associated data either described in a Data Availability or included in a supplemental file.- You mentioned DataMed on Line 187. I do not think DataMed is regularly maintained at this point and you may not want to include it. For example, it does not allow you to search for data past 2024, and it only has 2 datasets from 2024.- Similar to the note above, another challenge worth mentioning is that many data infrastructure projects are reliant on grant funding, meaning that some data catalogs or data search tools go out of date or are no longer maintained.- In Table 1, you mention the NYU Data Catalog. It is not a government data portal. It is a data catalog that includes some government data.Thank you for the opportunity to read your paper.

Level of Interest

Please indicate how interesting you found the manuscript: Choose an item.

Quality of Written English

Please indicate the quality of language in the manuscript: Choose an item.

Declaration of Competing Interests

Please complete a declaration of competing interests, considering the following questions:

- Have you in the past five years received reimbursements, fees, funding, or salary from an organisation that may in any way gain or lose financially from the publication of this manuscript, either now or in the future?
- Do you hold any stocks or shares in an organisation that may in any way gain or lose financially from the publication of this manuscript, either now or in the future?
- Do you hold or are you currently applying for any patents relating to the content of the manuscript?
- Have you received reimbursements, fees, funding, or salary from an organization that holds or has applied for patents relating to the content of the manuscript?
- Do you have any other financial competing interests?
- Do you have any non-financial competing interests in relation to this paper?

If you can answer no to all of the above, write 'I declare that I have no competing interests' below. If your reply is yes to any, please give details below.

I have no financial competing interests. I am cited in the references twice.

I agree to the open peer review policy of the journal. I understand that my name will be included on my report to the authors and, if the manuscript is accepted for publication, my named report including any attachments I upload will be posted on the website along with the authors' responses. I agree for my report to be made available under an Open Access Creative Commons CC-BY license (http://creativecommons.org/licenses/by/4.0/). I understand that any comments which I do not wish to be included in my named report can be included as confidential comments to the editors, which will not be published.

Choose an item.

To further support our reviewers, we have joined with Publons, where you can gain additional credit to further highlight your hard work (see: https://publons.com/journal/530/gigascience). On publication of this paper, your review will be automatically added to Publons, you can then choose whether or not to claim your Publons credit. I understand this statement.

Yes Choose an item.
