# Supplementary material for: Datagraphy: toward a systematic approach to dataset discovery
Source: Gigascience. 2025 Oct 22;14:giaf134. doi: 10.1093/gigascience/giaf134 (PMC12644982; doi:10.1093/gigascience/giaf134)
Supplement: giaf134_Reviewer_2_Report_Revision_1 — Nicole Contaxis -- 10/6/2025 [file giaf134_reviewer_2_report_revision_1.pdf]

**Reviewer Report**

**Title: Datagraphy: toward a systematic approach to dataset discovery**

**Version: Revision 1** **Date:** 10/6/2025

**Reviewer name: Nicole Contaxis**

**Reviewer Comments to Author:**

Thank you for your response to my comments. I believe you have addressed my concerns in your edits. I have one final note on line 335. You mention that your selection of the 11 dataset discovery resources was empirical as well as designed to capture the heterogeneity of the current data discovery landscape. While I recognize that your selection does reflect the heterogeneity of the landscape, I am not sure it was empirical. For example, when representing general-purpose repositories, you chose Dryad and Zenodo. If this were an empirical selection, I might expect an explanation why other general-purpose repositories weren't chosen (e.g., Figshare). I do not think that this is sufficient enough of a concern for another round of edits, but I am not sure I would call this selection empirical.

**Level of Interest**

Please indicate how interesting you found the manuscript: Choose an item.

**Quality of Written English**

Please indicate the quality of language in the manuscript: Choose an item.

**Declaration of Competing Interests**

Please complete a declaration of competing interests, considering the following questions:

- Have you in the past five years received reimbursements, fees, funding, or salary from an organisation that may in any way gain or lose financially from the publication of this manuscript, either now or in the future?
- Do you hold any stocks or shares in an organisation that may in any way gain or lose financially from the publication of this manuscript, either now or in the future?
- Do you hold or are you currently applying for any patents relating to the content of the manuscript?
- Have you received reimbursements, fees, funding, or salary from an organization that holds or has applied for patents relating to the content of the manuscript?
- Do you have any other financial competing interests?
- Do you have any non-financial competing interests in relation to this paper?

If you can answer no to all of the above, write 'I declare that I have no competing interests' below. If your reply is yes to any, please give details below.

I am cited in the paper and a program I run, the NYU Data Catalog, is mentioned. I do not financially benefit from citations or mentions of that program.

I agree to the open peer review policy of the journal. I understand that my name will be included on my report to the authors and, if the manuscript is accepted for publication, my named report including any attachments I upload will be posted on the website along with the authors' responses. I agree for my report to be made available under an Open Access Creative Commons CC-BY license (http://creativecommons.org/licenses/by/4.0/). I understand that any comments which I do not wish to be included in my named report can be included as confidential comments to the editors, which will not be published.

Choose an item.

To further support our reviewers, we have joined with Publons, where you can gain additional credit to further highlight your hard work (see: https://publons.com/journal/530/gigascience). On publication of this paper, your review will be automatically added to Publons, you can then choose whether or not to claim your Publons credit. I understand this statement.

Yes Choose an item.
